# Comparative characterization of human induced pluripotent stem cells (hiPSC) derived from patients with schizophrenia and autism

**DOI:** 10.1038/s41398-019-0517-3

**Published:** 2019-07-29

**Authors:** Lena-Marie Grunwald, Ricarda Stock, Kathrina Haag, Sandra Buckenmaier, Mark-Christian Eberle, Dirk Wildgruber, Helena Storchak, Martin Kriebel, Stephanie Weißgraeber, Lisha Mathew, Yasmin Singh, Maarten Loos, Ka Wan Li, Udo Kraushaar, Andreas J. Fallgatter, Hansjürgen Volkmer

**Affiliations:** 1 Department Molecular Biology, NMI Natural and Medical Sciences Institute at the University of Tübingen, Markwiesenstr. 55, 72770 Reutlingen, Germany; 2 Department Cell Physiology, NMI Natural and Medical Sciences Institute at the University of Tübingen, Markwiesenstr. 55, 72770 Reutlingen, Germany; 3 Department of Psychiatry, University of Tübingen, Osianderstrasse 24, 72076 Tübingen, Germany; 4 CeGaT GmbH - Center for Genomics and Transcriptomics, Paul-Ehrlich-Str. 23, 72076 Tübingen, Germany; 5 Sylics (Synaptologics BV), PO Box 71033, 1008 BA Amsterdam, The Netherlands; 6 Department of Molecular and Cellular Neurobiology, Center for Neurogenomics and Cognitive Research, Amsterdam Neuroscience, Vrije Universiteit, Amsterdam, The Netherlands

**Keywords:** Stem cells, Neuroscience

## Abstract

Human induced pluripotent stem cells (hiPSC) provide an attractive tool to study disease mechanisms of neurodevelopmental disorders such as schizophrenia. A pertinent problem is the development of hiPSC-based assays to discriminate schizophrenia (SZ) from autism spectrum disorder (ASD) models. Healthy control individuals as well as patients with SZ and ASD were examined by a panel of diagnostic tests. Subsequently, skin biopsies were taken for the generation, differentiation, and testing of hiPSC-derived neurons from all individuals. SZ and ASD neurons share a reduced capacity for cortical differentiation as shown by quantitative analysis of the synaptic marker PSD95 and neurite outgrowth. By contrast, pattern analysis of calcium signals turned out to discriminate among healthy control, schizophrenia, and autism samples. Schizophrenia neurons displayed decreased peak frequency accompanied by increased peak areas, while autism neurons showed a slight decrease in peak amplitudes. For further analysis of the schizophrenia phenotype, transcriptome analyses revealed a clear discrimination among schizophrenia, autism, and healthy controls based on differentially expressed genes. However, considerable differences were still evident among schizophrenia patients under inspection. For one individual with schizophrenia, expression analysis revealed deregulation of genes associated with the major histocompatibility complex class II (MHC class II) presentation pathway. Interestingly, antipsychotic treatment of healthy control neurons also increased MHC class II expression. In conclusion, transcriptome analysis combined with pattern analysis of calcium signals appeared as a tool to discriminate between SZ and ASD phenotypes in vitro.

## Introduction

Schizophrenia (SZ) is a serious disease with a prevalence of 1% and high social burden^1,2^. Beside the elaboration of positive (delusions, perceptual disturbances, and hallucinations) and negative symptoms (e.g., lethargy, blunted emotional responses, reductions in speech, social withdrawal, anhedonia, and alogia) patients suffer from increased risk for drug abuse and comorbid depression resulting in suicide in about 5–10% of the cases^3^. Although therapeutic approaches were considerably improved over the last century, a disease modifying and sufficient therapy is not available especially in the case of negative symptoms and cognitive impairments^4^. SZ is a complex disease of unknown etiology, while 145 genetic susceptibility genes of low penetrance have been identified^5,6^. Linking genomic alterations in SZ to single cell sequencing data, recent hypotheses suggest the involvement of e.g., pyramidal neurons, medium spiny neurons, and subclasses of interneurons^7^. For the understanding of disease mechanisms and the development of therapeutic approaches, hiPSC give access to experimental models that reproduce the genetic background of patients.

As reported previously, SZ-hiPSCs showed diminished neuronal connectivity, decreased neurite number, PSD95-protein levels and glutamate receptor expression, and deficient synaptic function^8–12^. On the other hand, hiPSC modelling autism spectrum disorders exhibited activity-dependent dendritic retraction in Timothy syndrome or dysfunctional glutamatergic signalling, as well as Na^+^ and inactivating K^+^ voltage-gated currents in idiopathic autism samples^13,14^. An important problem is the availability of models and readouts that ensure discrimination among different neurodevelopmental disorders. Therefore, hiPSC-derived neurons from healthy control individuals as well as from patients with SZ and autism were tested in a panel of assays, including quantification of synaptic markers, measurement of neurite outgrowth, analysis of calcium signals as well as transcriptome analysis. Analysis of calcium signals and transcriptomic profiles proved to be a possibility to discriminate SZ models from healthy controls and autism samples.

## Patients, materials, and methods

### Patient recruitment and diagnostic procedures

After a positive vote of the local Institutional Review Board and based on extensive screening of the files of patients with SZ, personal psychiatric and psychopathological investigations, and checking of inclusion and exclusion criteria, six individuals (three patients with SZ, three patients with autism) were recruited and investigated according to diagnostic criteria of Diagnostic and Statistical Manual of Mental Disorders (DSM IV)^15^. Three healthy individuals were included for comparison. For inclusion and exclusion criteria as well as medications see Supplemental methods.

### hiPSC generation and neuronal differentiation

Human skin biopsies were used for the generation of hiPSC via retroviral transduction^16^. For NPC generation, hiPSCs were enzymatically dissociated and seeded into AggreWell^TM^ plates (STEMCELL Technologies Germany GmbH, Köln, Germany) for embryoid body formation. Subsequently, neural rosettes were induced, selected, and differentiated into neural progenitor cells (NPCs), which were finally differentiated into neurons. For further details see Supplemental methods.

### Transcriptomic analysis

For transcriptome sequencing, 4 × 10^4^ cells were lysed into RLT buffer for total RNA extraction (RNeasy Mini Kit; Qiagen GmbH, Hilden, Germany). The quality and quantity of RNA was measured using the RNA 6000 Nano chip on the 2100 BioAnalyzer (Agilent Technologies, Santa Clara, USA). In total 100 ng of total RNA was used for library preparation with the TruSeq Stranded mRNA LT Kit (Illumina) according to manufacturer’s protocols. Massively parallel sequencing was performed on the HiSeq 2500 (Illumina) with single-end sequencing (100 bp read length) to yield an average of 75 million reads per sample. Sequencing data were processed with CASAVA (version 1.8.2, Illumina) to demultiplex sequencing reads. Sequencing adapters were trimmed using Skewer (version 0.1.116^17^). Trimmed raw reads were aligned to the human reference genome (hg19) using STAR (version 2.4.0 h^18^) with default parameters. Mapped reads were counted with HTSeq-count (Anders et al. 2015). Analyses of differential expression between groups were performed with DESeq2 (Love et al. 2014) in R (R Core Team 2015). Differentially expressed genes were identified at a significance threshold of *p* < 0.01 (FDR-corrected *p*-values). When comparing the transcriptional effects of the different treatments, a more lenient *p*-value threshold was employed to ensure that observed differences did not result from marginally insignificant *p*-values due to experimental noise. A gene was considered as significantly deregulated if *p* < 0.01 was achieved at least in one of three comparisons and if any other comparison showed differential expression with *p* < 0.05. Gene ontology classification was performed with the help of www.pantherdb.org^19^.

### Quantification of neurite outgrowth and PSD95 clusters

After predifferentiation using 3 N medium supplemented with bFGF, 1 × 10^5^ cells/well were differentiated on Poly-L-ornithine-laminin coated µ-plates with 24 wells (ibidi GmbH, Planegg/Martinsried, Germany) for 4 days. Cells were fixed using 4% PFA and stained against β-III-tubulin. In case of drug treatment cells were treated with antipsychotics after 24 h, cultivated again for 48 h and fixed. As antipsychiotics Haloperidol, Olanzapine, and Clozapine (all Sigma-Aldrich, St. Louis, USA) were used in concentrations up to 4 µM. DMSO served as solvent control.

Confocal fluorescence images of neuronal cultures were further processed and analyzed using Imaris software (Bitplane AG, Zurich, Switzerland). PSD95-positive spots colocalizing with β-III-tubulin-stained neurites were quantified on neuritic segments 20 µm in length. Each experiment was normalized to CTR1. Within each experiment, all settings for exposure times, contrast, brightness, resolution, and threshold values were kept constant.

### Calcium imaging

For calcium imaging, NPCs were incubated for 56 days in 3 N medium^20^ for cortical-like differentiation. For further 14 days of differentiation, media were exchanged for BrainPhys^TM^ Neuronal Medium (STEMCELL Technologies Germany GmbH, Köln, Germany) plus supplements (20× NeuroCult SM1 Supplement, 10× N2 Supplement, 100 µg/ml BDNF, 100 µg/ml GDNF, 100 mg/ml cAMP, and 50 mg/ml L-ascorbic acid). For visualization of calcium signals, neurons were stained with 1 µM Cal520^®^ AM (AAT Bioquest^®^, CA, USA) for 30 min and imaged for 10 min at a rate of 20 frames per second using spinning disc confocal microscopy (Cell Observer SD, Carl Zeiss Microscopy GmbH, Oberkochen, Deutschland equipped with an an iXon DV885 EMCCD camera, Andor Technology, Belfast, UK).

### Statistic evaluation

Statistical analyses were performed using StatView or JMP ^®^ 10 software (SAS Institute Inc., North Carolina, USA) and GraphPad InStat (Version 3.10, GraphPad Software). The *p*-values were assigned as follows: *p < 0.05; **p < 0.01; ***p < 0.001. Each experiment was repeated at least three times providing at least three independent iPSC differentiations.

## Results

### Recruitment of patients with schizophrenia and autism

**Fig. 1:**
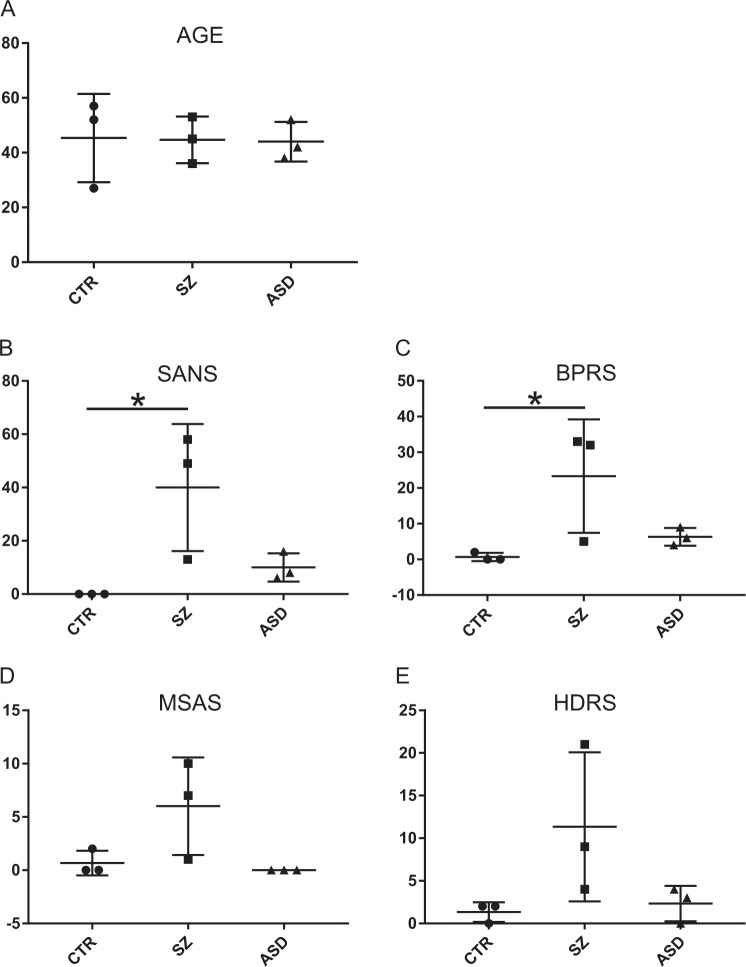
Psychiatric evaluation. Scale for the Assessment of Negative Symptoms (SANS), Brief Psychiatric Rating Scale (BPRS), Hamilton Depression Rating Scale (HDRS), and Modified Simpson Angus Scale (MSAS). *N* = 3 for each experimental group healthy controls (CTR), schizophrenia (SZ), and autism (ASD). SANS: CTR vs. SZ, *H*(2,9) = 6.713; *p* = 0.035. BPRS: CTR vs. SZ, *H*(2,9) = 6.006; *p* = 0.05. *p < 0.05) all other pairwise comparisons were not significant (*α* = 0.05)

Besides DSM IV scores, a positive family history for SZ with an affected first degree relative was an essential criterion for the selection of patients with SZ. The patients with ASD and the healthy controls did not have a family history for any psychiatric disorder. All three groups (patients with schizophrenia, ASD, and healthy controls) consisted of one women and two men. Patients and healthy controls were age-matched but not the patients with autism (Fig. 1a). All patients and healthy controls were investigated by the Scale for the Assessment of Negative Symptoms (SANS)^21^, brief psychiatric rating scale (BPRS)^22^, Hamilton Depression Rating Scale (HDRS)^23^, and Modified Simpson Angus Scale (MSAS; EPS)^24^. Only in BPRS and SANS the group of patients with SZ significantly differed from healthy individuals (Fig. 1b, c) while no differences between patients with ASD and healthy individuals were observed. Likewise, no significant differences appeared among the three groups after application of HDRS for criteria of depression and MSAS (Fig. 1d, e). Therefore, negative symptoms appeared as the most obvious difference between the patients diagnosed with SZ and the healthy controls.

### Generation of hiPSCs and hiPSC-derived neurons

For further experimentation, hiPSC were generated from dermal fibroblasts received from patiens with SZ as well as of healthy individuals and ASD patients. hiPSC were controlled for stem cell phenotypes, pluripotency, and genomic stability (see Supplementary Figs. S1, 2 and supplemental methods for details and quality controls). hiPSC were first differentiated into NPC which were then terminally differentiated into neurons with a cortical phenotype since they were considered to reflect negative symptoms relying on hypofrontality^25–27^. All NPC and corresponding differentiated neurons were examined for marker expression specific for neural progenitors and differentiated neurons (see Supplementary Figs. S3, 4).

### Developmental phenotypes

**Fig. 2:**
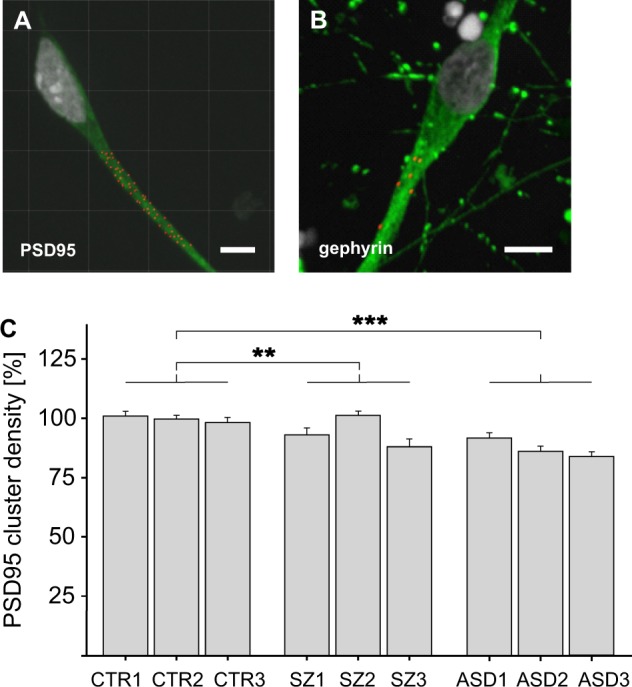
Expression of differentiation markers in hiPSC-derived neurons. **a**, **b** Immunostaining for PSD95 or gephyrin clusters, respectively. PSD95 or gephyrin clusters are indicated in red while neurons were stained for β-III-tubulin in green. Scale bar 10 µm. **c** Determination of PSD95 cluster density on 20 µm segments on β-III-tubulin-positive neurites. For each of the three individuals of each group (healthy CTR1-3, SZ1-3, and ASD1-3), one clone was tested in at least six independent differentiations. Within one experiment all donors were normalized to CTR1. For a group comparison, mean PSD95 cluster density of all donors of each group was calculated and compared with the other groups. Kruskal–Wallis Test and Dunn’s post hoc test, *H*(2) = 62.98; **p < 0,01; ***p < 0,001; *n* = 360 for each group, error bars are s.e.m.). Data were collected from three independent experiments

SZ as well as autism are recognized as neurodevelopmental disorders characterized by deficits in neuronal connectivity^28^. Therefore, hiPSC differentiation into neuronal phenotypes may represent an appropriate tool to study developmental impairments found in SZ and ASD. After neuronal differentiation of NPC for 56 days, cultures were stained for neuronal and synaptic markers. Overall, the culture showed poor expression of inhibitory synapse marker gephyrin as compared with pronounced PSD95 staining indicative for excitatory postsynaptic structures (Fig. 2a, b). Twenty micrometer segments of β-III-tubulin-positive neurites were chosen for the determination of PSD95 excitatory marker densities (Imaris software package, Bitplane). The group comparison between neurons from the SZ and the healthy control group (Fig. 2c) revealed a small reduction in PSD95 spot densities by 5% that was even more pronounced for neurons derived from individuals with autism (12%). Likewise, PSD95 spot densities were also significantly lower in the ASD samples when compared with the SZ samples. Thus, at the level of PSD95 marker expression, a minor, albeit significant reduction of PSD95 marker density was observed for SZ and ASD neurons. Since this analysis may detect extrasynaptic PSD95 clusters, colocalizing PSD95 and VGlut cluster spots indicative for synapses formed in vitro were additionally examined. Here, significant differences were only observed for the autism group compared with healthy controls (Supplementary Fig. S5).

**Fig. 3:**
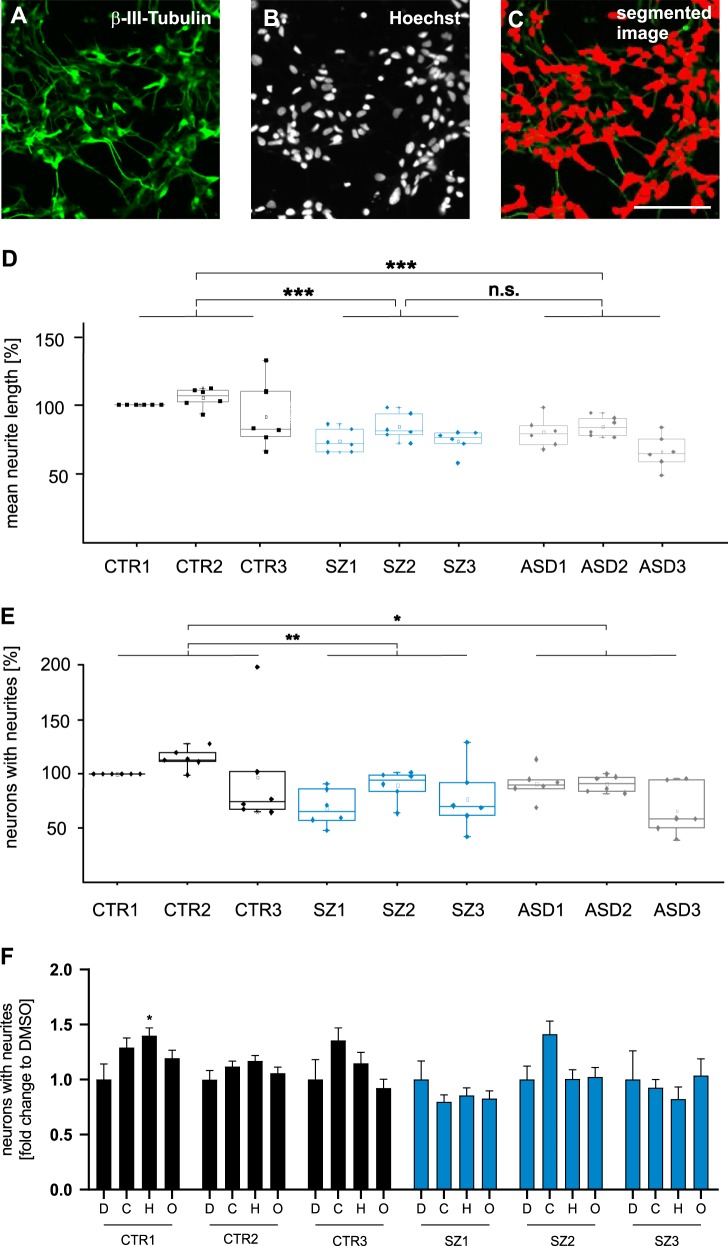
Determination of neurite outgrowth. **a**–**c** β-III-tubulin- (**a**) and Hoechst-stained (**b**) neuronal cultures were segmented (**c**) using the MetaXpress software (ImageXpress, Molecular Devices). Scale bar 100 µm. **d**, **e** Mean neurite length and the percentage of neurons with neurites was determined by dividing the lengths of individual neuritis >10 µm by the number of Hoechst-stained nuclei. Within individual experiments, mean neurite length was normalized to donor CTR1. For each clone (CTR1-3, SZ1-3, and ASD1-3) with one clone per individual, six independent differentiations were included for outgrowth measurements. Mean neurite length was determined from 25 images, each with at least 50 cells analysed, and represented as a dot in the box-and-whisker plots. Group comparisons included the three individual clones of all experiments *n* = 18; Kruskal–Wallis Test and Dunn’s post hoc test, *H*(2) = 21.6; ***p < 0.001, error bars are s.e.m.; n.s. not significant ***p < 0.001. **f** Drug response of neurons exposed to antipsychotic drugs. Drug response is expressed as the *n*-fold change in the percentage of neurons with neurites normalized to the DMSO control (“D”) for each clone, 1 µM clozapine (“C”), 1 µM haloperidol (“H”), and 1 µM olanzapine (“O”). Experiments were performed with three clones from CTR1, three clones from CTR2, and three clones from SZ2. Percentage of neurons with neurites was determined from 81 images retrieved from at least three independent experiments. Kruskal–Wallis Test and Dunn’s post hoc test. CTR1 *H*(3) = 8.31, CTR2 *H*(3) = 3.66, CTR3 *H*(3) = 7.83, SZ1 *H*(3) = 0.76, SZ2 *H*(3) = 7.96, and SZ3 *H*(3) = 35.6. *p < 0.5;**p < 0.01; ***p < 0.001; error bars are s.e.m

Neurodevelopmental deficits in SZ or ASD may correspond to a decreased capacity for neurite outgrowth. To test this hypothesis, NPC were differentiated into neurons for four days and stained for β-III-tubulin and nuclei (Hoechst) to assess neurite outgrowth (Fig. 3a, b). Images were retrieved with an ImageXpress device and mean neurite outgrowth was determined by the MetaXpress software package in a fully automatized procedure (Fig. 3c, d). In a group comparison, neurons derived from patients with either SZ or autism showed significantly reduced neurite length by ~24% (SZ and ASD) as compared with the healthy controls, while no difference was observed between SZ and ASD samples. Likewise, the percentage of neurons elaborating neurites was analysed. Within the groups of CTR and SZ clones no significant differences were observed indicating a relatively homogenous induction of neurite outgrowth. In contrast, significant reductions were found in group comparisons between CTR and SZ as well as CTR and ASD samples (Fig. 3e). In summary, these results indicate reduced neuronal maturation and neurite outgrowth of SZ and ASD model neurons.

Next, the impact of different concentrations of antipsychotic drugs was tested by automatized high content analysis of neuronal networks formed in 72 h of incubation. Drugs were added to CTR1-3 and SZ1-3 after 24 h to allow for initial adhesion. Haloperidol was chosen as a first generation antipsychotic, while clozapine and olanzapine represent second generation antipsychotics. Drugs were applied at a concentration of 1 µM similar to a previous report as a first hint to determine the impact of antipsychotic treatment on the percentage of cells with neurites^9^. No significant changes were observed after drug application to any of the SZ clones as compared with the DMSO control. Clozapine, olanzapine, and haloperidol showed a minor tendency to increase the percentage of neurons with neurites in CTR clones, while statistical significance was achieved only with haloperidol treated CTR1 (see Fig. 3f). Measuring neurite length at the example of clones CTR1 and SZ2, a concentration-dependent decrease of neurite outgrowth was observed in CTR2 after application of haloperidol and olanzapine (Supplementary Information Fig. S6). In summary, drugs had only minor effects on iPSC-derived neurons under inspection.

### Impaired neuronal functioning in SZ

**Fig. 4:**
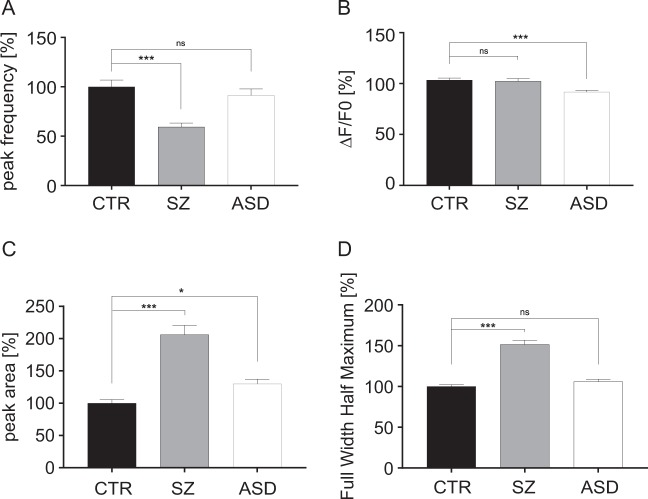
Measurement of calcium signals. Cal-520®-stained neurons of healthy control, schizophrenia, and autism groups were imaged for 10 min and signals obtained were used to calculate peak frequency (**a**), peak amplitude (**b**), peak area (**c**), and full width half maximum (**d**). Fluorescence intensity Δ*F*/*F*, frequency, and length of events were analyzed using Fiji and Origin software; Kruskal–Wallis Test and Dunn’s post hoc test, peak frequency *H*(2) = 22.56, Δ*F*/*F*0 *H*(2) = 19.44, peak area *H*(2) = 93.17, and FWHM *H*(2) = 45.2; *p < 0.01; ***p < 0.0001; for each group, error bars are s.e.m.

For analysis of calcium signals, NPC derived from all nine individuals (one clone per individual, three individuals for each group of healthy donors, SZ and ASD) were differentiated into neurons for 8 weeks. Cells were loaded with the calcium indicator Cal-520. Images of spontaneously active neurons were retrieved and calcium signals were analyzed for calculation of peak frequency, peak amplitude (Δ*F*/*F*0), peak area, and peak full width half maximum (FWHM, Fig. 4a–d). Compared with healthy controls, peak frequency was reduced in the SZ group, not observed with the autism group. The peak amplitude (Δ*F*/*F*0) was equal between healthy controls and the SZ group, while the autism group displayed a minor, however significant, decrease. Peak area and FWHM were considerably increased in the SZ group, while this effect was not observed with neurons derived from ASD individuals. Overall, calcium signals of different clones within one group showed small standard errors of the mean indicating a relatively homogenous response over different clones and patients. Thus, pattern analysis of calcium signals provides a mean to discriminate the healthy control from the SZ group. Most interestingly, calcium responses discriminated between the SZ and ASD groups that contrasts with comparable effects in PSD95 density and neurite outgrowth measurements.

### Transcriptomic analysis of neurons differentiated from hiPSC

**Fig. 5:**
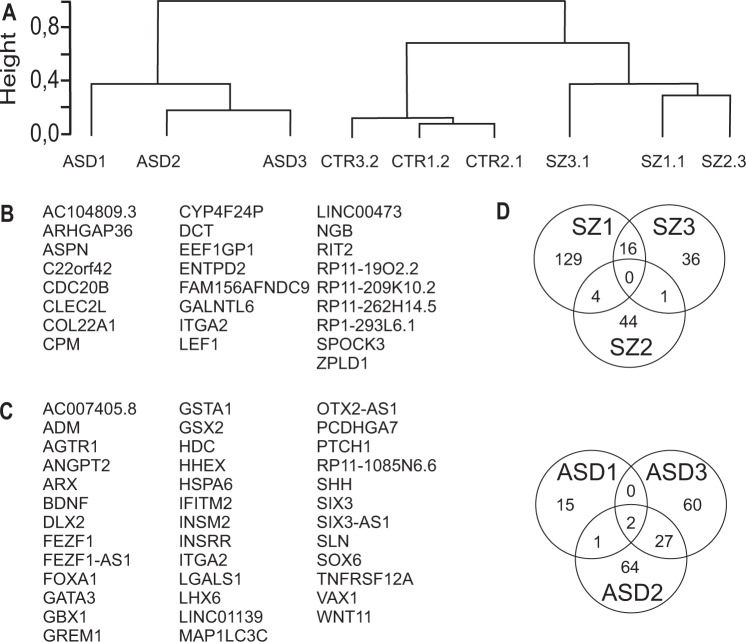
Transcriptome sequencing shows discrimination of disease groups. **a** Hierarchical clustering based on similarity of expression data (Spearman’s rank correlation coefficient) of deregulated genes in a one group comparison. **b**, **c** List of jointly enriched genes after comparison of the group of the three control and the three donors with SZ (**b**) or ASD (**c**), one clone per patient. **d** Heterogeneity within the schizophrenia and ASD cohort. Analysis of the transcriptome of individual schizophrenia samples with the group of the three healthy control samples. The Venn diagram depicts the number of individually and commonly deregulated genes in the three SZ and the three ASD samples, respectively

For further characterization, comparative transcriptome analysis was performed including nine clones (three individuals each for healthy control, schizophrenia, and autism) after 4 weeks of neuronal differentiation. This time point was chosen to identify disease-relevant genes in immature neurons for detection of neurodevelopmental phenotypes of SZ. All genes of all samples, expressed significantly different in a one- group comparison, were submitted to hierarchical clustering based on similarity of expression data (Spearman’s rank correlation coefficient, Fig. 5a). The analysis shows that the different groups (healthy, schizophrenia, and autism) can be discriminated according to their transcriptomic profiles. Likewise, a group comparison identified genes jointly deregulated in the three schizophrenia and in the autism samples compared with the control samples CTR1-3 (Fig. 5b, c, Supplementary Table S1). Only a small number of 25 deregulated genes was found suggesting a low overlap in the SZ transcriptomic profiles (*α* = 0.05). Nevertheless, LEF1 was identified suggesting deficits in the WNT signalling pathway across all patients in accordance with a previous report^29^. Likewise, another hit is represented by ENTPD2 that was found to be regulated by antipsychotic treatment of human patients^30^. Comparison of samples ASD1-3 with healthy controls revealed 38 significantly deregulated genes. Among these genes, we identified HDC that was recently shown to be deregulated in patients with ASD^31^. Likewise, we found sonic hedgehog (SHH), its receptor ptch1 as well as the downstream SHH target Grem1^32,33^. Interestingly, integrin α2 becomes deregulated in SZ and ASD samples. The relatively small number of deregulated mRNAs prompted us to get more insight into the individual disposition. Each of the SZ and ASD samples were compared with the group of healthy controls, individually. Comparison of the transcriptomic profile revealed that transcriptomes of the three patients with schizophrenia or ASD shared only few deregulated genes (Fig. 5d). In the case of ASD samples FEZ-F1 and FEZF1-AS1 were found in all three samples. This indicates that the SZ and ASD individuals were quite heterogeneous in the comparison of significantly deregulated genes within groups although all three groups (CTR, SZ, and ASD) clearly segregate in hierarchical clustering.

In case of patient SZ2, 48 genes were deregulated in comparison with the group of healthy controls (*α* = 0.05; see Supplementary Table S2). Analysis of the reactome by Panther gene classification (http://pantherdb.org) revealed that patient SZ2 showed deregulated immune system-associated genes when compared with the group of healthy controls (Supplementary Fig. S7) that was not observed in SZ1 and 3. Panther gene classification revealed significant enrichment of genes attributed to MHC class II antigen presentation (FDR 6.91E–12; Supplementary Fig. S7). In addition to the genes *HLA-DPA1*, *HLA-DRB1*, and *CD74*, found to be deregulated in neuropsychiatric disorders^34^, we identified further members of the MHC class II processing and presentation pathway including *CIITA, CD74, HLA-DOA, HLA-DRA, HLA-DRB5, HLA-DRB6, HLA-DQA1, HLA-DQA2, HLA-DQB2, HLA-DMA*, and *HLA-DMB* (Supplementary Table S2). The class II major histocompatibility complex transactivator *CIITA* activates MHC class II promoters. CD74 acts as an MHC class II chaperone and directly associates with the MHC class II α and β chains in the endoplasmic reticulum^35^. HLA-DOA is involved in antigen loading of MHC class II complexes in the endosomes^36^, while the remaining MHC class II-associated genes are involved in antigen presentation.

Patient SZ2 who has shown upregulated MHC class II expression received clozapine alone as a treatment, while other drugs or additional drugs were prescribed for SZ1 and SZ3. To test for a potential impact of clozapine on transcriptomic profiles, the impact of clozapine on healthy control neurons CTR1 derived from an individual without antipsychotic treatment was examined. After having passed neuronal differentiation for 4 weeks, CTR1 neurons were exposed to clozapine for 2 weeks. Analysis of significantly deregulated genes in DMSO-treated control samples versus clozapine-treated samples again revealed genes involved in MHCII antigen presentation (FDR 2.10E–08; Supplementary Fig. S8). In conclusion, expression of genes related to MHC class II antigen presentation were deregulated in one patient SZ2 and were susceptible to clozapine treatment in the healthy control.

### Proteomic analysis of neurons differentiated from hiPSC

Neurons were collected after 6 weeks of differentiation for the isolation of proteins and comparative proteomic analysis of control (CTR1-3), schizophrenia (SZ1-3), and autism (ASD1-3) groups. For proteome analysis, an intermediate time point between that used for transcriptomics at day 30 and that for calcium imaging (day 56) was chosen for two reasons: (1) the expected delayed response of alterations in the proteome as compared with the transcriptome favours a shift to later time points (2). Day 60 was suspected to be too late to detect dynamic changes at a time point when clones differing in developmental progress such as neurite outgrowth have reached a plateau phase. A total of 736 proteins were identified (at least one unique peptide in all nine samples) for which label free quantification data was obtained (Supplementary Table S3). Of the 26 jointly deregulated genes at the transcriptome level (Fig. 5b), none was identified with sufficient quantification data to compare transcript expression and protein abundance. Expression of vacuolar protein sorting-associated protein 35, was significantly downregulated by ~75% in the schizophrenia samples in comparison with the healthy control samples (*p* < 0.00001, FDR < 5%), while no significant difference was observed in the comparison of autism samples and healthy controls. This protein, which is involved in retrograde transport to the Golgi apparatus, has been found to be differentially expressed in postmortem cerebellar tissue of schizophrenia patients in a previous study^37^.

## Discussion

Our results suggest that examination of calcium signalling and analysis of transcriptome profiles may provide a mean to discriminate among healthy controls, schizophrenia, and autism samples in hiPSC-based models of different neurodevelopmental disorders. Nevertheless, we found considerable differences in the transcriptome of neurons derived from different individuals with schizophrenia in spite of selection of a well-characterized group of patients. Surprisingly, analysis of the transcriptome of one patient reveals deregulation of the MHC II antigen presentation pathway that is susceptible to clozapine treatment.

Neurodevelopmental disorders such as schizophrenia and autism share synaptic dysfunction as a major determinant and were proposed to be classified as “synaptopathies”^38^. More specifically, expression of PSD-95, a scaffolding protein of excitatory synapses, was shown to be downregulated in the dorsolateral and dorsomedial prefrontal cortex of patients with schizophrenia^39,40^ suggesting a link to hypofrontality. Likewise, a decrease in synaptic PSD95 clusters was observed with hiPSC-based models for SZ and ASD^9,10,41^, which is in accordance with our findings. Moreover, many proteins directly or indirectly interacting with PSD95 represent risk factors for idiopathic autism or underlie monogenic, ASD-associated syndromes like Phelan-McDermid syndrome^42–44^. Similar to reduced PSD95 cluster density, we found impaired neurite outgrowth in neuronal samples from patients with schizophrenia or autism independent of different medications applied, severity of SZ, and transcriptomic variability. Although consistent with published findings^11,45^, these assays did not inform on potential differences between these two neurodevelopmental diseases. By contrast, specific patterns of calcium signals discriminated healthy controls from schizophrenia and autism samples, and autism from schizophrenia samples. Thus, analysis of calcium signals appears to be a promising approach to discriminate among different diseases. hiPSC generation and differentiation may cause variability in phenotypic assays^46^. However, we processed a smaller number of samples within a short time period allowing for high reproducibility as confirmed by only small variability in functional assays and the proper segregation of healthy, SZ, and ASD samples after hierarchical clustering.

Haloperidol increased the percentage of CTR neurons with neurites, while neurite length was reduced at the example of the healthy control clone CTR1. This may be explained by different effects on the initiation versus the elongation of neurites. Reduced neuronal development or even neurotoxicity after haloperidol treatment was found in other reports using rodent neuronal cultures^47,48^. Overall, the experiments suggests that neurite outgrowth is not a suitable paradigm to test beneficial effects of antipsychotic drugs on iPSC-derived neurons prepared from SZ samples. However, inclusion of more iPSC clones is required for a final conclusion.

It was surprising to observe deregulation of mRNA expression profiles of many components of the MHC II antigen presentation pathway in hiPSC-derived neurons of patient SZ2 since MHC II-associated genes have so far not been described to be expressed in neurons. However, expression of MHC II-associated genes has been observed in human fetal neural precursor cells and in a subpopulation of human neural stem cells suggesting immaturity of the culture system^49,50^. Likewise, we cannot completely rule out the possibility that the differentiation protocol gives yield to a small population of microglial cells contributing to the detection of MHC class II transcripts in transcriptome analyses. The functional impact of MHC class II expression in neurons is still unclear. MHC II expressing neural stem and progenitor cells are potentially immunogenic and show MHC II upregulation upon IFNγ stimulation^50,51^. However, especially in the context of neuronal maturation, MHC II expression may also have important nonimmune implications as in the case of MHC I-related genes that were shown to be involved in synapse formation and functioning^52^. In this line, we found deregulated MHC class I-associated protein HLA-F and ERAP2 in patient SZ2 (Supplementary Table S2). The finding that clozapine treatment increases MHC class II expression in healthy control neurons raises the question whether clozapine treatment induces MHC class II expression in neurons. In this line, patient SZ2 received clozapine treatment, however, patient SZ3 also received clozapine without showing upregulation of MHC class II-associated genes. The divergent response may be explained either by different genetic backgrounds of the two patients or by the additional medication of SZ3 which may potentially blunt the clozapine effect.

In summary, we have shown that analysis of calcium signals is useful to discriminate autism and schizophrenia phenotypes in hiPSC-derived neurons derived from different patients. Likewise, we provided hints for a potential contribution of the MHC class II pathway to the phenotype of sample SZ2.

## Supplementary information

Supplemental table S2

Supplemental table S3

Supplemental table S1

Supplemental material
