# Supplementary material for: Comparative characterization of human induced pluripotent stem cells (hiPSC) derived from patients with schizophrenia and autism
Source: Transl Psychiatry. 2019 Jul 29;9:179. doi: 10.1038/s41398-019-0517-3 (PMC6663940; doi:10.1038/s41398-019-0517-3)
Supplement: Supplementary file 1 — Supplemental table S2 [file 41398_2019_517_MOESM1_ESM.pdf]

Supplemental table S2

Deregulated mRNAs of SZ2 compared to the group of healthy controls CTR1,2,3

gene_id	gene	baseMean	log2FoldChange	lfcSE	stat	pvalue	padj
ENSG00000224911	AC015936.3	93,3650197	4,244931072	1,02796543	4,12944923	3,64E-05	0,02193113
ENSG00000106819	ASPN	513,043841	4,082341174	0,89776295	4,5472373	5,44E-06	0,0047206
ENSG00000125730	C3	82,4096708	4,261980204	0,97676661	4,36335577	1,28E-05	0,00924111
ENSG00000151062	CACNA2D4	117,577691	4,06073088	0,95272836	4,26221267	2,02E-05	0,0137337
ENSG00000019582	CD74	12301,8847	5,961863689	0,93810421	6,35522539	2,08E-10	1,51E-06
ENSG00000164287	CDC20B	58,5358669	6,000599287	1,05161692	5,70606954	1,16E-08	2,28E-05
ENSG00000133048	CHI3L1	1454,28641	5,816924043	0,94003366	6,18799552	6,09E-10	2,21E-06
ENSG00000179583	CIITA	1020,07467	5,734069226	0,94506317	6,06739254	1,30E-09	4,03E-06
ENSG00000115844	DLX2	313,509902	4,151684406	0,94187496	4,4078934	1,04E-05	0,00838687
ENSG00000105880	DLX5	179,753483	4,16799157	0,94723063	4,40018665	1,08E-05	0,00838687
ENSG00000006377	DLX6	92,6039572	3,969684947	1,00097226	3,96582912	7,31E-05	0,03781054
ENSG00000231764	DLX6-AS1	1586,87752	5,081953235	0,89436801	5,68217242	1,33E-08	2,41E-05
ENSG00000070886	EPHA8	730,399894	-3,547127398	0,90424188	-3,92276387	8,75E-05	0,04357617
ENSG00000164308	ERAP2	773,564532	5,364702086	0,95254646	5,63195846	1,78E-08	2,76E-05
ENSG00000230316	FEZF1-AS1	33,8374737	4,417157088	1,07555311	4,10687026	4,01E-05	0,02353442
ENSG00000143631	FLG	20,4858799	4,600177245	1,09632116	4,19601245	2,72E-05	0,01734759
ENSG00000164694	FNDC1	273,356825	3,738919511	0,92488903	4,04256011	5,29E-05	0,03020868
ENSG00000134612	FOLH1B	38,4802338	5,585841019	1,079649	5,1737565	2,29E-07	0,00026218
ENSG00000176165	FOXG1	573,952944	4,1386971	1,06714164	3,87830158	0,00010519	0,04821119
ENSG00000117226	GBP3	401,835411	4,20877983	0,95245789	4,41886183	9,92E-06	0,0082858
ENSG00000131095	GFAP	48438,2639	3,824662228	0,91714363	4,17018893	3,04E-05	0,01887996
ENSG00000184674	GSTT1	911,695506	-4,780162341	0,91329899	-5,2339512	1,66E-07	0,00020014
ENSG00000206337	HCP5	196,747512	4,324725105	0,98521118	4,38964272	1,14E-05	0,0085004
ENSG00000204257	HLA-DMA	996,195529	4,218151482	0,90468679	4,66255452	3,12E-06	0,00282535
ENSG00000242574	HLA-DMB	344,812711	4,530533268	0,9039972	5,01166735	5,40E-07	0,00058579

ENSG00000204252	HLA-DOA	1295,47277	6,301866064	0,9358914	6,73354413	1,66E-11	1,80E-07
ENSG00000231389	HLA-DPA1	5359,91757	5,606313178	0,89132758	6,28984596	3,18E-10	1,56E-06
ENSG00000196735	HLA-DQA1	1511,60291	5,535999062	0,95550708	5,79378129	6,88E-09	1,49E-05
ENSG00000237541	HLA-DQA2	711,906614	5,210365051	1,05054945	4,95965709	7,06E-07	0,00073012
ENSG00000232629	HLA-DQB2	111,351614	6,029798144	1,0297285	5,85571649	4,75E-09	1,15E-05
ENSG00000204287	HLA-DRA	9921,96611	5,539264499	0,88332431	6,27092952	3,59E-10	1,56E-06
ENSG00000196126	HLA-DRB1	3829,96493	4,032116215	0,99930897	4,03490444	5,46E-05	0,03041045
ENSG00000198502	HLA-DRB5	930,565252	4,343492646	0,99693237	4,35685788	1,32E-05	0,00924111
ENSG00000229391	HLA-DRB6	433,344135	5,611980405	1,00680603	5,57404328	2,49E-08	3,60E-05
ENSG00000204642	HLA-F	272,374174	3,832808587	0,98919149	3,87468819	0,00010676	0,04821119
ENSG00000132204	LINC00470	66,8182205	4,729563678	0,99768982	4,7405151	2,13E-06	0,00210385
ENSG00000012223	LTF	1676,89576	6,802811782	0,99154255	6,860837	6,85E-12	1,49E-07
ENSG00000053438	NNAT	70431,1266	-4,702164387	0,88314132	-5,32436234	1,01E-07	0,00012939
ENSG00000111331	OAS3	498,562369	4,199238433	0,89146025	4,71051675	2,47E-06	0,00233252
ENSG00000160951	PTGER1	76,5995958	3,872015565	0,98759388	3,92065567	8,83E-05	0,04357617
ENSG00000198774	RASSF9	195,626248	3,727843295	0,9259836	4,02582	5,68E-05	0,03081862
ENSG00000250138	RP11-848G14.5	16,8870091	4,276938548	1,09745574	3,8971399	9,73E-05	0,04696325
ENSG00000177409	SAMD9L	286,832784	3,737104797	0,96176277	3,88568252	0,00010204	0,0481642
ENSG00000126778	SIX1	153,508514	3,771923811	0,97463952	3,87007067	0,0001088	0,04821119
ENSG00000226287	TMEM191A	177,126874	-3,729471994	0,93637446	-3,9828852	6,81E-05	0,03605444
ENSG00000150244	TRIM48	80,0229445	6,217231518	1,03887943	5,9845554	2,17E-09	5,89E-06
ENSG00000148704	VAX1	98,0218703	5,703404869	1,01246166	5,63320579	1,77E-08	2,76E-05
ENSG00000106018	VIPR2	240,506187	-4,161382975	0,98045961	-4,24431861	2,19E-05	0,01442589
ENSG00000197134	ZNF257	45,9548116	5,842963312	1,07331332	5,44385615	5,21E-08	7,08E-05
