# Supplementary material for: Comparative characterization of human induced pluripotent stem cells (hiPSC) derived from patients with schizophrenia and autism
Source: Transl Psychiatry. 2019 Jul 29;9:179. doi: 10.1038/s41398-019-0517-3 (PMC6663940; doi:10.1038/s41398-019-0517-3)
Supplement: Supplementary file 2 — Supplemental table S3 [file 41398_2019_517_MOESM2_ESM.pdf]

Supplemental table S3

Gene names	Protein names	Average (Healthy)	Average (Autism)	Average (SZ)	Fold Difference (Autism - Healthy)	Fold Difference (SZ - Healthy)	Fold Difference (SZ - Autism)	P-value Healthy vs Autism	P-value Healthy vs SZ	P-value Autism vs SZ	Q-value Healthy vs Autism	Q-value Healthy vs SZ	Q-value Autism vs SZ
**VPS35**	**Vacuolar protein sorting-associated protein 35**	**5,03**	**4,94**	**4,92**	**0,80**	**0,78**	**0,97**	**0,1148**	**0,0001**	**0,8055**	**0,2283**	**0,0234**	**0,6124**
CLTC	Clathrin heavy chain 1	6,05	5,88	5,87	0,67	0,66	0,99	0,0816	0,0003	0,9414	0,2248	0,0637	0,6561
SGTA	Small glutamine-rich tetratricopeptide repeat-containing protein alpha	4,17	4,41	4,28	1,71	1,27	0,74	0,0742	0,3450	0,0008	0,2248	0,4142	0,1841
PTPLAD1	Very-long-chain (3R)-3-hydroxyacyl-CoA dehydratase 3	4,80	4,71	4,49	0,80	0,48	0,60	0,4065	0,0009	0,1141	0,3450	0,1282	0,3354
RAB11B;RAB11A	Ras-related protein Rab-11B;Ras-related protein Rab-11A	4,76	4,94	4,63	1,50	0,74	0,49	0,0057	0,0091	0,0010	0,2248	0,1685	0,1841
ACTB	Actin, cytoplasmic 1;Actin, cytoplasmic 1, N-terminally processed	6,28	6,25	6,11	0,92	0,67	0,73	0,6843	0,1038	0,0017	0,4151	0,2759	0,1841
RPL8	60S ribosomal protein L8	3,67	3,76	3,96	1,23	1,96	1,60	0,1421	0,0020	0,0076	0,2397	0,1640	0,1841
SMU1	WD40 repeat-containing protein SMU1;WD40 repeat-containing protein	4,22	4,38	4,16	1,46	0,86	0,59	0,0092	0,0175	0,0022	0,2248	0,2036	0,1841
TUBB	Tubulin beta chain	6,21	6,24	6,02	1,07	0,64	0,60	0,6222	0,0381	0,0024	0,3979	0,2066	0,1841
MGEA5	Bifunctional protein NCOAT;Protein O-GlcNAcase;Histone acetyltransfer	4,30	4,49	4,29	1,57	0,97	0,62	0,0623	0,8912	0,0027	0,2248	0,5472	0,1841
RPL19	Ribosomal protein L19;60S ribosomal protein L19	3,99	4,15	4,29	1,44	1,97	1,36	0,1538	0,0028	0,1765	0,2451	0,1640	0,3720
RAB7A	Ras-related protein Rab-7a	5,02	5,24	5,06	1,65	1,10	0,67	0,0028	0,4916	0,0305	0,2248	0,4608	0,2831
RPS7	40S ribosomal protein S7	4,64	4,87	5,05	1,72	2,58	1,50	0,2064	0,0031	0,3319	0,2750	0,1640	0,4345
DCX	Neuronal migration protein doublecortin	5,11	5,22	4,64	1,29	0,34	0,27	0,5344	0,0462	0,0035	0,3789	0,2066	0,1841
NCAM1	Neural cell adhesion molecule 1	5,33	5,17	4,71	0,69	0,24	0,35	0,3498	0,0036	0,0477	0,3239	0,1640	0,2831
DCTN2	Dynactin subunit 2	4,75	4,94	4,48	1,54	0,54	0,35	0,2961	0,1811	0,0038	0,3116	0,3071	0,1841
H2AFY2	Core histone macro-H2A.2;Histone H2A	5,33	5,42	5,18	1,23	0,72	0,58	0,3637	0,1665	0,0043	0,3285	0,3027	0,1841
COPB1	Coatomer subunit beta	4,67	4,62	4,84	0,89	1,47	1,65	0,3778	0,0044	0,0101	0,3328	0,1640	0,1841
HSPB1	Heat shock protein beta-1	4,84	5,00	5,42	1,47	3,79	2,58	0,3372	0,0047	0,0748	0,3221	0,1640	0,2918
PPT1	Palmitoyl-protein thioesterase 1	4,12	4,14	4,19	1,05	1,18	1,12	0,6732	0,0047	0,3777	0,4111	0,1640	0,4438
OPA1	Dynamin-like 120 kDa protein, mitochondrial;Dynamin-like 120 kDa prot	4,55	4,50	4,18	0,90	0,43	0,47	0,7534	0,0594	0,0049	0,4326	0,2247	0,1841
PHB	Prohibitin	5,23	5,35	5,38	1,31	1,40	1,07	0,0051	0,0648	0,6325	0,2248	0,2293	0,5402
PDIA3	Protein disulfide-isomerase A3	5,69	5,57	5,78	0,76	1,22	1,62	0,0260	0,0128	0,0051	0,2248	0,1983	0,1841
VCL	Vinculin	4,95	4,51	5,20	0,36	1,75	4,84	0,0558	0,0054	0,0142	0,2248	0,1640	0,2177
PDIA6	Protein disulfide-isomerase A6	5,47	5,40	5,62	0,84	1,41	1,68	0,1942	0,0349	0,0054	0,2681	0,2036	0,1841
EIF5A	Eukaryotic translation initiation factor 5A-1	4,31	4,70	4,70	2,45	2,45	1,00	0,0552	0,0056	0,9963	0,2248	0,1640	0,6648
RCN1	Reticulocalbin-1	4,68	4,44	4,83	0,58	1,43	2,46	0,0474	0,1027	0,0059	0,2248	0,2753	0,1841
H3F3B;HIST2H3A;HIST	Histone H3;Histone H3.2;Histone H3.1t;Histone H3.3;Histone H3.1;Histo	4,23	4,80	4,65	3,74	2,67	0,71	0,0662	0,0061	0,5661	0,2248	0,1640	0,5074
RPL5	60S ribosomal protein L5	4,47	4,40	4,59	0,85	1,32	1,55	0,6184	0,4082	0,0062	0,3979	0,4344	0,1841
NIPSNAP3A	Protein NipSnap homolog 3A	4,12	4,22	4,40	1,26	1,88	1,49	0,5287	0,0066	0,3071	0,3778	0,1640	0,4247
RPS18	40S ribosomal protein S18	4,33	4,72	4,75	2,42	2,61	1,08	0,0570	0,0067	0,8432	0,2248	0,1640	0,6237
06. Sep	Septin-6	4,21	4,24	4,10	1,07	0,77	0,72	0,3947	0,0369	0,0068	0,3397	0,2036	0,1841
SOD2	Superoxide dismutase [Mn], mitochondrial	3,97	4,22	4,33	1,79	2,31	1,29	0,0170	0,0068	0,2309	0,2248	0,1640	0,3949
HSD17B12	Estradiol 17-beta-dehydrogenase 12	4,39	4,56	4,67	1,46	1,87	1,28	0,0211	0,0068	0,0939	0,2248	0,1640	0,3180
SNAP91	Clathrin coat assembly protein AP180	4,49	4,39	4,15	0,79	0,45	0,58	0,5857	0,0070	0,2335	0,3926	0,1640	0,3949
SERPINH1	Serpin H1	5,67	5,33	6,05	0,46	2,42	5,23	0,0248	0,0489	0,0071	0,2248	0,2147	0,1841
MACF1	Microtubule-actin cross-linking factor 1, isoforms 1/2/3/5	4,35	4,06	4,25	0,51	0,78	1,55	0,0071	0,8033	0,6548	0,2248	0,5317	0,5488
PPIB	Peptidyl-prolyl cis-trans isomerase B	5,13	5,30	5,54	1,49	2,60	1,75	0,0575	0,0074	0,0336	0,2248	0,1640	0,2831
SND1	Staphylococcal nuclease domain-containing protein 1	4,99	4,79	5,04	0,64	1,13	1,76	0,0182	0,1457	0,0074	0,2248	0,2911	0,1841
NADK2	NAD kinase 2, mitochondrial	3,87	3,64	3,81	0,59	0,87	1,47	0,0077	0,5282	0,1386	0,2248	0,4701	0,3520
PFN1	Profilin-1	4,91	5,30	5,21	2,44	2,01	0,82	0,0691	0,0079	0,6403	0,2248	0,1670	0,5450
FAM213A	Redox-regulatory protein FAM213A	4,37	4,57	4,55	1,58	1,51	0,95	0,0082	0,2857	0,8895	0,2248	0,3914	0,6445
MOGS	Mannosyl-oligosaccharide glucosidase	4,10	3,98	4,21	0,75	1,31	1,73	0,0445	0,0398	0,0082	0,2248	0,2066	0,1841
PKM	Pyruvate kinase PKM;Pyruvate kinase	4,37	4,49	4,14	1,31	0,59	0,45	0,3142	0,0426	0,0082	0,3185	0,2066	0,1841
CALU	Calumenin	4,83	4,38	4,92	0,36	1,23	3,45	0,0084	0,5339	0,0096	0,2248	0,4701	0,1841
PPP2R2A	Serine/threonine-protein phosphatase 2A 55 kDa regulatory subunit B a	4,28	4,32	4,22	1,10	0,88	0,80	0,7584	0,6912	0,0088	0,4335	0,5201	0,1841
NOLC1	Nucleolar and coiled-body phosphoprotein 1	3,05	3,21	3,33	1,44	1,89	1,31	0,0090	0,0447	0,2910	0,2248	0,2066	0,4247

ACO2	Aconitate hydratase, mitochondrial	5,42	5,31	5,31	0,77	0,77	1,00	0,2397	0,0092	0,9830	0,2853	0,1685	0,6619
PHB2	Prohibitin-2	5,23	5,28	5,38	1,12	1,40	1,26	0,0093	0,0620	0,1580	0,2248	0,2276	0,3626
DYNC1H1	Cytoplasmic dynein 1 heavy chain 1	6,08	5,97	5,91	0,77	0,68	0,88	0,1742	0,0093	0,4895	0,2560	0,1685	0,4752
NSFL1C	NSFL1 cofactor p47	4,25	4,47	4,14	1,64	0,77	0,47	0,0095	0,5111	0,1037	0,2248	0,4676	0,3299
KRT1	Keratin, type II cytoskeletal 1	5,19	4,24	4,57	0,11	0,24	2,13	0,0514	0,1484	0,0095	0,2248	0,2924	0,1841
PDE6H;MYL6	Myosin light polypeptide 6	4,20	4,65	4,57	2,85	2,33	0,82	0,1428	0,0095	0,7464	0,2397	0,1685	0,5860
PGAM1	Phosphoglycerate mutase 1	4,88	5,36	5,15	2,98	1,85	0,62	0,0098	0,1640	0,2702	0,2248	0,3027	0,4160
TLN1	Talin-1	4,70	4,24	5,04	0,35	2,18	6,20	0,0790	0,0690	0,0099	0,2248	0,2321	0,1841
P4HB	Protein disulfide-isomerase	5,49	5,37	5,70	0,75	1,64	2,18	0,0716	0,0226	0,0099	0,2248	0,2036	0,1841
TUBB4B	Tubulin beta-4B chain	6,02	6,06	5,80	1,08	0,61	0,56	0,5423	0,0278	0,0099	0,3801	0,2036	0,1841
SEC13	Protein SEC13 homolog	3,62	3,72	3,90	1,27	1,89	1,49	0,3942	0,0603	0,0105	0,3397	0,2259	0,1848
CALD1	Caldesmon	4,43	3,90	4,35	0,30	0,84	2,84	0,0776	0,7610	0,0111	0,2248	0,5288	0,1884
RTN4	Reticulon-4	5,36	5,22	5,18	0,72	0,66	0,92	0,1466	0,0113	0,6584	0,2425	0,1907	0,5508
LASP1	LIM and SH3 domain protein 1	4,43	4,61	4,51	1,54	1,23	0,79	0,1090	0,3816	0,0117	0,2265	0,4226	0,1912
PGK1	Phosphoglycerate kinase 1	5,62	5,99	5,76	2,34	1,37	0,58	0,0121	0,3711	0,1589	0,2248	0,4203	0,3626
YWHAZ	14-3-3 protein zeta/delta	5,59	5,84	5,62	1,77	1,05	0,59	0,0126	0,8797	0,1608	0,2248	0,5472	0,3626
ATP2B4	Plasma membrane calcium-transporting ATPase 4	4,62	4,24	4,14	0,42	0,34	0,80	0,0289	0,0126	0,5350	0,2248	0,1983	0,4934
ATP5H	ATP synthase subunit d, mitochondrial	4,06	4,36	4,52	1,99	2,85	1,43	0,0572	0,0131	0,0297	0,2248	0,1983	0,2831
HSPA6;HSPA7	Heat shock 70 kDa protein 6;Putative heat shock 70 kDa protein 7	3,55	3,72	3,85	1,50	2,00	1,33	0,1907	0,0540	0,0142	0,2673	0,2147	0,2177
AP3D1	AP-3 complex subunit delta-1	4,34	4,28	4,20	0,87	0,73	0,84	0,2613	0,0143	0,2334	0,2951	0,2036	0,3949
HSP90B1	Endoplasmin	5,89	5,75	6,02	0,72	1,36	1,88	0,1018	0,0640	0,0156	0,2253	0,2293	0,2281
PGRMC1	Membrane-associated progesterone receptor component 1	4,61	4,77	4,60	1,43	0,98	0,68	0,1043	0,9020	0,0158	0,2253	0,5472	0,2281
RPL7	60S ribosomal protein L7	4,64	4,72	4,89	1,18	1,77	1,49	0,3097	0,0159	0,0502	0,3185	0,2036	0,2831
ATP5F1	ATP synthase F(0) complex subunit B1, mitochondrial	4,43	4,51	4,50	1,22	1,19	0,98	0,0160	0,4572	0,9232	0,2248	0,4491	0,6542
CTNNB1	Catenin beta-1	4,91	4,70	4,63	0,61	0,53	0,86	0,3113	0,0164	0,7452	0,3185	0,2036	0,5860
FBL	rRNA 2-O-methyltransferase fibrillarin	4,46	4,52	4,71	1,16	1,77	1,53	0,4193	0,0166	0,0235	0,3481	0,2036	0,2622
C21orf33	ES1 protein homolog, mitochondrial	3,76	3,96	3,96	1,60	1,58	0,99	0,0167	0,2074	0,9711	0,2248	0,3352	0,6605
PPP2R1A	Serine/threonine-protein phosphatase 2A 65 kDa regulatory subunit A a	5,50	5,51	5,34	1,03	0,70	0,68	0,8114	0,0300	0,0178	0,4477	0,2036	0,2474
HK1	Hexokinase-1	5,31	5,05	4,81	0,55	0,32	0,58	0,1296	0,0179	0,1462	0,2363	0,2036	0,3554
RAB1A	Ras-related protein Rab-1A	4,84	5,09	5,02	1,79	1,50	0,84	0,0218	0,0179	0,2951	0,2248	0,2036	0,4247
RPL15	Ribosomal protein L15;60S ribosomal protein L15	4,14	4,27	4,47	1,35	2,14	1,59	0,1348	0,0179	0,1120	0,2397	0,2036	0,3354
ATP5O	ATP synthase subunit O, mitochondrial	4,65	4,86	4,87	1,62	1,65	1,02	0,0180	0,1529	0,9519	0,2248	0,2943	0,6568
LMNA	Prelamin-A/C;Lamin-A/C	5,01	4,39	5,64	0,24	4,24	17,81	0,1804	0,0523	0,0181	0,2587	0,2147	0,2474
YWHAG	14-3-3 protein gamma;14-3-3 protein gamma, N-terminally processed	5,04	5,30	4,92	1,82	0,76	0,42	0,0182	0,5174	0,0750	0,2248	0,4680	0,2918
PSMD8	26S proteasome non-ATPase regulatory subunit 8	4,22	4,44	4,39	1,66	1,50	0,91	0,0183	0,0343	0,3177	0,2248	0,2036	0,4294
RPL24	60S ribosomal protein L24	4,39	4,69	4,77	1,96	2,40	1,22	0,2205	0,0188	0,6602	0,2791	0,2036	0,5515
RPS13	40S ribosomal protein S13	4,56	4,83	4,83	1,89	1,87	0,99	0,1135	0,0188	0,9813	0,2283	0,2036	0,6619
PDIA3		4,62	4,40	4,65	0,61	1,08	1,75	0,0979	0,7075	0,0195	0,2248	0,5201	0,2566
MLEC	Malectin	4,07	4,01	4,23	0,87	1,45	1,68	0,4238	0,0328	0,0199	0,3492	0,2036	0,2566
YBX1	Nuclease-sensitive element-binding protein 1	4,81	4,52	4,38	0,52	0,37	0,72	0,0202	0,0363	0,3553	0,2248	0,2036	0,4375
PRDX4	Peroxiredoxin-4	4,41	4,63	4,81	1,67	2,51	1,50	0,0980	0,0202	0,1349	0,2248	0,2036	0,3500
RAN	GTP-binding nuclear protein Ran	5,09	5,30	5,27	1,62	1,51	0,93	0,0446	0,0216	0,6856	0,2248	0,2036	0,5659
ARF1;ARF3	ADP-ribosylation factor 1;ADP-ribosylation factor 3	5,06	5,34	5,28	1,91	1,64	0,86	0,0216	0,1277	0,5865	0,2248	0,2810	0,5181
MATR3	Matrin-3	5,62	5,59	5,52	0,94	0,79	0,84	0,7327	0,2376	0,0222	0,4291	0,3633	0,2622
TUBB4A	Tubulin beta-4A chain	5,32	5,24	4,90	0,84	0,38	0,45	0,6271	0,0223	0,0468	0,3982	0,2036	0,2831
SF3B3	Splicing factor 3B subunit 3	4,54	4,56	4,67	1,05	1,34	1,28	0,9234	0,5424	0,0224	0,4771	0,4701	0,2622
TMED9	Transmembrane emp24 domain-containing protein 9	4,14	4,24	4,37	1,26	1,72	1,37	0,0697	0,0226	0,0686	0,2248	0,2036	0,2918
RAB6B	Ras-related protein Rab-6B	4,86	5,05	4,86	1,53	0,99	0,64	0,0230	0,8972	0,0302	0,2248	0,5472	0,2831
HSDL1	Inactive hydroxysteroid dehydrogenase-like protein 1	4,33	4,18	3,66	0,71	0,21	0,30	0,5584	0,0230	0,0407	0,3838	0,2036	0,2831
SNRPD3	Small nuclear ribonucleoprotein Sm D3	4,07	4,53	4,34	2,91	1,87	0,64	0,0628	0,0233	0,3360	0,2248	0,2036	0,4350
HADHB	Trifunctional enzyme subunit beta, mitochondrial;3-ketoacyl-CoA thiolas	4,62	4,56	4,77	0,87	1,42	1,63	0,3524	0,1421	0,0236	0,3239	0,2908	0,2622
MAPRE1	Microtubule-associated protein RP/EB family member 1	4,69	4,70	4,41	1,02	0,52	0,51	0,9431	0,1390	0,0238	0,4825	0,2881	0,2622
RPS4X	40S ribosomal protein S4, X isoform	4,73	4,76	4,90	1,08	1,48	1,37	0,5790	0,0331	0,0238	0,3926	0,2036	0,2622
LSS	Lanosterol synthase	4,58	4,31	4,31	0,53	0,53	1,01	0,1070	0,0239	0,9857	0,2262	0,2036	0,6619
FARSB	Phenylalanine--tRNA ligase beta subunit	4,37	4,45	4,35	1,22	0,97	0,80	0,2421	0,8479	0,0240	0,2853	0,5393	0,2622
RPL3	60S ribosomal protein L3	4,79	4,72	4,89	0,84	1,26	1,50	0,4992	0,4178	0,0246	0,3696	0,4365	0,2629

ACTN4	Alpha-actinin-4	5,51	5,29	5,52	0,60	1,01	1,70	0,0733	0,9440	0,0258	0,2248	0,5592	0,2691
RPL26;KRBA2;RPL26L1	60S ribosomal protein L26-like 1;60S ribosomal protein L26	3,90	3,99	4,14	1,21	1,74	1,44	0,4697	0,0261	0,1262	0,3631	0,2036	0,3411
SRRT	Serrate RNA effector molecule homolog	3,77	3,46	3,48	0,49	0,51	1,04	0,0280	0,0266	0,7062	0,2248	0,2036	0,5714
DDX17	Probable ATP-dependent RNA helicase DDX17	5,51	5,51	5,45	0,98	0,86	0,88	0,9193	0,4330	0,0272	0,4757	0,4405	0,2762
CAPNS1	Calpain small subunit 1	4,16	4,36	4,54	1,58	2,38	1,51	0,0896	0,0273	0,2202	0,2248	0,2036	0,3949
RAB10	Ras-related protein Rab-10	4,49	4,60	4,59	1,29	1,25	0,96	0,0300	0,0273	0,7235	0,2248	0,2036	0,5787
RPL17	60S ribosomal protein L17	4,52	4,63	4,73	1,27	1,60	1,26	0,3514	0,0273	0,3634	0,3239	0,2036	0,4375
VPS4B	Vacuolar protein sorting-associated protein 4B	3,86	3,82	3,99	0,90	1,34	1,48	0,5995	0,0275	0,0811	0,3926	0,2036	0,2929
HDLBP	Vigilin	4,31	4,09	4,52	0,59	1,61	2,72	0,0904	0,1712	0,0276	0,2248	0,3057	0,2762
GSTP1	Glutathione S-transferase P	5,28	5,65	5,59	2,36	2,07	0,88	0,0420	0,0278	0,6709	0,2248	0,2036	0,5566
RPL10;RPL10L	60S ribosomal protein L10;60S ribosomal protein L10-like	4,25	4,47	4,63	1,68	2,40	1,43	0,0544	0,0280	0,2611	0,2248	0,2036	0,4124
GNB2L1	Guanine nucleotide-binding protein subunit beta-2-like 1;Guanine nucle	4,92	4,97	5,14	1,12	1,65	1,48	0,5196	0,0287	0,0994	0,3756	0,2036	0,3265
CTNND1	Catenin delta-1	4,42	4,41	4,32	0,97	0,80	0,83	0,7362	0,0294	0,0751	0,4291	0,2036	0,2918
ATP5J2-PTCD1;ATP5J2	ATP synthase subunit f, mitochondrial	4,01	4,20	4,11	1,55	1,25	0,80	0,0302	0,4601	0,4474	0,2248	0,4491	0,4665
SARS	Serine--tRNA ligase, cytoplasmic	4,75	4,88	4,81	1,35	1,14	0,84	0,0306	0,1797	0,2113	0,2248	0,3070	0,3949
11. Sep	Septin-11	4,98	4,98	4,86	1,00	0,76	0,76	0,9518	0,0307	0,0466	0,4843	0,2036	0,2831
RPN2	Dolichyl-diphosphooligosaccharide--protein glycosyltransferase subunit 2	5,10	4,87	5,02	0,59	0,83	1,40	0,0308	0,5478	0,3257	0,2248	0,4701	0,4335
HSPD1	60 kDa heat shock protein, mitochondrial	6,01	5,89	5,99	0,76	0,96	1,25	0,0312	0,6761	0,0858	0,2248	0,5151	0,3018
RPL21	60S ribosomal protein L21	4,39	4,51	4,69	1,33	2,00	1,50	0,3031	0,0313	0,0783	0,3158	0,2036	0,2918
RPL4	60S ribosomal protein L4	5,05	5,07	5,24	1,06	1,54	1,45	0,8793	0,2953	0,0313	0,4682	0,3972	0,2831
GPI	Glucose-6-phosphate isomerase	5,29	5,58	5,33	1,96	1,11	0,56	0,0321	0,8000	0,1652	0,2248	0,5305	0,3639
PHGDH	D-3-phosphoglycerate dehydrogenase	5,10	5,29	5,41	1,56	2,03	1,30	0,1435	0,0325	0,1251	0,2397	0,2036	0,3411
HIST2H3PS2	Histone H3	4,74	5,20	4,98	2,90	1,77	0,61	0,0752	0,0326	0,3113	0,2248	0,2036	0,4258
EIF3E	Eukaryotic translation initiation factor 3 subunit E	4,40	4,38	4,51	0,94	1,28	1,36	0,7876	0,2783	0,0327	0,4406	0,3891	0,2831
TOP2B	DNA topoisomerase 2-beta;DNA topoisomerase 2	5,00	5,02	4,89	1,05	0,78	0,74	0,8504	0,3708	0,0327	0,4582	0,4203	0,2831
KLC1	Kinesin light chain 1	4,83	4,82	4,64	0,99	0,64	0,65	0,9607	0,0611	0,0328	0,4881	0,2269	0,2831
PDIA4	Protein disulfide-isomerase A4	5,18	5,07	5,29	0,77	1,27	1,65	0,2077	0,0329	0,0405	0,2753	0,2036	0,2831
NAGK	N-acetyl-D-glucosamine kinase	4,14	4,30	4,16	1,45	1,05	0,72	0,1593	0,8248	0,0332	0,2480	0,5351	0,2831
MDH2	Malate dehydrogenase, mitochondrial;Malate dehydrogenase	5,44	5,54	5,53	1,25	1,24	1,00	0,0336	0,1937	0,9758	0,2248	0,3206	0,6619
RPS23	40S ribosomal protein S23	4,09	4,45	4,37	2,30	1,92	0,84	0,0601	0,0339	0,6529	0,2248	0,2036	0,5488
FASN	Fatty acid synthase;[Acyl-carrier-protein] S-acetyltransferase;[Acyl-carrie	5,67	5,50	5,57	0,67	0,79	1,18	0,0341	0,0986	0,3043	0,2248	0,2702	0,4247
FLOT1	Flotillin-1	4,28	4,45	4,52	1,49	1,75	1,17	0,1677	0,0341	0,3707	0,2538	0,2036	0,4438
ECH1	Delta(3,5)-Delta(2,4)-dienoyl-CoA isomerase, mitochondrial	4,37	4,68	4,74	2,06	2,38	1,16	0,0900	0,0341	0,5487	0,2248	0,2036	0,4987
IPO7	Importin-7	4,70	4,61	4,62	0,82	0,84	1,02	0,0342	0,4294	0,9427	0,2248	0,4405	0,6561
YWHAE	14-3-3 protein epsilon	5,82	6,08	5,86	1,82	1,09	0,60	0,0349	0,8504	0,2463	0,2248	0,5401	0,3987
ATP1A3	Sodium/potassium-transporting ATPase subunit alpha-3	5,60	5,27	5,00	0,46	0,25	0,55	0,1794	0,0353	0,3564	0,2587	0,2036	0,4375
LRRC59	Leucine-rich repeat-containing protein 59	4,52	4,53	4,75	1,01	1,68	1,67	0,9474	0,0354	0,0426	0,4834	0,2036	0,2831
STOML2	Stomatin-like protein 2, mitochondrial	4,40	4,33	4,50	0,86	1,27	1,48	0,1700	0,1242	0,0361	0,2538	0,2810	0,2831
NCKAP1	Nck-associated protein 1	4,58	4,46	4,37	0,76	0,62	0,81	0,1526	0,0361	0,3768	0,2451	0,2036	0,4438
GOT2	Aspartate aminotransferase, mitochondrial	5,11	5,12	4,96	1,04	0,71	0,68	0,7123	0,0798	0,0369	0,4251	0,2497	0,2831
LDHA	L-lactate dehydrogenase A chain	4,80	5,14	5,51	2,15	5,10	2,38	0,2068	0,0370	0,2131	0,2750	0,2036	0,3949
PGM1	Phosphoglucomutase-1	3,71	4,09	4,49	2,39	5,95	2,48	0,2220	0,0370	0,1327	0,2791	0,2036	0,3491
PPP1R7	Protein phosphatase 1 regulatory subunit 7	4,40	4,55	4,39	1,44	0,98	0,68	0,0447	0,9184	0,0372	0,2248	0,5542	0,2831
PRDX3	Thioredoxin-dependent peroxide reductase, mitochondrial	4,64	4,86	4,79	1,68	1,41	0,84	0,0381	0,1394	0,2207	0,2248	0,2881	0,3949
PRDX5	Peroxiredoxin-5, mitochondrial	4,79	5,15	4,86	2,30	1,19	0,51	0,0381	0,5959	0,1339	0,2248	0,4911	0,3499
SNRPD1	Small nuclear ribonucleoprotein Sm D1	4,24	4,83	4,60	3,90	2,30	0,59	0,0518	0,0386	0,3325	0,2248	0,2066	0,4345
RAB2A	Ras-related protein Rab-2A	4,76	5,09	4,80	2,13	1,08	0,51	0,0387	0,8689	0,1329	0,2248	0,5453	0,3491
IMMT	MICOS complex subunit MIC60	4,91	4,78	4,85	0,73	0,87	1,18	0,0392	0,4006	0,3421	0,2248	0,4318	0,4358
HIST1H2BN;HIST1H2BI	Histone H2B;Histone H2B type 1-M;Histone H2B type 1-N;Histone H2B ty	6,08	6,43	6,26	2,26	1,51	0,67	0,1280	0,0393	0,3863	0,2363	0,2066	0,4454
CYB5R3	NADH-cytochrome b5 reductase 3;NADH-cytochrome b5 reductase 3 me	4,95	5,12	5,02	1,46	1,18	0,81	0,1263	0,4332	0,0394	0,2357	0,4405	0,2831
TPI1	Triosephosphate isomerase	5,16	5,78	5,54	4,22	2,41	0,57	0,0397	0,1647	0,3244	0,2248	0,3027	0,4329
CALR	Calreticulin	5,55	5,43	5,64	0,77	1,24	1,61	0,1730	0,1112	0,0400	0,2560	0,2771	0,2831
CEP170	Centrosomal protein of 170 kDa	4,28	4,27	3,92	0,98	0,44	0,45	0,8898	0,0532	0,0402	0,4703	0,2147	0,2831
CKAP5	Cytoskeleton-associated protein 5	4,59	4,53	4,14	0,88	0,36	0,41	0,6220	0,0403	0,0526	0,3979	0,2066	0,2833
PRKDC	DNA-dependent protein kinase catalytic subunit	5,49	5,37	5,53	0,76	1,09	1,44	0,1840	0,6423	0,0406	0,2598	0,5066	0,2831
YWHAQ	14-3-3 protein theta	5,27	5,45	5,17	1,48	0,79	0,53	0,0406	0,5334	0,1354	0,2248	0,4701	0,3500
MAP4	Microtubule-associated protein;Microtubule-associated protein 4	4,76	4,58	4,66	0,67	0,81	1,20	0,0411	0,4601	0,4908	0,2248	0,4491	0,4752

CSNK2A1;CSNK2A3	Casein kinase II subunit alpha;Casein kinase II subunit alpha 3	4,49	4,65	4,46	1,45	0,94	0,64	0,1169	0,7826	0,0411	0,2299	0,5305	0,2831
GNAS	Guanine nucleotide-binding protein G(s) subunit alpha isoforms short;Gu	5,19	5,28	5,04	1,25	0,72	0,58	0,1624	0,1495	0,0413	0,2506	0,2932	0,2831
DYNC1LI1	Cytoplasmic dynein 1 light intermediate chain 1	4,44	4,48	4,24	1,11	0,63	0,57	0,7423	0,2619	0,0414	0,4295	0,3799	0,2831
HSPA1A	Heat shock 70 kDa protein 1A/1B	5,27	5,35	5,28	1,20	1,04	0,86	0,0415	0,8760	0,5104	0,2248	0,5472	0,4794
H2AFY	Core histone macro-H2A.1;Histone H2A	5,25	5,29	5,08	1,08	0,68	0,63	0,5333	0,0564	0,0423	0,3789	0,2189	0,2831
EIF3C;EIF3CL	Eukaryotic translation initiation factor 3 subunit C;Eukaryotic translation	4,29	4,06	4,19	0,60	0,80	1,33	0,1143	0,4288	0,0424	0,2283	0,4405	0,2831
DNM1	Dynamin-1	4,64	4,51	4,13	0,74	0,31	0,42	0,5628	0,0430	0,0601	0,3841	0,2066	0,2918
RPL18	60S ribosomal protein L18	4,52	4,82	4,97	1,97	2,79	1,42	0,1428	0,0435	0,3366	0,2397	0,2066	0,4350
TMED10	Transmembrane emp24 domain-containing protein 10	4,47	4,50	4,69	1,08	1,68	1,55	0,7785	0,0435	0,2207	0,4393	0,2066	0,3949
ITGB1	Integrin beta-1	4,23	4,10	4,55	0,73	2,05	2,79	0,5915	0,3043	0,0438	0,3926	0,4054	0,2831
TMED2	Transmembrane emp24 domain-containing protein 2	4,16	4,47	4,51	2,05	2,25	1,10	0,0440	0,0448	0,7853	0,2248	0,2066	0,6027
RPS3	40S ribosomal protein S3	5,23	5,28	5,40	1,13	1,50	1,33	0,3594	0,0441	0,0596	0,3262	0,2066	0,2918
PAK2	Serine/threonine-protein kinase PAK 2;PAK-2p27;PAK-2p34	4,23	4,42	3,99	1,54	0,57	0,37	0,3156	0,0574	0,0443	0,3185	0,2189	0,2831
PRDX1	Peroxiredoxin-1	5,15	5,44	5,42	1,94	1,86	0,96	0,0864	0,0444	0,8939	0,2248	0,2066	0,6445
FH	Fumarate hydratase, mitochondrial	4,90	4,74	4,75	0,68	0,70	1,03	0,0446	0,2612	0,9291	0,2248	0,3799	0,6545
IMPDH2	Inosine-5-monophosphate dehydrogenase 2	4,49	4,54	4,71	1,13	1,68	1,49	0,7405	0,0453	0,2603	0,4293	0,2066	0,4124
ANXA5	Annexin A5;Annexin	5,56	5,56	5,88	1,00	2,07	2,07	0,9993	0,0454	0,0490	0,4969	0,2066	0,2831
DDX3X	ATP-dependent RNA helicase DDX3X	4,91	4,82	4,86	0,81	0,89	1,10	0,0456	0,6335	0,6919	0,2248	0,5035	0,5682
RPS3A	40S ribosomal protein S3a	4,52	4,58	4,70	1,15	1,50	1,31	0,4093	0,0459	0,1264	0,3458	0,2066	0,3411
RALA	Ras-related protein Ral-A	4,36	4,53	4,42	1,47	1,13	0,77	0,0460	0,5384	0,1255	0,2248	0,4701	0,3411
AP2B1	AP-2 complex subunit beta	5,19	5,11	5,02	0,83	0,68	0,81	0,3030	0,0463	0,3327	0,3158	0,2066	0,4345
RAC1;RAC2	Ras-related C3 botulinum toxin substrate 1;Ras-related C3 botulinum tox	4,34	4,57	4,49	1,69	1,39	0,82	0,0467	0,1342	0,3916	0,2248	0,2815	0,4472
TSN	Translin	4,17	4,68	4,48	3,23	2,00	0,62	0,0468	0,2037	0,3610	0,2248	0,3318	0,4375
PRDX2	Peroxiredoxin-2	4,92	5,45	5,02	3,42	1,24	0,36	0,0470	0,6245	0,1041	0,2248	0,5017	0,3299
CKAP4	Cytoskeleton-associated protein 4	5,27	4,96	5,35	0,49	1,21	2,48	0,0519	0,5673	0,0472	0,2248	0,4749	0,2831
UQCRC2	Cytochrome b-c1 complex subunit 2, mitochondrial	4,95	4,76	4,80	0,65	0,72	1,10	0,0473	0,1194	0,5044	0,2248	0,2810	0,4794
ARPC4-TTLL3;ARPC4	Actin-related protein 2/3 complex subunit 4	4,49	4,80	4,70	2,03	1,62	0,80	0,0478	0,0885	0,4993	0,2248	0,2550	0,4781
INA	Alpha-internexin	5,93	5,42	4,36	0,31	0,03	0,09	0,0478	0,1429	0,2913	0,2248	0,2908	0,4247
DDX6	Probable ATP-dependent RNA helicase DDX6	4,81	4,84	4,70	1,07	0,77	0,72	0,4804	0,1549	0,0483	0,3631	0,2956	0,2831
SLC25A1	Tricarboxylate transport protein, mitochondrial	4,45	4,62	4,49	1,46	1,09	0,75	0,0484	0,7987	0,3604	0,2248	0,5305	0,4375
NIPSNAP1	Protein NipSnap homolog 1	4,52	4,80	4,44	1,94	0,84	0,43	0,0484	0,4892	0,0529	0,2248	0,4608	0,2833
ACTR1A	Alpha-centractin	5,08	5,17	4,92	1,22	0,68	0,56	0,1230	0,1238	0,0485	0,2351	0,2810	0,2831
GNB1	Guanine nucleotide-binding protein G(I)/G(S)/G(T) subunit beta-1	4,74	4,84	4,46	1,25	0,52	0,42	0,0488	0,1199	0,0531	0,2248	0,2810	0,2833
TUBB2A	Tubulin beta-2A chain	6,87	6,88	6,59	1,02	0,52	0,51	0,9084	0,0683	0,0489	0,4741	0,2315	0,2831
09. Sep	Septin-9	4,59	4,81	4,89	1,66	2,00	1,20	0,0492	0,0664	0,4913	0,2248	0,2293	0,4752
TMEM43	Transmembrane protein 43	4,35	4,28	4,59	0,85	1,73	2,03	0,3929	0,1079	0,0493	0,3397	0,2771	0,2831
TUBB3	Tubulin beta-3 chain	6,18	6,23	5,65	1,12	0,30	0,26	0,6508	0,0814	0,0496	0,4056	0,2497	0,2831
EEF2	Elongation factor 2	5,66	5,60	5,74	0,87	1,21	1,39	0,0756	0,2090	0,0498	0,2248	0,3353	0,2831
GNAI2	Guanine nucleotide-binding protein G(i) subunit alpha-2	5,25	5,31	5,00	1,14	0,56	0,49	0,0501	0,3363	0,2495	0,2248	0,4142	0,4018
NOP58	Nucleolar protein 58	4,23	4,37	4,58	1,36	2,20	1,61	0,1972	0,0513	0,1698	0,2688	0,2147	0,3685
NDUFS1	NADH-ubiquinone oxidoreductase 75 kDa subunit, mitochondrial	4,66	4,39	4,30	0,54	0,44	0,81	0,2940	0,0516	0,6886	0,3115	0,2147	0,5674
RPS8	40S ribosomal protein S8	4,86	5,05	5,21	1,54	2,24	1,46	0,2252	0,0518	0,1190	0,2811	0,2147	0,3392
CKB	Creatine kinase B-type	5,99	6,28	5,93	1,98	0,88	0,45	0,0758	0,6597	0,0521	0,2248	0,5107	0,2833
DHX15	Putative pre-mRNA-splicing factor ATP-dependent RNA helicase DHX15	4,79	4,88	4,88	1,22	1,21	0,99	0,1593	0,0523	0,9445	0,2480	0,2147	0,6561
07. Sep	Septin-7	5,06	5,08	5,00	1,04	0,88	0,84	0,4705	0,0718	0,0524	0,3631	0,2356	0,2833
XPO1	Exportin-1	4,97	4,82	4,96	0,70	0,96	1,38	0,0529	0,7871	0,1222	0,2248	0,5305	0,3392
PRDX6	Peroxiredoxin-6	5,04	5,21	5,46	1,48	2,60	1,75	0,1286	0,0534	0,2208	0,2363	0,2147	0,3949
MAP4K4;MINK1	Mitogen-activated protein kinase kinase kinase kinase 4;Misshapen-like	4,16	4,04	3,96	0,75	0,62	0,83	0,4760	0,0535	0,6422	0,3631	0,2147	0,5457
AK1	Adenylate kinase isoenzyme 1	4,44	4,94	5,08	3,21	4,36	1,36	0,0933	0,0536	0,1212	0,2248	0,2147	0,3392
DLAT	Acetyltransferase component of pyruvate dehydrogenase complex;Dihyd	4,47	4,30	4,37	0,67	0,79	1,17	0,0537	0,3548	0,5195	0,2248	0,4175	0,4860
PAFAH1B3	Platelet-activating factor acetylhydrolase IB subunit gamma	4,82	5,20	4,13	2,44	0,21	0,08	0,0671	0,1594	0,0542	0,2248	0,2982	0,2848
THY1	Thy-1 membrane glycoprotein	4,16	3,97	4,52	0,65	2,29	3,52	0,4860	0,0542	0,0785	0,3631	0,2147	0,2918
PPIA	Peptidyl-prolyl cis-trans isomerase A;Peptidyl-prolyl cis-trans isomerase	5,39	5,78	5,59	2,51	1,59	0,64	0,0543	0,1302	0,2961	0,2248	0,2810	0,4247
CRKL	Crk-like protein	4,37	4,63	4,39	1,82	1,04	0,57	0,1234	0,8633	0,0546	0,2351	0,5442	0,2848
PFKL	ATP-dependent 6-phosphofructokinase, liver type	4,93	5,14	5,01	1,60	1,18	0,74	0,0551	0,5424	0,1731	0,2248	0,4701	0,3691
ACTN1	Alpha-actinin-1	5,14	4,12	4,97	0,09	0,68	7,18	0,0824	0,6724	0,0551	0,2248	0,5150	0,2848
VAT1	Synaptic vesicle membrane protein VAT-1 homolog	5,34	5,17	5,03	0,67	0,49	0,74	0,1497	0,0552	0,3312	0,2432	0,2166	0,4345

PSMA5	Proteasome subunit alpha type-5	4,59	4,90	4,85	2,03	1,81	0,89	0,0563	0,0661	0,6319	0,2248	0,2293	0,5402
PLEKHH3	Pleckstrin homology domain-containing family H member 3	4,75	4,83	4,42	1,20	0,47	0,39	0,3073	0,1042	0,0566	0,3184	0,2759	0,2895
CIRBP	Cold-inducible RNA-binding protein	4,44	4,85	4,68	2,62	1,76	0,67	0,0568	0,2182	0,2085	0,2248	0,3475	0,3949
NSF	Vesicle-fusing ATPase	4,99	4,84	4,83	0,71	0,69	0,98	0,2626	0,0573	0,9440	0,2956	0,2189	0,6561
FUBP3	Far upstream element-binding protein 3	4,05	4,10	4,30	1,14	1,79	1,58	0,7231	0,1663	0,0575	0,4289	0,3027	0,2909
ELAVL1	ELAV-like protein 1	4,85	4,90	4,83	1,13	0,96	0,85	0,2744	0,7060	0,0587	0,3042	0,5201	0,2918
CFL1	Cofilin-1	5,49	5,78	5,60	1,93	1,27	0,65	0,0593	0,2918	0,1588	0,2248	0,3962	0,3626
SUGP2	SURP and G-patch domain-containing protein 2	3,79	3,67	3,80	0,76	1,04	1,36	0,0597	0,8716	0,2372	0,2248	0,5453	0,3949
CORO1C	Coronin;Coronin-1C	4,74	4,81	4,53	1,19	0,62	0,52	0,3524	0,0945	0,0612	0,3239	0,2643	0,2918
GLO1	Lactoylglutathione lyase	4,34	4,54	4,59	1,57	1,79	1,14	0,2406	0,0623	0,7167	0,2853	0,2276	0,5751
MYH10	Myosin-10	5,63	5,50	5,61	0,75	0,95	1,26	0,0628	0,7499	0,1801	0,2248	0,5268	0,3754
HNRNPH3	Heterogeneous nuclear ribonucleoprotein H3	4,97	5,18	5,01	1,61	1,09	0,68	0,1312	0,7282	0,0632	0,2376	0,5245	0,2918
NUDT11;NUDT10	Diphosphoinositol polyphosphate phosphohydrolase 3-beta;Diphosphoir	3,19	3,41	3,16	1,67	0,95	0,57	0,0639	0,8138	0,1019	0,2248	0,5333	0,3270
PAFAH1B2	Platelet-activating factor acetylhydrolase IB subunit beta	4,44	4,77	4,59	2,13	1,42	0,67	0,0640	0,3272	0,1304	0,2248	0,4137	0,3491
ARPC1A	Actin-related protein 2/3 complex subunit 1A	4,79	4,82	4,69	1,08	0,80	0,74	0,1700	0,1203	0,0641	0,2538	0,2810	0,2918
RPL27	60S ribosomal protein L27	4,52	4,69	4,80	1,48	1,87	1,26	0,3232	0,0643	0,5091	0,3185	0,2293	0,4794
RARS	Arginine--tRNA ligase, cytoplasmic	4,47	4,40	4,55	0,85	1,20	1,41	0,2316	0,0727	0,0644	0,2816	0,2356	0,2918
VIM	Vimentin	6,80	6,53	6,68	0,54	0,76	1,41	0,0645	0,4984	0,3925	0,2248	0,4619	0,4473
HYOU1	Hypoxia up-regulated protein 1	5,32	4,87	5,23	0,35	0,82	2,31	0,0647	0,3654	0,1373	0,2248	0,4200	0,3513
MT-CO2	Cytochrome c oxidase subunit 2	4,52	4,70	4,71	1,50	1,55	1,04	0,0649	0,1709	0,9090	0,2248	0,3057	0,6468
RPL6	60S ribosomal protein L6	4,66	4,86	5,01	1,59	2,26	1,43	0,5185	0,2749	0,0649	0,3756	0,3887	0,2918
MARS	Methionine--tRNA ligase, cytoplasmic	4,18	3,96	4,19	0,59	1,02	1,72	0,0651	0,8836	0,0865	0,2248	0,5472	0,3018
PSMB1	Proteasome subunit beta type-1	4,16	4,78	4,78	4,16	4,14	0,99	0,0797	0,0652	0,9858	0,2248	0,2293	0,6619
NUDC	Nuclear migration protein nudC	4,49	4,40	4,33	0,82	0,69	0,84	0,0655	0,4694	0,7203	0,2248	0,4550	0,5771
GANAB	Neutral alpha-glucosidase AB	5,46	5,41	5,59	0,90	1,35	1,51	0,4202	0,1673	0,0658	0,3481	0,3028	0,2918
HSPA5	78 kDa glucose-regulated protein	5,94	5,77	6,04	0,68	1,27	1,87	0,1202	0,2867	0,0658	0,2314	0,3914	0,2918
EEF1D	Elongation factor 1-delta	4,81	4,81	4,88	0,99	1,17	1,18	0,9336	0,2402	0,0660	0,4790	0,3640	0,2918
RPS9	40S ribosomal protein S9	4,82	4,92	5,08	1,25	1,82	1,45	0,4051	0,0666	0,2468	0,3448	0,2293	0,3987
NME1-NME2;NME2;NI	Nucleoside diphosphate kinase;Nucleoside diphosphate kinase B;Nucleo	4,86	5,06	5,07	1,58	1,64	1,03	0,5098	0,0675	0,9606	0,3714	0,2307	0,6584
MYH9	Myosin-9	5,34	4,88	5,27	0,35	0,84	2,42	0,1130	0,6985	0,0677	0,2283	0,5201	0,2918
EFTUD2	116 kDa U5 small nuclear ribonucleoprotein component	4,82	4,75	4,83	0,85	1,01	1,19	0,3462	0,9598	0,0680	0,3239	0,5624	0,2918
SRM	Spermidine synthase	4,19	4,27	4,41	1,20	1,67	1,39	0,4405	0,1073	0,0683	0,3524	0,2771	0,2918
RANBP1	Ran-specific GTPase-activating protein	4,21	4,51	4,31	2,00	1,26	0,63	0,0688	0,3366	0,1101	0,2248	0,4142	0,3354
MAP1B	Microtubule-associated protein 1B;MAP1B heavy chain;MAP1 light chair	6,19	6,02	5,54	0,68	0,23	0,33	0,0689	0,1522	0,2663	0,2248	0,2943	0,4135
RPS2	40S ribosomal protein S2	4,76	4,97	5,05	1,62	1,92	1,18	0,2396	0,1319	0,0689	0,2853	0,2810	0,2918
HIST2H2AC;HIST2H2A/	Histone H2A type 2-C;Histone H2A type 2-A	5,38	5,72	5,63	2,22	1,80	0,81	0,0901	0,0696	0,5059	0,2248	0,2322	0,4794
PEBP1	Phosphatidylethanolamine-binding protein 1;Hippocampal cholinergic ne	4,91	5,35	4,76	2,79	0,72	0,26	0,0698	0,6274	0,0797	0,2248	0,5017	0,2918
PSMC3	26S protease regulatory subunit 6A	4,54	4,70	4,61	1,46	1,17	0,80	0,2828	0,6168	0,0702	0,3074	0,4995	0,2918
CAPN1	Calpain-1 catalytic subunit	4,40	4,28	4,44	0,77	1,10	1,43	0,2388	0,7074	0,0710	0,2853	0,5201	0,2918
FLNA	Filamin-A	5,84	5,46	5,76	0,42	0,84	2,00	0,0712	0,5243	0,1222	0,2248	0,4695	0,3392
ACTR1B	Beta-centractin	4,23	4,28	4,14	1,11	0,82	0,73	0,3423	0,2533	0,0719	0,3236	0,3725	0,2918
RCN2	Reticulocalbin-2	4,61	4,70	4,55	1,21	0,86	0,71	0,3832	0,3577	0,0720	0,3337	0,4197	0,2918
SPTAN1	Spectrin alpha chain, non-erythrocytic 1	5,83	5,62	5,75	0,61	0,83	1,38	0,1977	0,0723	0,3912	0,2688	0,2356	0,4472
HSD17B10	3-hydroxyacyl-CoA dehydrogenase type-2	4,85	4,93	5,01	1,20	1,45	1,20	0,0729	0,0841	0,3079	0,2248	0,2497	0,4247
ACOX1	Peroxisomal acyl-coenzyme A oxidase 1	4,15	4,07	3,99	0,84	0,69	0,82	0,5080	0,0729	0,4538	0,3710	0,2356	0,4681
TNC	Tenascin	4,80	4,21	4,89	0,26	1,21	4,73	0,1479	0,8120	0,0732	0,2425	0,5333	0,2918
TRA2B	Transformer-2 protein homolog beta	4,29	4,46	4,34	1,48	1,12	0,75	0,4282	0,8109	0,0735	0,3505	0,5333	0,2918
PTMS	Parathymosin	3,48	4,26	3,96	5,98	3,01	0,50	0,0747	0,1129	0,4106	0,2248	0,2780	0,4542
ENO1	Alpha-enolase	5,87	6,14	6,03	1,87	1,47	0,79	0,0749	0,4311	0,6317	0,2248	0,4405	0,5402
SRP14	Signal recognition particle 14 kDa protein	4,45	4,88	4,68	2,67	1,69	0,63	0,0749	0,1301	0,2744	0,2248	0,2810	0,4183
ALDOA	Fructose-bisphosphate aldolase A	5,28	5,47	5,29	1,57	1,02	0,65	0,0751	0,9253	0,0840	0,2248	0,5568	0,3013
ACTR2	Actin-related protein 2	4,93	4,99	4,79	1,15	0,72	0,63	0,0751	0,3147	0,1736	0,2248	0,4115	0,3691
DDOST	Dolichyl-diphosphooligosaccharide--protein glycosyltransferase 48 kDa s	4,87	4,80	4,98	0,86	1,30	1,51	0,1986	0,2392	0,0754	0,2691	0,3640	0,2918
HTRA1	Serine protease HTRA1	4,71	4,05	4,81	0,22	1,28	5,71	0,1461	0,7407	0,0755	0,2425	0,5259	0,2918
GNB2	Guanine nucleotide-binding protein G(I)/G(S)/G(T) subunit beta-2	5,05	5,08	4,84	1,07	0,61	0,58	0,5434	0,1097	0,0766	0,3801	0,2771	0,2918
COPS5	COP9 signalosome complex subunit 5	3,99	4,21	4,13	1,64	1,36	0,83	0,0767	0,2294	0,4560	0,2248	0,3560	0,4681
AKR7A2	Aflatoxin B1 aldehyde reductase member 2	4,17	4,37	4,33	1,61	1,45	0,90	0,0769	0,1099	0,5261	0,2248	0,2771	0,4886

CAND1	Cullin-associated NEDD8-dissociated protein 1	5,43	5,47	5,33	1,09	0,81	0,74	0,6466	0,3642	0,0772	0,4050	0,4200	0,2918
GAPDH	Glyceraldehyde-3-phosphate dehydrogenase	6,26	6,41	6,18	1,43	0,84	0,59	0,0810	0,4578	0,0773	0,2248	0,4491	0,2918
PRKCSH	Glucosidase 2 subunit beta	4,84	4,71	4,93	0,74	1,24	1,66	0,0893	0,3451	0,0773	0,2248	0,4142	0,2918
COPG1	Coatomer subunit gamma-1	4,22	4,14	4,45	0,83	1,70	2,04	0,4474	0,1775	0,0778	0,3558	0,3070	0,2918
TUBB6	Tubulin beta-6 chain	5,41	5,03	5,13	0,42	0,53	1,27	0,0778	0,4772	0,8008	0,2248	0,4594	0,6098
HSPA4	Heat shock 70 kDa protein 4	5,10	5,11	5,02	1,03	0,83	0,81	0,8497	0,2874	0,0785	0,4582	0,3914	0,2918
CCAR1	Cell division cycle and apoptosis regulator protein 1	4,11	4,58	4,39	2,98	1,91	0,64	0,0978	0,0792	0,4075	0,2248	0,2497	0,4542
COPA	Coatomer subunit alpha;Xenin;Proxenin	4,87	4,63	4,91	0,57	1,09	1,91	0,0792	0,6270	0,0847	0,2248	0,5017	0,3016
USP7	Ubiquitin carboxyl-terminal hydrolase 7;Ubiquitin carboxyl-terminal hydr	4,33	4,17	4,02	0,70	0,49	0,71	0,0888	0,0792	0,2871	0,2248	0,2497	0,4247
DCLK2	Serine/threonine-protein kinase DCLK2	5,17	5,31	4,90	1,39	0,53	0,38	0,5081	0,0829	0,0799	0,3710	0,2497	0,2918
YWHAH	14-3-3 protein eta	4,53	4,61	4,22	1,20	0,49	0,41	0,4797	0,1840	0,0802	0,3631	0,3081	0,2918
RPL10A	60S ribosomal protein L10a	4,60	4,80	4,89	1,56	1,94	1,24	0,2316	0,0812	0,4551	0,2816	0,2497	0,4681
RPL12	60S ribosomal protein L12	4,20	4,49	4,50	1,97	2,02	1,03	0,0911	0,0813	0,9293	0,2248	0,2497	0,6545
EPB41L3	Band 4.1-like protein 3;Band 4.1-like protein 3, N-terminally processed	4,88	3,83	4,45	0,09	0,38	4,19	0,0816	0,3679	0,3681	0,2248	0,4200	0,4420
RPL14	60S ribosomal protein L14	4,47	4,55	4,67	1,20	1,58	1,31	0,4713	0,0818	0,3429	0,3631	0,2497	0,4358
VAT1L	Synaptic vesicle membrane protein VAT-1 homolog-like	4,55	4,44	3,86	0,78	0,20	0,26	0,7520	0,0820	0,1116	0,4325	0,2497	0,3354
EIF4G1	Eukaryotic translation initiation factor 4 gamma 1	4,47	4,30	4,45	0,68	0,97	1,42	0,0824	0,9030	0,2532	0,2248	0,5472	0,4050
RAB14	Ras-related protein Rab-14	4,60	4,78	4,61	1,55	1,04	0,68	0,0837	0,5615	0,1099	0,2248	0,4712	0,3354
RPL11	60S ribosomal protein L11	4,57	4,70	4,81	1,35	1,72	1,28	0,3353	0,0838	0,4808	0,3221	0,2497	0,4752
UQCRC1	Cytochrome b-c1 complex subunit 1, mitochondrial	4,58	4,44	4,46	0,74	0,76	1,04	0,0841	0,2370	0,8358	0,2248	0,3633	0,6219
RPL9	60S ribosomal protein L9	4,60	4,83	4,97	1,69	2,35	1,39	0,2426	0,0843	0,2142	0,2853	0,2497	0,3949
DNAJA1	DnaJ homolog subfamily A member 1	4,38	4,23	4,22	0,71	0,70	0,98	0,0844	0,1270	0,9104	0,2248	0,2810	0,6468
FSCN1	Fascin	5,43	5,57	5,45	1,37	1,05	0,76	0,0844	0,7813	0,1490	0,2248	0,5305	0,3570
VAPA	Vesicle-associated membrane protein-associated protein A	4,52	4,68	4,48	1,46	0,92	0,63	0,0846	0,8199	0,2031	0,2248	0,5343	0,3887
HSP90AB1	Heat shock protein HSP 90-beta	5,65	5,68	5,77	1,07	1,30	1,22	0,5601	0,0852	0,1001	0,3838	0,2498	0,3265
PPA1	Inorganic pyrophosphatase	4,83	5,07	4,82	1,76	0,98	0,56	0,0852	0,9550	0,1630	0,2248	0,5610	0,3626
CYB5B	Cytochrome b5 type B	4,52	4,71	4,73	1,53	1,62	1,06	0,0968	0,0858	0,7309	0,2248	0,2498	0,5823
TUFM	Elongation factor Tu, mitochondrial	5,29	5,21	5,25	0,83	0,91	1,10	0,1417	0,0861	0,4298	0,2397	0,2498	0,4615
ETF1	Eukaryotic peptide chain release factor subunit 1	4,19	4,15	4,24	0,92	1,12	1,22	0,7441	0,6761	0,0867	0,4295	0,5151	0,3018
ANXA6	Annexin A6;Annexin	5,61	5,37	5,81	0,58	1,59	2,73	0,2505	0,1479	0,0887	0,2872	0,2924	0,3067
CALM2;CALM1;CALM3	Calmodulin	4,84	5,44	5,14	3,93	1,97	0,50	0,0888	0,3741	0,2427	0,2248	0,4214	0,3949
DLD	Dihydrolipoyl dehydrogenase, mitochondrial	4,58	4,29	4,55	0,51	0,94	1,84	0,0904	0,8123	0,1621	0,2248	0,5333	0,3626
UBB;RPS27A;UBC;UBA	Ubiquitin-60S ribosomal protein L40;Ubiquitin;60S ribosomal protein L40	5,44	5,64	5,50	1,61	1,15	0,71	0,0915	0,5203	0,2595	0,2248	0,4684	0,4124
UCHL1	Ubiquitin carboxyl-terminal hydrolase isozyme L1	5,26	5,53	5,27	1,83	1,01	0,55	0,1700	0,9857	0,0922	0,2538	0,5712	0,3165
ATP2A2	Sarcoplasmic/endoplasmic reticulum calcium ATPase 2	4,91	4,60	4,98	0,50	1,17	2,36	0,0925	0,7729	0,2252	0,2248	0,5305	0,3949
ANP32A	Acidic leucine-rich nuclear phosphoprotein 32 family member A	4,59	4,96	4,73	2,32	1,36	0,59	0,0926	0,6635	0,4553	0,2248	0,5112	0,4681
HARS	Histidine--tRNA ligase, cytoplasmic	4,46	4,34	4,42	0,77	0,92	1,19	0,0928	0,7400	0,4994	0,2248	0,5259	0,4781
CAPRIN1	Caprin-1	4,23	4,03	4,10	0,63	0,74	1,17	0,0931	0,2494	0,5634	0,2248	0,3694	0,5059
EIF2S1	Eukaryotic translation initiation factor 2 subunit 1	4,22	4,06	4,12	0,70	0,79	1,14	0,1029	0,0934	0,4347	0,2253	0,2643	0,4621
HADH	Hydroxyacyl-coenzyme A dehydrogenase, mitochondrial	4,65	4,80	4,86	1,42	1,62	1,15	0,0985	0,0934	0,5489	0,2248	0,2643	0,4987
HADHA	Trifunctional enzyme subunit alpha, mitochondrial;Long-chain enoyl-CoA	5,19	4,93	5,29	0,54	1,24	2,28	0,1698	0,3831	0,0936	0,2538	0,4226	0,3180
DBN1	Drebrin	5,14	5,11	4,89	0,93	0,55	0,60	0,5912	0,0937	0,1318	0,3926	0,2643	0,3491
ERGIC1	Endoplasmic reticulum-Golgi intermediate compartment protein 1	4,23	4,37	4,32	1,39	1,24	0,89	0,0942	0,2418	0,3853	0,2248	0,3640	0,4454
GARS	Glycine--tRNA ligase	4,73	4,60	4,71	0,73	0,96	1,31	0,0942	0,8400	0,1541	0,2248	0,5383	0,3622
UFL1	E3 UFM1-protein ligase 1	3,74	3,68	3,88	0,86	1,39	1,62	0,5941	0,0948	0,1436	0,3926	0,2643	0,3531
LRP1	Prolow-density lipoprotein receptor-related protein 1;Low-density lipopr	4,72	4,16	4,29	0,28	0,38	1,35	0,0952	0,2675	0,6960	0,2248	0,3828	0,5695
PFKM	ATP-dependent 6-phosphofructokinase, muscle type	4,94	5,04	4,96	1,27	1,07	0,84	0,0953	0,8140	0,4889	0,2248	0,5333	0,4752
LRPPRC	Leucine-rich PPR motif-containing protein, mitochondrial	4,91	4,10	4,65	0,15	0,55	3,59	0,0955	0,2603	0,2394	0,2248	0,3799	0,3949
TNPO1	Transportin-1	4,57	4,55	4,49	0,97	0,84	0,87	0,9170	0,5975	0,0959	0,4751	0,4914	0,3210
EEF1A1;EEF1A1P5	Elongation factor 1-alpha 1;Putative elongation factor 1-alpha-like 3	6,04	6,06	6,16	1,05	1,30	1,25	0,6642	0,0960	0,1125	0,4084	0,2658	0,3354
VTA1	Vacuolar protein sorting-associated protein VTA1 homolog	4,15	4,18	4,07	1,09	0,84	0,77	0,5830	0,3635	0,0961	0,3926	0,4200	0,3210
ANXA2;ANXA2P2	Annexin A2;Annexin;Putative annexin A2-like protein	5,82	5,52	5,93	0,49	1,28	2,59	0,2719	0,5464	0,0979	0,3031	0,4701	0,3250
SRSF3	Serine/arginine-rich splicing factor 3	4,38	4,70	4,52	2,09	1,38	0,66	0,0989	0,3478	0,1601	0,2248	0,4150	0,3626
ALDH2	Aldehyde dehydrogenase, mitochondrial	4,90	4,84	4,70	0,86	0,63	0,73	0,4542	0,0995	0,1709	0,3581	0,2702	0,3685
ALDH7A1	Alpha-aminoadipic semialdehyde dehydrogenase	4,91	4,95	5,04	1,09	1,34	1,24	0,5851	0,0995	0,2684	0,3926	0,2702	0,4146
EIF4G2	Eukaryotic translation initiation factor 4 gamma 2	4,61	4,62	4,49	1,00	0,75	0,75	0,9812	0,1232	0,1004	0,4917	0,2810	0,3265
CDC37	Hsp90 co-chaperone Cdc37;Hsp90 co-chaperone Cdc37, N-terminally pro	4,45	4,48	4,37	1,07	0,83	0,78	0,7554	0,1006	0,2767	0,4331	0,2715	0,4183

RAB5A	Ras-related protein Rab-5A	4,64	4,75	4,63	1,30	0,99	0,76	0,2844	0,9535	0,1010	0,3078	0,5609	0,3265
HSD17B4	Peroxisomal multifunctional enzyme type 2;(3R)-hydroxyacyl-CoA dehyd	4,94	4,80	4,81	0,71	0,74	1,03	0,1013	0,3282	0,9112	0,2253	0,4137	0,6468
ACBD3	Golgi resident protein GCP60	3,63	3,46	3,65	0,69	1,06	1,54	0,1019	0,7995	0,1873	0,2253	0,5305	0,3790
HIST1H4A	Histone H4	5,92	6,43	6,14	3,19	1,67	0,52	0,1024	0,1320	0,2812	0,2253	0,2810	0,4223
MDH1	Malate dehydrogenase, cytoplasmic	5,02	5,26	5,05	1,71	1,06	0,62	0,1041	0,8958	0,1824	0,2253	0,5472	0,3757
RNF40	E3 ubiquitin-protein ligase BRE1B	3,38	3,30	3,34	0,84	0,91	1,08	0,1048	0,4856	0,6056	0,2253	0,4608	0,5283
FLNC	Filamin-C	5,53	4,84	5,59	0,20	1,14	5,63	0,1048	0,8773	0,1843	0,2253	0,5472	0,3772
ATP5B	ATP synthase subunit beta, mitochondrial;ATP synthase subunit beta	6,15	6,04	6,07	0,76	0,83	1,09	0,1053	0,3608	0,6492	0,2253	0,4200	0,5480
SERPINB6	Serpin B6	4,10	4,13	4,27	1,06	1,47	1,38	0,6513	0,1058	0,2024	0,4056	0,2771	0,3887
ACADM	Medium-chain specific acyl-CoA dehydrogenase, mitochondrial	4,01	4,03	4,21	1,03	1,56	1,51	0,9316	0,1060	0,3751	0,4786	0,2771	0,4438
ALDH18A1	Delta-1-pyrroline-5-carboxylate synthase;Glutamate 5-kinase;Gamma-gl	4,44	4,16	4,50	0,52	1,15	2,22	0,1063	0,7984	0,2428	0,2261	0,5305	0,3949
ARHGDIA	Rho GDP-dissociation inhibitor 1	4,78	4,97	4,90	1,56	1,32	0,85	0,1081	0,3871	0,4735	0,2265	0,4238	0,4736
H1FX	Histone H1x	4,54	5,09	4,62	3,52	1,18	0,34	0,1176	0,7739	0,1084	0,2301	0,5305	0,3354
MYL12A;MYL12B;MYL	Myosin regulatory light chain 12A;Myosin regulatory light chain 12B;Myo	4,25	4,39	4,53	1,39	1,91	1,38	0,2364	0,1085	0,4445	0,2853	0,2771	0,4665
CDC2;CDK1	Cyclin-dependent kinase 1	4,40	4,13	4,14	0,54	0,55	1,03	0,1088	0,1443	0,8928	0,2265	0,2911	0,6445
DARS	Aspartate--tRNA ligase, cytoplasmic	4,83	4,92	4,98	1,23	1,41	1,15	0,1485	0,1095	0,3468	0,2425	0,2771	0,4358
ADD1	Alpha-adducin	4,78	4,57	4,60	0,62	0,65	1,05	0,1100	0,1453	0,8403	0,2272	0,2911	0,6234
ARF4	ADP-ribosylation factor 4	4,26	4,45	4,62	1,52	2,26	1,49	0,2486	0,1107	0,4607	0,2872	0,2771	0,4681
USP5	Ubiquitin carboxyl-terminal hydrolase 5	4,95	5,03	4,97	1,20	1,05	0,87	0,2726	0,7810	0,1109	0,3031	0,5305	0,3354
GOT1	Aspartate aminotransferase, cytoplasmic	4,65	4,61	4,43	0,92	0,60	0,66	0,7794	0,1123	0,1438	0,4393	0,2780	0,3531
SCARB2	Lysosome membrane protein 2	4,81	4,58	4,62	0,59	0,65	1,09	0,1124	0,2632	0,8260	0,2283	0,3806	0,6188
DDX5	Probable ATP-dependent RNA helicase DDX5	4,96	4,87	4,82	0,82	0,73	0,89	0,2823	0,1286	0,1124	0,3074	0,2810	0,3354
TARS	Threonine--tRNA ligase, cytoplasmic	4,44	4,18	4,48	0,55	1,09	1,99	0,1937	0,7846	0,1131	0,2681	0,5305	0,3354
OTUB1	Ubiquitin thioesterase OTUB1	4,47	4,65	4,52	1,52	1,13	0,74	0,1132	0,6158	0,2260	0,2283	0,4995	0,3949
CCT2	T-complex protein 1 subunit beta	5,51	5,55	5,50	1,08	0,96	0,89	0,5384	0,7197	0,1137	0,3795	0,5213	0,3354
CRMP1	Dihydropyrimidinase-related protein 1	6,15	6,21	5,51	1,17	0,23	0,20	0,4238	0,1483	0,1139	0,3492	0,2924	0,3354
G3BP2	Ras GTPase-activating protein-binding protein 2	4,22	4,15	4,03	0,85	0,64	0,76	0,3999	0,1143	0,2367	0,3425	0,2792	0,3949
ERLIN2	Erlin-2	4,85	4,64	4,85	0,60	0,99	1,63	0,1144	0,9353	0,1718	0,2283	0,5568	0,3685
MARCKS	Myristoylated alanine-rich C-kinase substrate	4,75	4,52	4,29	0,60	0,35	0,58	0,2228	0,1147	0,2783	0,2791	0,2792	0,4192
NPM1	Nucleophosmin	5,38	5,33	5,42	0,90	1,11	1,23	0,5473	0,5414	0,1151	0,3806	0,4701	0,3354
SCRN1	Secernin-1	4,81	4,96	4,65	1,41	0,69	0,49	0,2194	0,3512	0,1154	0,2791	0,4156	0,3354
HIST2H2BE;HIST1H2BE	Histone H2B type 2-E;Histone H2B type 1-B;Histone H2B type 1-O;Histon	4,90	5,35	4,91	2,81	1,02	0,36	0,1163	0,9747	0,2295	0,2299	0,5664	0,3949
ATP5A1	ATP synthase subunit alpha, mitochondrial	6,07	5,94	5,98	0,73	0,81	1,11	0,1183	0,3276	0,6210	0,2301	0,4137	0,5370
PSME2	Proteasome activator complex subunit 2	3,96	4,30	4,53	2,20	3,67	1,67	0,2297	0,1185	0,4284	0,2816	0,2810	0,4615
SPTBN1	Spectrin beta chain, non-erythrocytic 1	5,65	4,99	5,54	0,22	0,77	3,56	0,1298	0,1190	0,1930	0,2363	0,2810	0,3828
PSMA7;PSMA8	Proteasome subunit alpha type-7;Proteasome subunit alpha type-7-like	4,61	4,82	4,70	1,60	1,22	0,76	0,1199	0,4925	0,2242	0,2314	0,4608	0,3949
AP2A2	AP-2 complex subunit alpha-2	4,83	4,68	4,67	0,72	0,71	0,98	0,3257	0,1199	0,9528	0,3185	0,2810	0,6568
SLC25A5	ADP/ATP translocase 2;ADP/ATP translocase 2, N-terminally processed	5,07	5,11	5,24	1,10	1,49	1,35	0,2917	0,1201	0,1811	0,3115	0,2810	0,3754
CCT6A	T-complex protein 1 subunit zeta	5,47	5,47	5,41	1,00	0,88	0,88	0,9741	0,3194	0,1209	0,4917	0,4137	0,3392
HNRNPA3	Heterogeneous nuclear ribonucleoprotein A3	5,33	5,36	5,25	1,07	0,84	0,78	0,5915	0,1794	0,1212	0,3926	0,3070	0,3392
PAFAH1B1	Platelet-activating factor acetylhydrolase IB subunit alpha	4,72	4,77	4,50	1,12	0,60	0,53	0,5594	0,2317	0,1212	0,3838	0,3570	0,3392
PAICS	Multifunctional protein ADE2;Phosphoribosylaminoimidazole-succinocar	4,89	5,00	4,67	1,27	0,59	0,47	0,3512	0,2066	0,1220	0,3239	0,3352	0,3392
SFPQ	Splicing factor, proline- and glutamine-rich	5,40	5,34	5,30	0,87	0,81	0,93	0,1405	0,1229	0,4169	0,2397	0,2810	0,4581
RPL13	60S ribosomal protein L13	4,67	4,79	4,88	1,33	1,63	1,23	0,3330	0,1230	0,2664	0,3221	0,2810	0,4135
APMAP	Adipocyte plasma membrane-associated protein	4,86	4,67	4,53	0,64	0,47	0,73	0,2505	0,1248	0,3949	0,2872	0,2810	0,4479
LDHB	L-lactate dehydrogenase B chain;L-lactate dehydrogenase	5,70	5,93	5,79	1,71	1,24	0,72	0,1251	0,5549	0,3038	0,2357	0,4701	0,4247
ASAH1	Acid ceramidase;Acid ceramidase subunit alpha;Acid ceramidase subunit	4,55	4,37	4,41	0,66	0,72	1,09	0,3622	0,1251	0,8266	0,3280	0,2810	0,6188
PSMA3	Proteasome subunit alpha type-3	4,18	4,54	4,29	2,32	1,29	0,56	0,1254	0,5797	0,1972	0,2357	0,4824	0,3859
HNRNPK	Heterogeneous nuclear ribonucleoprotein K	5,88	5,97	5,85	1,24	0,94	0,76	0,3341	0,7664	0,1262	0,3221	0,5296	0,3411
LAMP1	Lysosome-associated membrane glycoprotein 1	4,92	4,49	4,85	0,37	0,84	2,27	0,1263	0,3824	0,1667	0,2357	0,4226	0,3654
FXR1	Fragile X mental retardation syndrome-related protein 1	4,15	4,04	4,23	0,78	1,20	1,54	0,1288	0,7560	0,4766	0,2363	0,5288	0,4746
CYFIP2	Cytoplasmic FMR1-interacting protein 2	4,79	4,62	4,46	0,68	0,47	0,69	0,2148	0,1301	0,4472	0,2791	0,2810	0,4665
AHSA1	Activator of 90 kDa heat shock protein ATPase homolog 1	4,48	4,43	4,23	0,89	0,56	0,63	0,5353	0,1302	0,1931	0,3789	0,2810	0,3828
ETFA	Electron transfer flavoprotein subunit alpha, mitochondrial	4,37	4,54	4,58	1,46	1,60	1,10	0,2195	0,1304	0,6071	0,2791	0,2810	0,5287
AHCY	Adenosylhomocysteinase	5,13	5,08	5,16	0,90	1,09	1,21	0,4750	0,5559	0,1312	0,3631	0,4701	0,3491
RPLP2	60S acidic ribosomal protein P2	4,29	4,73	4,60	2,74	2,04	0,74	0,1473	0,1320	0,5936	0,2425	0,2810	0,5215
UGGT1	UDP-glucose:glycoprotein glucosyltransferase 1	4,86	4,52	4,61	0,45	0,56	1,24	0,2211	0,1327	0,7099	0,2791	0,2810	0,5715

AKAP12	A-kinase anchor protein 12	4,37	3,84	3,85	0,30	0,31	1,04	0,1560	0,1343	0,9530	0,2459	0,2815	0,6568
TFRC	Transferrin receptor protein 1;Transferrin receptor protein 1, serum forr	3,97	4,12	4,02	1,40	1,12	0,80	0,1354	0,6662	0,3547	0,2397	0,5121	0,4375
PSMA1	Proteasome subunit alpha type-1;Proteasome subunit alpha type	4,37	4,59	4,49	1,65	1,33	0,81	0,1356	0,4913	0,5404	0,2397	0,4608	0,4970
G6PD	Glucose-6-phosphate 1-dehydrogenase	4,38	4,18	4,09	0,64	0,51	0,81	0,1358	0,2753	0,7080	0,2397	0,3887	0,5715
FABP5	Fatty acid-binding protein, epidermal	4,25	4,64	4,31	2,45	1,14	0,46	0,1359	0,7905	0,2228	0,2397	0,5305	0,3949
RPS17L;RPS17	40S ribosomal protein S17-like;40S ribosomal protein S17	4,50	4,73	4,71	1,69	1,60	0,95	0,1366	0,1433	0,8647	0,2397	0,2908	0,6363
XPNPEP1	Xaa-Pro aminopeptidase 1	4,33	4,39	4,27	1,15	0,88	0,76	0,5865	0,6155	0,1366	0,3926	0,4995	0,3512
TOP1	DNA topoisomerase 1	4,40	4,25	4,31	0,70	0,81	1,16	0,1382	0,1820	0,4619	0,2397	0,3071	0,4681
ERP29	Endoplasmic reticulum resident protein 29	4,51	4,58	4,65	1,18	1,39	1,19	0,3218	0,1384	0,3402	0,3185	0,2881	0,4358
CLIP2	CAP-Gly domain-containing linker protein 2	4,20	4,28	4,04	1,20	0,69	0,58	0,6266	0,4436	0,1390	0,3982	0,4464	0,3520
IQGAP1	Ras GTPase-activating-like protein IQGAP1	4,74	4,32	4,95	0,38	1,64	4,30	0,3423	0,4469	0,1398	0,3236	0,4486	0,3520
CPNE3	Copine-3	4,26	4,34	4,32	1,20	1,14	0,94	0,1407	0,2424	0,6752	0,2397	0,3640	0,5592
PLEC	Plectin	5,65	4,61	5,44	0,09	0,62	6,68	0,1410	0,1435	0,2179	0,2397	0,2908	0,3949
ST13;ST13P5;ST13P4	Hsc70-interacting protein;Putative protein FAM10A5;Putative protein FA	4,94	5,03	4,96	1,24	1,05	0,85	0,1415	0,7406	0,3032	0,2397	0,5259	0,4247
SRSF7	Serine/arginine-rich splicing factor 7	4,59	4,77	4,58	1,51	0,96	0,64	0,2262	0,8472	0,1415	0,2815	0,5393	0,3531
USP9X	Probable ubiquitin carboxyl-terminal hydrolase FAF-X	4,07	3,87	4,02	0,62	0,89	1,42	0,1930	0,7424	0,1417	0,2681	0,5259	0,3531
LAMP2	Lysosome-associated membrane glycoprotein 2	4,66	4,33	4,59	0,47	0,85	1,83	0,1426	0,4578	0,2034	0,2397	0,4491	0,3887
RAB5B	Ras-related protein Rab-5B	4,06	4,13	4,19	1,17	1,34	1,15	0,4312	0,1685	0,1434	0,3505	0,3037	0,3531
NPTN;DKFZp566H1924	Neuroplastin	4,57	5,08	4,56	3,25	0,98	0,30	0,1545	0,9615	0,1460	0,2451	0,5625	0,3554
STIP1	Stress-induced-phosphoprotein 1	4,93	4,99	4,89	1,14	0,90	0,79	0,4005	0,4104	0,1482	0,3425	0,4346	0,3570
OLA1	Obg-like ATPase 1	4,81	4,94	4,78	1,34	0,93	0,70	0,1780	0,7627	0,1485	0,2584	0,5288	0,3570
DPYSL3	Dihydropyrimidinase-related protein 3	6,16	6,20	5,64	1,10	0,30	0,27	0,5430	0,1782	0,1501	0,3801	0,3070	0,3579
ESD	S-formylglutathione hydrolase	4,34	4,51	4,47	1,45	1,34	0,92	0,1502	0,3625	0,7276	0,2432	0,4200	0,5810
MAP2	Microtubule-associated protein 2	5,47	5,43	4,63	0,91	0,14	0,16	0,7728	0,1518	0,1639	0,4369	0,2943	0,3626
FTO	Alpha-ketoglutarate-dependent dioxygenase FTO	3,94	4,01	4,03	1,17	1,24	1,07	0,4761	0,1525	0,7477	0,3631	0,2943	0,5860
UBR4	E3 ubiquitin-protein ligase UBR4	4,19	4,28	4,21	1,24	1,04	0,84	0,4532	0,8885	0,1531	0,3581	0,5472	0,3622
COPB2	Coatomer subunit beta	4,47	4,22	4,41	0,56	0,88	1,56	0,1538	0,7716	0,1933	0,2451	0,5305	0,3828
HMGCS1	Hydroxymethylglutaryl-CoA synthase, cytoplasmic	4,99	5,18	4,82	1,52	0,67	0,44	0,3758	0,5523	0,1538	0,3328	0,4701	0,3622
CTNNA2	Catenin alpha-2	5,00	4,85	4,55	0,71	0,36	0,50	0,3448	0,1545	0,3470	0,3239	0,2956	0,4358
AIFM1	Apoptosis-inducing factor 1, mitochondrial	4,04	3,80	3,98	0,57	0,87	1,53	0,1548	0,5599	0,2400	0,2451	0,4712	0,3949
LMNB1	Lamin-B1	5,43	5,49	5,26	1,16	0,67	0,58	0,4935	0,2583	0,1558	0,3669	0,3787	0,3626
GDI2	Rab GDP dissociation inhibitor beta	5,79	5,87	5,67	1,19	0,75	0,63	0,1636	0,3750	0,1559	0,2514	0,4214	0,3626
ATIC	Bifunctional purine biosynthesis protein PURH;Phosphoribosylaminoimid	4,63	4,54	4,71	0,83	1,22	1,47	0,5970	0,1563	0,3131	0,3926	0,2960	0,4271
DLST	Dihydrolipoyllysine-residue succinyltransferase component of 2-oxogluta	4,80	4,66	4,65	0,72	0,71	0,98	0,1684	0,1565	0,9258	0,2538	0,2960	0,6542
PLRG1	Pleiotropic regulator 1	3,50	3,44	3,42	0,86	0,83	0,96	0,1566	0,3403	0,8297	0,2459	0,4142	0,6193
AP2M1	AP-2 complex subunit mu	4,35	4,50	4,30	1,39	0,89	0,64	0,3782	0,7850	0,1579	0,3328	0,5305	0,3626
WDR1	WD repeat-containing protein 1	5,02	4,95	4,90	0,86	0,77	0,89	0,4399	0,1596	0,4412	0,3524	0,2982	0,4665
U2AF2	Splicing factor U2AF 65 kDa subunit	4,79	4,83	4,64	1,10	0,72	0,66	0,8056	0,1598	0,2724	0,4460	0,2982	0,4181
H2AFV;H2AFZ	Histone H2A.V;Histone H2A.Z;Histone H2A	4,43	4,80	4,61	2,36	1,52	0,64	0,1617	0,2460	0,4454	0,2506	0,3682	0,4665
DPYSL2	Dihydropyrimidinase-related protein 2	6,16	6,15	5,68	0,99	0,34	0,34	0,9423	0,1624	0,1632	0,4825	0,3018	0,3626
ACLY	ATP-citrate synthase	4,84	4,71	4,92	0,74	1,19	1,59	0,4813	0,6567	0,1633	0,3631	0,5104	0,3626
IGF2BP1	Insulin-like growth factor 2 mRNA-binding protein 1	4,85	4,71	4,48	0,71	0,43	0,60	0,6031	0,1662	0,4287	0,3926	0,3027	0,4615
RUVBL1	RuvB-like 1	4,77	4,86	4,83	1,23	1,15	0,94	0,1667	0,3229	0,3479	0,2538	0,4137	0,4359
VCP	Transitional endoplasmic reticulum ATPase	5,57	5,57	5,53	0,98	0,90	0,91	0,8750	0,3391	0,1693	0,4666	0,4142	0,3685
DDAH2	N(G),N(G)-dimethylarginine dimethylaminohydrolase 2	4,66	4,96	4,50	1,99	0,68	0,34	0,1707	0,6014	0,1809	0,2538	0,4937	0,3754
DNAJB11	DnaJ homolog subfamily B member 11	4,25	4,22	4,41	0,93	1,42	1,52	0,8468	0,3651	0,1717	0,4582	0,4200	0,3685
RPS16	40S ribosomal protein S16	4,53	4,70	4,76	1,47	1,66	1,13	0,3365	0,1720	0,7771	0,3221	0,3057	0,5991
HNRNPA1;HNRNPA1L2	Heterogeneous nuclear ribonucleoprotein A1;Heterogeneous nuclear rib	5,66	5,66	5,56	1,00	0,79	0,79	0,9791	0,1725	0,2063	0,4917	0,3057	0,3927
ENAH	Protein enabled homolog	3,68	3,27	3,26	0,39	0,38	0,98	0,1749	0,1753	0,9674	0,2560	0,3070	0,6595
PDHA1	Pyruvate dehydrogenase E1 component subunit alpha, somatic form, mi	4,69	4,31	4,55	0,42	0,74	1,77	0,1749	0,5099	0,2894	0,2560	0,4675	0,4247
C1QBP	Complement component 1 Q subcomponent-binding protein, mitochond	4,34	4,38	4,60	1,10	1,82	1,66	0,6287	0,1750	0,2180	0,3986	0,3070	0,3949
DCLK1	Serine/threonine-protein kinase DCLK1	5,34	5,23	4,98	0,77	0,44	0,57	0,1761	0,1816	0,3464	0,2566	0,3071	0,4358
LRRC47	Leucine-rich repeat-containing protein 47	4,55	4,54	4,43	0,96	0,75	0,78	0,8478	0,1763	0,1972	0,4582	0,3070	0,3859
PDHB	Pyruvate dehydrogenase E1 component subunit beta, mitochondrial	4,73	4,70	4,82	0,93	1,23	1,31	0,7621	0,4193	0,1765	0,4346	0,4365	0,3720
DPYSL5	Dihydropyrimidinase-related protein 5	5,66	5,63	4,84	0,92	0,15	0,16	0,7258	0,1769	0,1924	0,4291	0,3070	0,3828
MAP1A	Microtubule-associated protein 1A;MAP1A heavy chain;MAP1 light chair	4,15	3,98	3,99	0,68	0,69	1,01	0,2421	0,1791	0,9612	0,2853	0,3070	0,6584
MUT	Methylmalonyl-CoA mutase, mitochondrial	4,12	4,09	4,19	0,94	1,16	1,23	0,6826	0,4310	0,1800	0,4147	0,4405	0,3754

NES	Nestin	5,83	5,62	5,76	0,62	0,84	1,36	0,1814	0,5560	0,3735	0,2587	0,4701	0,4438
HSPA9	Stress-70 protein, mitochondrial	5,36	5,27	5,39	0,81	1,07	1,33	0,1818	0,7240	0,2173	0,2587	0,5225	0,3949
ANP32E	Acidic leucine-rich nuclear phosphoprotein 32 family member E	4,26	4,49	4,40	1,73	1,39	0,80	0,1824	0,1904	0,5460	0,2587	0,3163	0,4987
GLB1	Beta-galactosidase	4,46	4,09	4,26	0,43	0,64	1,47	0,1825	0,2782	0,3997	0,2587	0,3891	0,4487
HNRNPA2B1	Heterogeneous nuclear ribonucleoproteins A2/B1	5,74	5,84	5,69	1,26	0,90	0,72	0,3474	0,7203	0,1828	0,3239	0,5213	0,3757
PDS5B	Sister chromatid cohesion protein PDS5 homolog B	4,08	4,11	4,16	1,07	1,21	1,13	0,6588	0,1830	0,3420	0,4078	0,3076	0,4358
ME2	NAD-dependent malic enzyme, mitochondrial	4,54	4,36	4,30	0,67	0,58	0,86	0,3142	0,1850	0,4915	0,3185	0,3085	0,4752
IARS	Isoleucine--tRNA ligase, cytoplasmic	4,43	4,29	4,73	0,73	2,00	2,74	0,2478	0,3389	0,1860	0,2872	0,4142	0,3790
CCT4	T-complex protein 1 subunit delta	5,34	5,39	5,34	1,11	1,00	0,90	0,4187	0,9881	0,1875	0,3481	0,5713	0,3790
DDAH1	N(G),N(G)-dimethylarginine dimethylaminohydrolase 1	5,00	5,18	4,70	1,55	0,51	0,33	0,1888	0,5268	0,3023	0,2656	0,4701	0,4247
CHTOP	Chromatin target of PRMT1 protein	3,78	3,85	3,78	1,17	0,99	0,84	0,1924	0,9692	0,4987	0,2681	0,5645	0,4781
PGLS	6-phosphogluconolactonase	4,59	4,75	4,22	1,45	0,43	0,29	0,3422	0,3702	0,1925	0,3236	0,4203	0,3828
NUDT21	Cleavage and polyadenylation specificity factor subunit 5	4,27	4,68	4,53	2,58	1,85	0,72	0,1950	0,4052	0,5355	0,2681	0,4337	0,4934
ENO2	Gamma-enolase;Enolase	5,26	5,52	4,47	1,83	0,16	0,09	0,2061	0,3218	0,1951	0,2750	0,4137	0,3848
ACOT7	Cytosolic acyl coenzyme A thioester hydrolase	5,17	5,11	4,62	0,86	0,28	0,32	0,6178	0,1962	0,2396	0,3979	0,3234	0,3949
EIF4E	Eukaryotic translation initiation factor 4E	3,93	4,07	4,00	1,39	1,17	0,84	0,1970	0,4963	0,2351	0,2688	0,4612	0,3949
DDB1	DNA damage-binding protein 1	4,58	4,53	4,71	0,89	1,34	1,51	0,7900	0,5521	0,1982	0,4412	0,4701	0,3863
NAPA	Alpha-soluble NSF attachment protein	4,28	4,48	4,46	1,59	1,53	0,96	0,2501	0,1986	0,8921	0,2872	0,3261	0,6445
CS	Citrate synthase;Citrate synthase, mitochondrial	5,27	5,18	5,11	0,82	0,70	0,85	0,2913	0,1998	0,4837	0,3115	0,3267	0,4752
EIF2S2	Eukaryotic translation initiation factor 2 subunit 2	3,99	3,96	4,15	0,93	1,43	1,54	0,8059	0,2690	0,2002	0,4460	0,3837	0,3886
PSMC6	26S protease regulatory subunit 10B	4,68	4,82	4,77	1,38	1,23	0,89	0,2017	0,3299	0,3293	0,2722	0,4137	0,4345
HNRNPA0	Heterogeneous nuclear ribonucleoprotein A0	4,88	5,02	4,80	1,39	0,84	0,60	0,2777	0,4964	0,2022	0,3069	0,4612	0,3887
FUS	RNA-binding protein FUS	4,86	4,77	4,73	0,81	0,74	0,92	0,2052	0,3311	0,7482	0,2750	0,4137	0,5860
NUP155	Nuclear pore complex protein Nup155	3,92	4,03	4,10	1,30	1,52	1,17	0,3594	0,2082	0,3625	0,3262	0,3353	0,4375
PABPC1;PABPC3	Polyadenylate-binding protein 1;Polyadenylate-binding protein 3	5,07	4,99	5,02	0,84	0,89	1,06	0,2089	0,4007	0,4272	0,2758	0,4318	0,4615
TUBB2B	Tubulin beta-2B chain	5,05	4,95	4,53	0,79	0,30	0,38	0,5546	0,2099	0,2624	0,3835	0,3355	0,4131
CAT	Catalase	4,06	3,70	3,86	0,43	0,62	1,43	0,2127	0,3778	0,3600	0,2791	0,4222	0,4375
RTN3	Reticulon-3	4,56	4,58	4,27	1,03	0,51	0,49	0,7651	0,2310	0,2144	0,4346	0,3570	0,3949
PSMB7	Proteasome subunit beta type-7	4,37	4,52	4,42	1,40	1,10	0,79	0,2146	0,8003	0,4660	0,2791	0,5305	0,4699
CAPZA1	F-actin-capping protein subunit alpha-1	4,79	4,89	4,78	1,27	0,98	0,77	0,2148	0,9467	0,4501	0,2791	0,5601	0,4681
BZW1	Basic leucine zipper and W2 domain-containing protein 1	4,37	4,33	4,48	0,91	1,27	1,40	0,5835	0,2783	0,2149	0,3926	0,3891	0,3949
02. Sep	Septin-2	5,07	5,11	5,07	1,11	0,99	0,90	0,2158	0,8702	0,2217	0,2791	0,5453	0,3949
ZNF512	Zinc finger protein 512	4,17	4,21	4,04	1,11	0,74	0,66	0,5287	0,3419	0,2172	0,3778	0,4142	0,3949
SF3A3	Splicing factor 3A subunit 3	4,07	4,15	4,10	1,21	1,07	0,88	0,2172	0,6638	0,3070	0,2791	0,5112	0,4247
ADK	Adenosine kinase	3,80	4,04	3,97	1,73	1,47	0,85	0,2173	0,3340	0,7651	0,2791	0,4142	0,5945
BUB3	Mitotic checkpoint protein BUB3	4,06	4,29	4,27	1,68	1,63	0,97	0,2283	0,2191	0,9374	0,2816	0,3476	0,6561
RAD23B	UV excision repair protein RAD23 homolog B	4,21	4,12	4,09	0,81	0,75	0,93	0,4170	0,2200	0,7321	0,3481	0,3477	0,5823
AGL	Glycogen debranching enzyme;4-alpha-glucanotransferase;Amylo-alpha	3,79	3,60	3,76	0,64	0,94	1,46	0,2216	0,8104	0,3025	0,2791	0,5333	0,4247
SEC31A	Protein transport protein Sec31A	4,22	4,11	4,36	0,79	1,39	1,77	0,2218	0,4144	0,2247	0,2791	0,4365	0,3949
PSIP1	PC4 and SFRS1-interacting protein	4,67	4,82	4,58	1,43	0,82	0,57	0,3887	0,6304	0,2245	0,3369	0,5020	0,3949
HIST1H1C	Histone H1.2	5,36	5,51	5,30	1,41	0,88	0,63	0,3250	0,4499	0,2253	0,3185	0,4491	0,3949
TCP1	T-complex protein 1 subunit alpha	5,25	5,24	5,22	0,97	0,92	0,95	0,7484	0,2269	0,5251	0,4311	0,3545	0,4886
HIST1H1B	Histone H1.5	4,94	5,18	5,13	1,72	1,55	0,90	0,3183	0,2273	0,7924	0,3185	0,3545	0,6052
PKM	Pyruvate kinase PKM;Pyruvate kinase	5,90	5,94	5,96	1,10	1,16	1,05	0,3802	0,2273	0,7335	0,3335	0,3545	0,5823
GPC4	Glypican-4;Secreted glypican-4	4,33	4,03	3,91	0,51	0,38	0,75	0,3250	0,2276	0,4103	0,3185	0,3545	0,4542
CBX3	Chromobox protein homolog 3	4,20	4,50	4,13	2,00	0,86	0,43	0,2446	0,7619	0,2281	0,2868	0,5288	0,3949
ASNA1	ATPase ASNA1	4,53	4,59	4,53	1,16	1,02	0,88	0,4298	0,8983	0,2284	0,3505	0,5472	0,3949
RPN1	Dolichyl-diphosphooligosaccharide--protein glycosyltransferase subunit	5,26	5,15	5,27	0,78	1,03	1,33	0,2284	0,9057	0,2605	0,2816	0,5481	0,4124
PYGL	Glycogen phosphorylase, liver form;Phosphorylase	4,15	3,90	4,07	0,55	0,83	1,50	0,2302	0,6981	0,4774	0,2816	0,5201	0,4746
HNRNPD	Heterogeneous nuclear ribonucleoprotein D0	5,31	5,39	5,24	1,21	0,85	0,71	0,4287	0,6153	0,2303	0,3505	0,4995	0,3949
ATP1A1	Sodium/potassium-transporting ATPase subunit alpha-1	4,40	4,21	4,40	0,66	1,01	1,53	0,4854	0,9923	0,2313	0,3631	0,5726	0,3949
TKT	Transketolase	5,49	5,55	5,45	1,15	0,92	0,80	0,2317	0,8303	0,5980	0,2816	0,5361	0,5244
ACAT1	Acetyl-CoA acetyltransferase, mitochondrial	4,79	4,70	4,60	0,81	0,64	0,79	0,2327	0,5340	0,7339	0,2818	0,4701	0,5823
HNRNPAB	Heterogeneous nuclear ribonucleoprotein A/B	4,81	4,97	4,76	1,44	0,89	0,62	0,3151	0,7474	0,2334	0,3185	0,5268	0,3949
MAPK1	Mitogen-activated protein kinase 1	4,58	4,68	4,52	1,24	0,87	0,70	0,5216	0,7330	0,2363	0,3756	0,5245	0,3949
CHD4	Chromodomain-helicase-DNA-binding protein 4	4,42	4,30	4,38	0,77	0,91	1,18	0,3280	0,7317	0,2371	0,3197	0,5245	0,3949
AKR1B1	Aldose reductase	4,59	4,82	4,78	1,70	1,54	0,90	0,2383	0,2937	0,6160	0,2853	0,3972	0,5340

PSMC5	26S protease regulatory subunit 8	4,77	4,80	4,73	1,07	0,91	0,85	0,4673	0,4679	0,2390	0,3631	0,4546	0,3949
SEC23A	Protein transport protein Sec23A	4,19	4,19	4,31	1,00	1,32	1,33	0,9626	0,2714	0,2407	0,4884	0,3858	0,3949
STAT1	Signal transducer and activator of transcription 1-alpha/beta;Signal trans	3,86	3,67	4,26	0,64	2,47	3,86	0,2867	0,3798	0,2419	0,3093	0,4223	0,3949
PSMC1	26S protease regulatory subunit 4	4,64	4,63	4,51	0,98	0,75	0,76	0,8879	0,2421	0,3065	0,4703	0,3640	0,4247
ARPC2	Actin-related protein 2/3 complex subunit 2	4,68	4,78	4,73	1,25	1,13	0,90	0,2472	0,2646	0,5247	0,2872	0,3808	0,4886
SLC25A11	Mitochondrial 2-oxoglutarate/malate carrier protein	4,54	4,77	4,75	1,69	1,63	0,97	0,2480	0,3375	0,8963	0,2872	0,4142	0,6445
HNRNPH1	Heterogeneous nuclear ribonucleoprotein H;Heterogeneous nuclear ribo	5,39	5,38	5,34	0,98	0,89	0,91	0,8587	0,2481	0,4596	0,4606	0,3691	0,4681
ABHD10	Mycophenolic acid acyl-glucuronide esterase, mitochondrial	4,28	4,23	4,13	0,89	0,72	0,81	0,7093	0,2483	0,3897	0,4240	0,3691	0,4472
SRSF1	Serine/arginine-rich splicing factor 1	4,69	4,91	4,92	1,65	1,68	1,02	0,2822	0,2510	0,9084	0,3074	0,3705	0,6468
PSMD11	26S proteasome non-ATPase regulatory subunit 11	4,96	5,02	4,99	1,13	1,07	0,94	0,3733	0,6130	0,2517	0,3328	0,4995	0,4040
LONP1	Lon protease homolog, mitochondrial	4,42	4,32	4,44	0,79	1,06	1,34	0,2528	0,9018	0,4907	0,2884	0,5472	0,4752
TRAP1	Heat shock protein 75 kDa, mitochondrial	4,46	4,35	4,42	0,77	0,91	1,19	0,2531	0,5147	0,4977	0,2884	0,4680	0,4781
FDPS	Farnesyl pyrophosphate synthase	4,71	4,91	4,74	1,58	1,08	0,69	0,2598	0,8848	0,4339	0,2951	0,5472	0,4621
TOMM40	Mitochondrial import receptor subunit TOM40 homolog	4,39	4,26	4,40	0,74	1,02	1,37	0,2612	0,9526	0,3364	0,2951	0,5609	0,4350
SUCLG1	Succinyl-CoA ligase [ADP/GDP-forming] subunit alpha, mitochondrial	4,06	4,03	4,11	0,92	1,10	1,20	0,6331	0,6367	0,2648	0,3993	0,5050	0,4135
ATP6V1A	V-type proton ATPase catalytic subunit A	4,94	4,99	4,34	1,10	0,25	0,22	0,7141	0,2945	0,2649	0,4254	0,3972	0,4135
ATP5C1	ATP synthase subunit gamma, mitochondrial	4,74	4,77	4,82	1,08	1,22	1,13	0,5576	0,2652	0,3008	0,3838	0,3808	0,4247
ACAT2	Acetyl-CoA acetyltransferase, cytosolic	4,92	4,99	4,57	1,18	0,45	0,38	0,5823	0,3282	0,2669	0,3926	0,4137	0,4135
API5	Apoptosis inhibitor 5	4,45	4,58	4,47	1,34	1,04	0,77	0,2687	0,8925	0,4246	0,3015	0,5472	0,4615
EPHX1	Epoxide hydrolase 1	4,75	4,48	4,60	0,54	0,72	1,33	0,2718	0,3452	0,4904	0,3031	0,4142	0,4752
DCTN1	Dynactin subunit 1	4,80	4,79	4,61	0,97	0,65	0,66	0,8952	0,2842	0,2737	0,4703	0,3914	0,4183
MTA1	Metastasis-associated protein MTA1	3,66	3,70	3,82	1,08	1,44	1,33	0,8485	0,3872	0,2755	0,4582	0,4238	0,4183
NDUFS2	NADH dehydrogenase [ubiquinone] iron-sulfur protein 2, mitochondrial	4,45	4,35	4,46	0,80	1,02	1,28	0,5603	0,9565	0,2768	0,3838	0,5612	0,4183
RPL27A	60S ribosomal protein L27a	4,28	4,46	4,39	1,50	1,27	0,85	0,2792	0,2827	0,6403	0,3074	0,3914	0,5450
ILF2	Interleukin enhancer-binding factor 2	5,34	5,45	5,37	1,30	1,08	0,83	0,2934	0,7643	0,2825	0,3115	0,5290	0,4224
PCBP2	Poly(rC)-binding protein 2	4,95	5,08	4,98	1,33	1,07	0,80	0,2829	0,7324	0,3184	0,3074	0,5245	0,4294
COPS2	COP9 signalosome complex subunit 2	4,11	4,13	4,07	1,04	0,91	0,88	0,6630	0,3445	0,2830	0,4084	0,4142	0,4224
CCT5	T-complex protein 1 subunit epsilon	5,35	5,31	5,30	0,91	0,89	0,97	0,4422	0,2831	0,6251	0,3524	0,3914	0,5393
APEX1	DNA-(apurinic or apyrimidinic site) lyase;DNA-(apurinic or apyrimidinic si	4,61	4,79	4,67	1,50	1,15	0,77	0,2832	0,6495	0,3893	0,3074	0,5095	0,4472
HNRNPF	Heterogeneous nuclear ribonucleoprotein F;Heterogeneous nuclear ribo	4,58	4,58	4,62	1,01	1,10	1,10	0,9650	0,6283	0,2846	0,4889	0,5017	0,4235
RBMX	RNA-binding motif protein, X chromosome;RNA-binding motif protein, X	5,01	4,99	4,81	0,95	0,63	0,66	0,8006	0,2846	0,2898	0,4450	0,3914	0,4247
EIF4A1	Eukaryotic initiation factor 4A-I	5,55	5,58	5,61	1,06	1,15	1,09	0,5042	0,2858	0,4894	0,3703	0,3914	0,4752
ALYREF	THO complex subunit 4	4,50	4,67	4,52	1,49	1,05	0,70	0,2880	0,9372	0,5743	0,3098	0,5568	0,5094
PFN2	Profilin;Profilin-2	4,12	4,47	4,21	2,25	1,23	0,55	0,2927	0,7067	0,3585	0,3115	0,5201	0,4375
NONO	Non-POU domain-containing octamer-binding protein	5,22	5,29	5,24	1,15	1,04	0,90	0,3850	0,8285	0,2937	0,3345	0,5361	0,4247
KPNA3	Importin subunit alpha-4	4,06	4,19	4,12	1,35	1,16	0,85	0,2947	0,7049	0,5844	0,3115	0,5201	0,5171
SYNM	Synemin	4,63	4,02	4,17	0,24	0,35	1,42	0,2964	0,4215	0,6289	0,3116	0,4365	0,5399
HNRNPC	Heterogeneous nuclear ribonucleoproteins C1/C2	5,58	5,59	5,52	1,04	0,88	0,84	0,8315	0,4055	0,2975	0,4533	0,4337	0,4247
SSB	Lupus La protein	4,68	4,64	4,72	0,92	1,10	1,20	0,5515	0,3539	0,2991	0,3821	0,4175	0,4247
EIF3F	Eukaryotic translation initiation factor 3 subunit F	4,51	4,66	4,66	1,42	1,42	1,00	0,3123	0,3000	0,9814	0,3185	0,4021	0,6619
ANXA1	Annexin A1;Annexin	5,13	4,71	5,22	0,38	1,22	3,21	0,3063	0,8164	0,3005	0,3183	0,5333	0,4247
DDX39B	Spliceosome RNA helicase DDX39B	5,41	5,49	5,48	1,18	1,15	0,98	0,3007	0,3407	0,8418	0,3152	0,4142	0,6236
RPS25	40S ribosomal protein S25	4,59	4,47	4,33	0,76	0,55	0,73	0,7070	0,3012	0,5463	0,4240	0,4024	0,4987
NXN	Nucleoredoxin	4,16	4,25	4,04	1,24	0,76	0,61	0,5457	0,3475	0,3014	0,3806	0,4150	0,4247
PSMC2	26S protease regulatory subunit 7	4,63	4,71	4,57	1,21	0,88	0,73	0,3784	0,6289	0,3036	0,3328	0,5017	0,4247
PCBP1	Poly(rC)-binding protein 1	4,67	4,75	4,53	1,19	0,71	0,60	0,4207	0,5095	0,3043	0,3481	0,4675	0,4247
RRAGA;RRAGB	Ras-related GTP-binding protein A;Ras-related GTP-binding protein B	3,65	3,63	3,72	0,96	1,19	1,24	0,9128	0,6833	0,3049	0,4741	0,5187	0,4247
PCNA	Proliferating cell nuclear antigen	4,21	4,18	4,26	0,93	1,10	1,19	0,7650	0,6539	0,3059	0,4346	0,5104	0,4247
CNP	2,3-cyclic-nucleotide 3-phosphodiesterase	4,57	4,54	4,31	0,93	0,55	0,59	0,8551	0,3068	0,4066	0,4593	0,4074	0,4542
MAT2A	S-adenosylmethionine synthase isoform type-2	4,48	4,48	4,56	1,00	1,19	1,19	0,9910	0,3077	0,5262	0,4945	0,4074	0,4886
TMPO	Lamina-associated polypeptide 2, isoforms beta/gamma;Thymopoietin;T	4,91	4,96	4,84	1,12	0,84	0,75	0,6702	0,5433	0,3098	0,4104	0,4701	0,4253
CCT3	T-complex protein 1 subunit gamma	5,41	5,42	5,39	1,01	0,95	0,94	0,9315	0,6416	0,3100	0,4786	0,5066	0,4253
ERP44	Endoplasmic reticulum resident protein 44	4,33	4,24	4,24	0,81	0,83	1,01	0,3111	0,3110	0,8448	0,3185	0,4105	0,6239
EIF4A3	Eukaryotic initiation factor 4A-III;Eukaryotic initiation factor 4A-III, N-terr	4,80	4,84	4,86	1,09	1,14	1,04	0,5614	0,3124	0,7087	0,3839	0,4110	0,5715
PSME1	Proteasome activator complex subunit 1	4,27	4,41	4,48	1,38	1,64	1,19	0,4007	0,3140	0,7377	0,3425	0,4115	0,5843
MAP1LC3B;MAP1LC3B	Microtubule-associated proteins 1A/1B light chain 3B;Microtubule-assoc	4,19	3,92	4,11	0,54	0,83	1,54	0,3141	0,7206	0,5746	0,3185	0,5213	0,5094
USO1	General vesicular transport factor p115	4,15	4,04	4,15	0,77	1,00	1,29	0,4089	0,9980	0,3164	0,3458	0,5744	0,4294

VDAC1	Voltage-dependent anion-selective channel protein 1	5,47	5,43	5,46	0,91	0,98	1,08	0,3172	0,8353	0,5695	0,3185	0,5382	0,5081
CNN3	Calponin-3	4,89	4,81	4,84	0,82	0,89	1,09	0,3180	0,5498	0,7004	0,3185	0,4701	0,5695
GML;TXNRD1	Thioredoxin reductase 1, cytoplasmic	4,55	4,62	4,49	1,18	0,89	0,75	0,4843	0,6936	0,3184	0,3631	0,5201	0,4294
EEF1B2	Elongation factor 1-beta	4,40	4,69	4,69	1,94	1,96	1,01	0,3190	0,3200	0,9552	0,3185	0,4137	0,6568
ILF3	Interleukin enhancer-binding factor 3	5,24	5,20	5,16	0,92	0,83	0,90	0,5873	0,3191	0,4356	0,3926	0,4137	0,4621
LARS	Leucine--tRNA ligase, cytoplasmic	4,58	4,54	4,63	0,92	1,12	1,21	0,7285	0,6932	0,3191	0,4291	0,5201	0,4294
RCC1	Regulator of chromosome condensation	3,92	4,06	3,99	1,38	1,20	0,87	0,3204	0,4872	0,6997	0,3185	0,4608	0,5695
CANX	Calnexin	5,65	5,55	5,63	0,79	0,95	1,21	0,3219	0,7892	0,4242	0,3185	0,5305	0,4615
NOP56	Nucleolar protein 56	4,43	4,34	4,45	0,82	1,06	1,29	0,3826	0,8360	0,3223	0,3337	0,5382	0,4314
PRPF8	Pre-mRNA-processing-splicing factor 8	4,83	4,76	4,82	0,86	0,99	1,14	0,6549	0,9703	0,3224	0,4060	0,5645	0,4314
EIF3S3;EIF3H	Eukaryotic translation initiation factor 3 subunit H	3,92	3,95	3,97	1,07	1,13	1,06	0,5225	0,3234	0,4418	0,3756	0,4137	0,4665
RBM39	RNA-binding protein 39	3,98	4,17	4,22	1,56	1,74	1,11	0,4489	0,3243	0,7474	0,3562	0,4137	0,5860
CTSD	Cathepsin D;Cathepsin D light chain;Cathepsin D heavy chain	5,12	4,99	5,08	0,75	0,92	1,23	0,3248	0,3954	0,4827	0,3185	0,4305	0,4752
ALDH6A1	Methylmalonate-semialdehyde dehydrogenase [acylating], mitochondria	4,34	4,44	4,26	1,26	0,83	0,66	0,3286	0,7021	0,4471	0,3197	0,5201	0,4665
STRAP	Serine-threonine kinase receptor-associated protein	4,57	4,64	4,65	1,19	1,21	1,02	0,3586	0,3298	0,7699	0,3262	0,4137	0,5945
PYGB	Glycogen phosphorylase, brain form	5,15	5,16	5,08	1,03	0,86	0,83	0,8163	0,4800	0,3304	0,4490	0,4608	0,4345
PRCP	Lysosomal Pro-X carboxypeptidase	4,74	4,66	4,61	0,84	0,74	0,88	0,6524	0,3306	0,7790	0,4056	0,4137	0,5997
TXNL1	Thioredoxin-like protein 1	4,16	4,21	4,09	1,12	0,85	0,76	0,6117	0,4710	0,3308	0,3960	0,4555	0,4345
RUVBL2	RuvB-like 2	4,72	4,76	4,78	1,09	1,14	1,05	0,6039	0,3321	0,7866	0,3926	0,4138	0,6027
IDH1	Isocitrate dehydrogenase [NADP] cytoplasmic	5,37	5,43	5,38	1,15	1,02	0,88	0,3352	0,9655	0,7698	0,3221	0,5641	0,5945
HNRNPR	Heterogeneous nuclear ribonucleoprotein R	5,40	5,39	5,34	0,98	0,87	0,89	0,8924	0,4541	0,3352	0,4703	0,4491	0,4350
CLU	Clusterin;Clusterin beta chain;Clusterin alpha chain;Clusterin	5,44	5,22	5,40	0,61	0,93	1,52	0,3365	0,8170	0,3626	0,3221	0,5333	0,4375
MTHFD1	C-1-tetrahydrofolate synthase, cytoplasmic;Methylenetetrahydrofolate d	4,34	4,52	4,39	1,52	1,13	0,74	0,3390	0,5617	0,4623	0,3230	0,4712	0,4681
USP14	Ubiquitin carboxyl-terminal hydrolase;Ubiquitin carboxyl-terminal hydro	4,58	4,51	4,60	0,85	1,05	1,24	0,4646	0,5549	0,3416	0,3631	0,4701	0,4358
DYNC1LI2	Cytoplasmic dynein 1 light intermediate chain 2	4,60	4,63	4,42	1,07	0,67	0,62	0,8305	0,3985	0,3435	0,4533	0,4318	0,4358
RAP1B;RAP1A	Ras-related protein Rap-1b;Ras-related protein Rap-1b-like protein;Ras-r	4,41	4,42	4,52	1,02	1,28	1,26	0,8711	0,3511	0,3438	0,4652	0,4156	0,4358
HP1BP3	Heterochromatin protein 1-binding protein 3	5,02	5,08	5,03	1,14	1,02	0,89	0,4309	0,9342	0,3450	0,3505	0,5568	0,4358
COPS6	COP9 signalosome complex subunit 6	4,16	4,27	4,22	1,30	1,14	0,88	0,3450	0,5850	0,4610	0,3239	0,4845	0,4681
SLC25A4	ADP/ATP translocase 1	4,43	4,55	4,44	1,31	1,00	0,77	0,3495	0,9885	0,3850	0,3239	0,5713	0,4454
TALDO1	Transaldolase	4,54	4,65	4,55	1,27	1,01	0,80	0,3500	0,9534	0,4566	0,3239	0,5609	0,4681
EIF2S3;EIF2S3L	Eukaryotic translation initiation factor 2 subunit 3;Putative eukaryotic tra	4,29	4,22	4,31	0,84	1,05	1,25	0,3757	0,7421	0,3509	0,3328	0,5259	0,4375
SORBS2	Sorbin and SH3 domain-containing protein 2	4,40	4,45	3,94	1,11	0,34	0,31	0,9125	0,3512	0,3584	0,4741	0,4156	0,4375
IPO5	Importin-5	5,22	5,20	5,17	0,97	0,89	0,92	0,8137	0,4026	0,3525	0,4483	0,4328	0,4375
MSN	Moesin	5,43	5,33	5,37	0,80	0,87	1,10	0,3543	0,7465	0,8392	0,3248	0,5268	0,6234
SLC3A2	4F2 cell-surface antigen heavy chain	4,80	4,64	4,65	0,69	0,71	1,02	0,3570	0,3602	0,9406	0,3262	0,4200	0,6561
PSMD2	26S proteasome non-ATPase regulatory subunit 2	4,81	4,83	4,77	1,05	0,92	0,88	0,6532	0,5487	0,3578	0,4056	0,4701	0,4375
KPNA4	Importin subunit alpha-3	4,28	4,35	4,30	1,17	1,05	0,89	0,3814	0,8171	0,3603	0,3337	0,5333	0,4375
RPLP0;RPLP0P6	60S acidic ribosomal protein P0;60S acidic ribosomal protein P0-like	4,96	4,96	5,04	1,00	1,20	1,20	0,9804	0,3935	0,3629	0,4917	0,4296	0,4375
GART	Trifunctional purine biosynthetic protein adenosine-3;Phosphoribosylam	4,41	4,44	4,47	1,06	1,13	1,07	0,6948	0,3660	0,5017	0,4187	0,4200	0,4794
HSPH1	Heat shock protein 105 kDa	4,71	4,68	4,62	0,93	0,81	0,87	0,7444	0,3670	0,5139	0,4295	0,4200	0,4817
ATXN10	Ataxin-10	4,30	4,39	4,34	1,21	1,08	0,89	0,3681	0,7211	0,4001	0,3314	0,5213	0,4487
RAP1GDS1	Rap1 GTPase-GDP dissociation stimulator 1	4,40	4,27	4,24	0,74	0,70	0,95	0,3688	0,3866	0,8712	0,3314	0,4238	0,6377
PSMD1	26S proteasome non-ATPase regulatory subunit 1	4,90	4,93	4,98	1,08	1,19	1,10	0,6008	0,3722	0,5687	0,3926	0,4204	0,5081
HMGB1	High mobility group protein B1	4,95	5,16	5,00	1,61	1,13	0,70	0,3724	0,7953	0,4712	0,3328	0,5305	0,4723
HSP90AA1	Heat shock protein HSP 90-alpha	6,09	6,14	6,08	1,12	0,97	0,87	0,3725	0,8704	0,4144	0,3328	0,5453	0,4564
TBCD	Tubulin-specific chaperone D	4,44	4,36	4,46	0,82	1,04	1,27	0,4406	0,8944	0,3728	0,3524	0,5472	0,4438
IGF2BP3	Insulin-like growth factor 2 mRNA-binding protein 3	4,72	4,62	4,83	0,81	1,31	1,62	0,5398	0,6507	0,3739	0,3797	0,5095	0,4438
EIF3A	Eukaryotic translation initiation factor 3 subunit A	4,47	4,34	4,42	0,74	0,89	1,20	0,4820	0,7693	0,3755	0,3631	0,5305	0,4438
CBR1	Carbonyl reductase [NADPH] 1	4,30	4,41	4,32	1,28	1,03	0,81	0,3764	0,9102	0,5505	0,3328	0,5500	0,4987
CCT7	T-complex protein 1 subunit eta	5,15	5,11	5,11	0,90	0,90	1,00	0,4161	0,3774	0,9302	0,3481	0,4222	0,6545
EZR	Ezrin	4,85	5,05	4,25	1,59	0,25	0,16	0,4189	0,5069	0,3777	0,3481	0,4675	0,4438
TROVE2	60 kDa SS-A/Ro ribonucleoprotein	4,50	4,62	4,70	1,32	1,58	1,20	0,4731	0,3798	0,6450	0,3631	0,4223	0,5471
IPO9	Importin-9	4,56	4,61	4,51	1,13	0,90	0,79	0,7093	0,7074	0,3803	0,4240	0,5201	0,4454
HNRNPL	Heterogeneous nuclear ribonucleoprotein L	5,23	5,32	5,28	1,25	1,14	0,91	0,4354	0,6547	0,3814	0,3524	0,5104	0,4454
FUBP1	Far upstream element-binding protein 1	4,72	4,83	4,63	1,30	0,82	0,63	0,6192	0,7595	0,3827	0,3979	0,5288	0,4454
SSRP1	FACT complex subunit SSRP1	4,49	4,53	4,41	1,10	0,83	0,76	0,8372	0,7052	0,3848	0,4557	0,5201	0,4454
PTBP1	Polypyrimidine tract-binding protein 1	4,98	4,98	5,07	1,00	1,23	1,22	0,9857	0,4879	0,3862	0,4932	0,4608	0,4454

RPL7A	60S ribosomal protein L7a	4,90	4,89	4,96	0,96	1,14	1,19	0,8613	0,6226	0,3947	0,4613	0,5017	0,4479
GRHPR	Glyoxylate reductase/hydroxypyruvate reductase	4,44	4,40	4,36	0,91	0,83	0,92	0,6693	0,4541	0,3958	0,4104	0,4491	0,4479
EMD	Emerin	3,95	3,84	4,00	0,79	1,14	1,44	0,5883	0,4939	0,3968	0,3926	0,4608	0,4480
ARPC5	Actin-related protein 2/3 complex subunit 5	3,94	3,98	3,92	1,10	0,95	0,86	0,6264	0,7192	0,3998	0,3982	0,5213	0,4487
CTSB	Cathepsin B;Cathepsin B light chain;Cathepsin B heavy chain	4,46	4,49	4,57	1,07	1,28	1,20	0,8089	0,4005	0,6260	0,4470	0,4318	0,5393
PSMB5	Proteasome subunit beta type-5	4,03	4,24	4,07	1,62	1,11	0,68	0,4053	0,4373	0,5101	0,3448	0,4421	0,4794
ECHS1	Enoyl-CoA hydratase, mitochondrial	4,62	4,63	4,72	1,04	1,27	1,23	0,6811	0,4069	0,4594	0,4146	0,4341	0,4681
FARSA	Phenylalanine--tRNA ligase alpha subunit	4,19	4,20	4,14	1,02	0,90	0,88	0,8403	0,4213	0,4085	0,4567	0,4365	0,4542
CAP1	Adenylyl cyclase-associated protein 1;Adenylyl cyclase-associated protei	5,05	5,02	4,88	0,93	0,67	0,72	0,4780	0,4098	0,4915	0,3631	0,4346	0,4752
RALY	RNA-binding protein Raly	4,71	4,81	4,69	1,23	0,94	0,76	0,4945	0,7593	0,4106	0,3669	0,5288	0,4542
SET;SETSIP	Protein SET;Protein SETSIP	4,81	4,92	4,84	1,28	1,08	0,84	0,4108	0,8668	0,6824	0,3463	0,5453	0,5642
GTF2I	General transcription factor II-I	4,39	4,35	4,41	0,91	1,04	1,15	0,6710	0,7826	0,4125	0,4104	0,5305	0,4553
PPP1CB	Serine/threonine-protein phosphatase PP1-beta catalytic subunit;Serine	4,75	4,79	4,77	1,10	1,06	0,96	0,4151	0,4520	0,7134	0,3481	0,4491	0,5734
PRKAR2A	cAMP-dependent protein kinase type II-alpha regulatory subunit	4,16	4,08	4,08	0,83	0,83	1,00	0,4402	0,4154	0,9925	0,3524	0,4365	0,6632
XRCC6	X-ray repair cross-complementing protein 6	5,50	5,58	5,56	1,19	1,13	0,95	0,4298	0,4169	0,7603	0,3505	0,4365	0,5945
ARCN1	Coatomer subunit delta	4,19	4,24	4,26	1,11	1,17	1,05	0,5224	0,4194	0,6505	0,3756	0,4365	0,5480
RBM14	RNA-binding protein 14	4,18	4,15	4,10	0,92	0,82	0,89	0,6858	0,4207	0,6502	0,4153	0,4365	0,5480
ADH5	Alcohol dehydrogenase class-3	4,69	4,77	4,69	1,21	1,01	0,84	0,4624	0,9350	0,4227	0,3629	0,5568	0,4615
MYH3	Myosin-3	2,71	2,44	3,92	0,53	16,31	30,84	0,5374	0,5174	0,4243	0,3795	0,4680	0,4615
GSR	Glutathione reductase, mitochondrial	3,87	3,92	3,70	1,11	0,66	0,60	0,5008	0,5136	0,4270	0,3701	0,4680	0,4615
CTNNA1	Catenin alpha-1	4,63	4,58	4,44	0,89	0,65	0,73	0,8440	0,4883	0,4276	0,4581	0,4608	0,4615
TRIM28	Transcription intermediary factor 1-beta	5,24	5,29	5,24	1,14	1,02	0,89	0,6312	0,9368	0,4304	0,3993	0,5568	0,4615
HDAC2	Histone deacetylase 2	4,47	4,58	4,59	1,30	1,31	1,01	0,4594	0,4304	0,9681	0,3614	0,4405	0,6595
NAP1L1	Nucleosome assembly protein 1-like 1	5,00	4,98	4,91	0,95	0,81	0,85	0,7663	0,4336	0,5438	0,4346	0,4405	0,4983
TUBA1A;TUBA3C;TUBA	Tubulin alpha-1A chain;Tubulin alpha-3C/D chain;Tubulin alpha-3E chain	5,83	5,91	5,79	1,20	0,93	0,77	0,8308	0,9300	0,4338	0,4533	0,5568	0,4621
SNRNP70	U1 small nuclear ribonucleoprotein 70 kDa	4,54	4,66	4,57	1,34	1,07	0,80	0,4372	0,8439	0,4352	0,3524	0,5393	0,4621
PSAT1	Phosphoserine aminotransferase	5,15	5,22	5,31	1,17	1,46	1,25	0,6783	0,4354	0,4690	0,4135	0,4412	0,4710
PDCD6IP	Programmed cell death 6-interacting protein	4,15	4,19	4,18	1,10	1,07	0,97	0,4359	0,7941	0,9070	0,3524	0,5305	0,6468
ALDH9A1	4-trimethylaminobutyraldehyde dehydrogenase	4,59	4,62	4,69	1,07	1,26	1,18	0,7235	0,4390	0,5621	0,4289	0,4427	0,5056
XRCC5	X-ray repair cross-complementing protein 5	5,48	5,55	5,53	1,17	1,12	0,95	0,4413	0,5333	0,6636	0,3524	0,4701	0,5533
CNDP2	Cytosolic non-specific dipeptidase	4,80	4,84	4,54	1,09	0,55	0,50	0,6014	0,5046	0,4473	0,3926	0,4667	0,4665
SMARCA5	SWI/SNF-related matrix-associated actin-dependent regulator of chroma	4,20	4,15	4,10	0,89	0,81	0,91	0,7281	0,4499	0,7624	0,4291	0,4491	0,5945
TTN	Titin	3,12	3,46	3,95	2,18	6,79	3,11	0,4520	0,5161	0,6993	0,3578	0,4680	0,5695
NUMA1	Nuclear mitotic apparatus protein 1	4,39	4,65	4,74	1,84	2,26	1,23	0,5829	0,4564	0,5295	0,3926	0,4491	0,4906
DNM1L	Dynamin-1-like protein	4,57	4,53	4,45	0,91	0,77	0,84	0,8180	0,4583	0,4907	0,4493	0,4491	0,4752
NCL	Nucleolin	5,41	5,33	5,38	0,83	0,94	1,13	0,4695	0,8243	0,4600	0,3631	0,5351	0,4681
LLGL1	Lethal(2) giant larvae protein homolog 1	4,51	4,38	4,53	0,75	1,06	1,41	0,4654	0,8996	0,4604	0,3631	0,5472	0,4681
AGRN	Agrin;Agrin N-terminal 110 kDa subunit;Agrin C-terminal 110 kDa subuni	4,05	4,03	4,08	0,96	1,08	1,12	0,8833	0,7833	0,4661	0,4697	0,5305	0,4699
CAMK1D;CAMK1	Calcium/calmodulin-dependent protein kinase type 1D;Calcium/calmodu	3,52	3,63	3,60	1,30	1,20	0,92	0,4664	0,7816	0,8875	0,3631	0,5305	0,6445
UBA1	Ubiquitin-like modifier-activating enzyme 1	5,79	5,79	5,70	1,00	0,83	0,83	0,9991	0,5423	0,4669	0,4969	0,4701	0,4699
DHX9	ATP-dependent RNA helicase A	5,59	5,55	5,54	0,91	0,89	0,98	0,5355	0,4670	0,8131	0,3789	0,4546	0,6144
RPL22	60S ribosomal protein L22	4,21	4,32	4,33	1,28	1,32	1,03	0,4756	0,4889	0,9068	0,3631	0,4608	0,6468
KPNB1	Importin subunit beta-1	5,41	5,44	5,44	1,08	1,08	1,00	0,5124	0,4768	0,9681	0,3726	0,4594	0,6595
NAP1L4	Nucleosome assembly protein 1-like 4	4,87	4,90	4,86	1,06	0,97	0,92	0,6405	0,6804	0,4769	0,4025	0,5174	0,4746
PA2G4	Proliferation-associated protein 2G4	4,59	4,66	4,61	1,18	1,05	0,89	0,4772	0,7104	0,6041	0,3631	0,5206	0,5280
BZW2	Basic leucine zipper and W2 domain-containing protein 2	4,11	4,16	4,16	1,14	1,13	1,00	0,4841	0,5182	0,9879	0,3631	0,4680	0,6619
HSP90AB2P	Putative heat shock protein HSP 90-beta 2	4,59	4,67	4,69	1,20	1,24	1,04	0,5027	0,4841	0,8098	0,3703	0,4608	0,6138
TARDBP	TAR DNA-binding protein 43	4,35	4,33	4,34	0,95	0,98	1,04	0,4864	0,8463	0,7665	0,3631	0,5393	0,5945
ACTR3	Actin-related protein 3	5,04	5,05	5,03	1,02	0,96	0,94	0,8032	0,7152	0,4868	0,4458	0,5213	0,4752
HNRNPUL2;HNRNPUL2	Heterogeneous nuclear ribonucleoprotein U-like protein 2	4,54	4,57	4,59	1,09	1,14	1,04	0,6614	0,4897	0,6981	0,4084	0,4608	0,5695
OAT	Ornithine aminotransferase, mitochondrial;Ornithine aminotransferase,	4,21	4,16	4,07	0,88	0,72	0,82	0,6948	0,4931	0,5578	0,4187	0,4608	0,5026
OSBPL8	Oxysterol-binding protein-related protein 8;Oxysterol-binding protein	3,75	3,69	3,71	0,87	0,91	1,05	0,4936	0,6502	0,8158	0,3669	0,5095	0,6150
SNRNP200	U5 small nuclear ribonucleoprotein 200 kDa helicase	4,82	4,73	4,79	0,81	0,93	1,16	0,5670	0,8599	0,4946	0,3862	0,5438	0,4772
RBBP4	Histone-binding protein RBBP4	4,69	4,77	4,74	1,21	1,13	0,93	0,5038	0,7040	0,8323	0,3703	0,5201	0,6203
EEF1G	Elongation factor 1-gamma	5,29	5,29	5,31	0,99	1,04	1,05	0,8925	0,5757	0,5041	0,4703	0,4800	0,4794
PGD	6-phosphogluconate dehydrogenase, decarboxylating	5,59	5,58	5,57	0,98	0,94	0,96	0,7980	0,5856	0,5051	0,4446	0,4845	0,4794
EIF3L	Eukaryotic translation initiation factor 3 subunit L	4,30	4,35	4,33	1,13	1,09	0,96	0,5059	0,6606	0,8002	0,3708	0,5107	0,6098

PSPC1	Paraspeckle component 1	4,44	4,39	4,37	0,90	0,85	0,94	0,7878	0,5078	0,8960	0,4406	0,4675	0,6445
PRMT1	Protein arginine N-methyltransferase 1	4,55	4,50	4,56	0,90	1,02	1,13	0,5288	0,8851	0,5086	0,3778	0,5472	0,4794
KHSRP	Far upstream element-binding protein 2	4,87	4,92	4,89	1,13	1,04	0,92	0,6209	0,8543	0,5105	0,3979	0,5417	0,4794
CRABP1	Cellular retinoic acid-binding protein 1	4,68	4,54	4,46	0,71	0,59	0,83	0,6330	0,5215	0,7059	0,3993	0,4684	0,5714
FHL1	Four and a half LIM domains protein 1	4,10	4,13	4,22	1,09	1,34	1,23	0,7584	0,5219	0,6156	0,4335	0,4684	0,5340
RPSA;RPSAP58	40S ribosomal protein SA	5,12	5,06	5,10	0,87	0,95	1,09	0,5227	0,8294	0,7467	0,3756	0,5361	0,5860
NASP	Nuclear autoantigenic sperm protein	4,32	4,27	4,47	0,88	1,43	1,61	0,8880	0,5301	0,5728	0,4703	0,4701	0,5094
IDH2	Isocitrate dehydrogenase [NADP], mitochondrial	5,55	5,55	5,59	1,00	1,09	1,09	0,9803	0,5350	0,5304	0,4917	0,4701	0,4906
PRPF19	Pre-mRNA-processing factor 19	4,73	4,74	4,76	1,04	1,10	1,05	0,7393	0,5341	0,7026	0,4293	0,4701	0,5703
SUPT16H	FACT complex subunit SPT16	4,44	4,23	4,35	0,61	0,80	1,30	0,5342	0,7951	0,5910	0,3789	0,5305	0,5210
NPEPPS	Puromycin-sensitive aminopeptidase	5,29	5,31	5,28	1,06	0,98	0,92	0,7864	0,8966	0,5428	0,4406	0,5472	0,4983
SDHA	Succinate dehydrogenase [ubiquinone] flavoprotein subunit, mitochondr	4,27	4,30	4,30	1,09	1,09	1,00	0,6400	0,5464	0,9902	0,4025	0,4701	0,6626
LMNB2	Lamin-B2	4,92	4,94	4,95	1,05	1,07	1,02	0,5472	0,5927	0,8670	0,3806	0,4894	0,6365
CCT8	T-complex protein 1 subunit theta	5,40	5,43	5,41	1,06	1,02	0,96	0,6033	0,8614	0,5497	0,3926	0,5438	0,4987
RPS6	40S ribosomal protein S6	4,59	4,62	4,67	1,08	1,21	1,12	0,7332	0,5508	0,6682	0,4291	0,4701	0,5558
KIF5C	Kinesin heavy chain isoform 5C	5,08	5,01	4,98	0,86	0,80	0,92	0,5510	0,6598	0,8868	0,3821	0,5107	0,6445
PSMD13	26S proteasome non-ATPase regulatory subunit 13	4,72	4,64	4,73	0,82	1,03	1,25	0,6028	0,8984	0,5513	0,3926	0,5472	0,4987
HUWE1	E3 ubiquitin-protein ligase HUWE1	4,25	4,18	4,15	0,85	0,79	0,93	0,6944	0,5826	0,5524	0,4187	0,4839	0,4987
EIF5	Eukaryotic translation initiation factor 5	4,15	4,09	4,10	0,88	0,89	1,01	0,6454	0,5541	0,9786	0,4050	0,4701	0,6619
EIF4A2	Eukaryotic initiation factor 4A-II	4,68	4,65	4,63	0,92	0,89	0,97	0,6642	0,5597	0,7420	0,4084	0,4712	0,5860
VARS	Valine--tRNA ligase	4,47	4,42	4,46	0,90	0,98	1,10	0,6043	0,9298	0,5700	0,3926	0,5568	0,5081
TPP1	Tripeptidyl-peptidase 1	4,74	4,65	4,67	0,82	0,86	1,04	0,5719	0,7348	0,9393	0,3889	0,5249	0,6561
PSMD6	26S proteasome non-ATPase regulatory subunit 6	4,54	4,55	4,50	1,01	0,91	0,90	0,9709	0,5748	0,6461	0,4912	0,4800	0,5471
PSMC4	26S protease regulatory subunit 6B	4,63	4,62	4,54	0,98	0,80	0,82	0,9476	0,6278	0,5919	0,4834	0,5017	0,5210
PSMD3	26S proteasome non-ATPase regulatory subunit 3	4,84	4,82	4,80	0,96	0,91	0,95	0,5949	0,6694	0,8129	0,3926	0,5137	0,6144
SHMT2	Serine hydroxymethyltransferase, mitochondrial;Serine hydroxymethyltr	4,76	4,70	4,80	0,87	1,10	1,27	0,6015	0,8389	0,6165	0,3926	0,5383	0,5340
AP1G1	AP-1 complex subunit gamma-1	4,35	4,40	4,34	1,13	0,96	0,85	0,7379	0,9247	0,6037	0,4291	0,5568	0,5280
NACA	Nascent polypeptide-associated complex subunit alpha;Nascent polypep	4,77	4,84	4,84	1,18	1,17	0,99	0,6098	0,6122	0,9494	0,3955	0,4995	0,6568
GLUD1;GLUD2	Glutamate dehydrogenase 1, mitochondrial;Glutamate dehydrogenase 2	5,15	5,10	5,08	0,89	0,85	0,96	0,6246	0,6149	0,9113	0,3982	0,4995	0,6468
PARP1	Poly [ADP-ribose] polymerase 1	5,26	5,30	5,29	1,11	1,07	0,97	0,6178	0,7959	0,8948	0,3979	0,5305	0,6445
FKBP4	Peptidyl-prolyl cis-trans isomerase FKBP4;Peptidyl-prolyl cis-trans isome	4,54	4,55	4,45	1,03	0,82	0,80	0,9126	0,6493	0,6275	0,4741	0,5095	0,5397
SF3A2	Splicing factor 3A subunit 2	3,98	4,02	4,01	1,09	1,09	0,99	0,7064	0,6282	0,9657	0,4240	0,5017	0,6595
TECR	Very-long-chain enoyl-CoA reductase	4,04	4,11	4,12	1,16	1,21	1,04	0,7324	0,6394	0,8782	0,4291	0,5062	0,6418
PSMD12	26S proteasome non-ATPase regulatory subunit 12	4,72	4,74	4,74	1,03	1,03	1,00	0,6483	0,6737	0,9734	0,4054	0,5151	0,6612
CCAR2	Cell cycle and apoptosis regulator protein 2	4,31	4,29	4,25	0,97	0,87	0,90	0,9137	0,6935	0,6548	0,4741	0,5201	0,5488
HMOX2	Heme oxygenase 2	3,83	3,83	3,78	1,02	0,89	0,88	0,9273	0,6562	0,6688	0,4778	0,5104	0,5558
GGH	Gamma-glutamyl hydrolase	4,79	4,74	4,72	0,90	0,86	0,96	0,7825	0,6873	0,9014	0,4403	0,5201	0,6463
DDX1	ATP-dependent RNA helicase DDX1	4,91	4,92	4,90	1,03	0,98	0,95	0,8221	0,9028	0,6906	0,4507	0,5472	0,5681
NARS	Asparagine--tRNA ligase, cytoplasmic	4,70	4,73	4,75	1,07	1,12	1,04	0,8231	0,6937	0,8654	0,4507	0,5201	0,6363
VDAC2	Voltage-dependent anion-selective channel protein 2	5,38	5,39	5,35	1,01	0,93	0,92	0,8962	0,7488	0,6939	0,4703	0,5268	0,5690
PSMD7	26S proteasome non-ATPase regulatory subunit 7	4,39	4,37	4,37	0,93	0,94	1,00	0,7008	0,7169	0,9874	0,4217	0,5213	0,6619
CSE1L	Exportin-2	5,58	5,56	5,53	0,94	0,89	0,95	0,7852	0,7054	0,8164	0,4406	0,5201	0,6150
SYNCRIP	Heterogeneous nuclear ribonucleoprotein Q	5,19	5,18	5,18	0,99	0,98	0,99	0,8893	0,7085	0,8227	0,4703	0,5201	0,6187
TPT1	Translationally-controlled tumor protein;TPT1-like protein	4,43	4,45	4,47	1,05	1,08	1,03	0,7309	0,7149	0,8837	0,4291	0,5213	0,6445
RTCB	tRNA-splicing ligase RtcB homolog	4,92	4,94	4,94	1,06	1,04	0,98	0,7155	0,7495	0,8994	0,4256	0,5268	0,6458
HNRNPM	Heterogeneous nuclear ribonucleoprotein M	5,55	5,58	5,58	1,07	1,07	1,00	0,7295	0,7549	0,9562	0,4291	0,5288	0,6568
DEK	Protein DEK	4,37	4,36	4,33	0,98	0,92	0,93	0,9511	0,7314	0,7922	0,4843	0,5245	0,6052
VDAC3	Voltage-dependent anion-selective channel protein 3	4,89	4,86	4,89	0,92	1,00	1,08	0,7359	0,9854	0,7684	0,4291	0,5712	0,5945
BLMH	Bleomycin hydrolase	4,29	4,31	4,31	1,05	1,04	1,00	0,7367	0,7622	0,9781	0,4291	0,5288	0,6619
CSDE1	Cold shock domain-containing protein E1	3,95	3,94	3,96	0,97	1,01	1,05	0,7642	0,9498	0,8250	0,4346	0,5609	0,6188
SLC25A6	ADP/ATP translocase 3;ADP/ATP translocase 3, N-terminally processed	4,65	4,65	4,67	0,99	1,06	1,07	0,9781	0,8465	0,7661	0,4917	0,5393	0,5945
ARL6IP5	PRA1 family protein 3	4,64	4,61	4,65	0,94	1,02	1,08	0,7985	0,9396	0,7668	0,4446	0,5574	0,5945
YARS	Tyrosine--tRNA ligase, cytoplasmic;Tyrosine--tRNA ligase, cytoplasmic, N	4,34	4,36	4,35	1,05	1,02	0,97	0,7715	0,9687	0,9254	0,4368	0,5645	0,6542
SLC25A3	Phosphate carrier protein, mitochondrial	4,89	4,90	4,87	1,00	0,95	0,95	0,9777	0,7799	0,7807	0,4917	0,5305	0,6001
PRMT5	Protein arginine N-methyltransferase 5;Protein arginine N-methyltransfe	3,90	3,92	3,93	1,03	1,06	1,03	0,8659	0,7855	0,8700	0,4631	0,5305	0,6377
HNRNPU	Heterogeneous nuclear ribonucleoprotein U	5,45	5,46	5,47	1,02	1,04	1,02	0,9102	0,8234	0,8096	0,4741	0,5351	0,6138
HSDL2	Hydroxysteroid dehydrogenase-like protein 2	4,21	4,21	4,18	0,99	0,93	0,93	0,9888	0,8400	0,8297	0,4941	0,5383	0,6193

QARS	Glutamine--tRNA ligase	4,43	4,45	4,42	1,04	0,96	0,92	0,9056	0,8886	0,8493	0,4741	0,5472	0,6263
MTCH2	Mitochondrial carrier homolog 2	4,51	4,49	4,50	0,96	0,98	1,02	0,8544	0,9301	0,9511	0,4593	0,5568	0,6568
CPSF6	Cleavage and polyadenylation specificity factor subunit 6	4,28	4,30	4,30	1,04	1,05	1,01	0,8949	0,8602	0,9552	0,4703	0,5438	0,6568
BCLAF1	Bcl-2-associated transcription factor 1	4,11	4,09	4,08	0,96	0,93	0,97	0,9078	0,8849	0,9357	0,4741	0,5472	0,6561
UGP2	UTP--glucose-1-phosphate uridylyltransferase	4,46	4,45	4,46	0,98	1,00	1,02	0,9275	0,9977	0,8892	0,4778	0,5744	0,6445
KARS	Lysine--tRNA ligase	4,47	4,47	4,48	1,00	1,01	1,01	0,9999	0,9333	0,9417	0,4969	0,5568	0,6561
