# Supplementary material for: Comparative characterization of human induced pluripotent stem cells (hiPSC) derived from patients with schizophrenia and autism
Source: Transl Psychiatry. 2019 Jul 29;9:179. doi: 10.1038/s41398-019-0517-3 (PMC6663940; doi:10.1038/s41398-019-0517-3)
Supplement: Supplementary file 4 — Supplemental material [file 41398_2019_517_MOESM4_ESM.docx]

Supplemental information

Supplemental figure S1

Supplemental Figure S1

Reprogrammed fibroblasts exhibit a stem cell phenotype. (A) Human dermal fibroblasts (HDF) and (B) stem cell colonies grown after retroviral transduction of OCT3/4, SOX2, KLF4 and c-MYC. (C,D) Expression of stem cell markers TRA1-8-1 and SSEA4. Scale bar (A-C): 100 µm. (E)-(G) Assessment of pluripotency by differentiation of hiPSC into phenotypes representative for the three germ layers: (E) mesodermal phenotypes as shown by staining for cTNT (cardiac troponin T), (F) endodermal phenotypes with staining for SOX17, (G) ectodermal phenotypes as shown by β-III-tubulin staining. (E’)-(G’): Hoechst staining for cells shown in (E)-(G). Scale bar E-F: 100 µm, G: 10 µm. (H,I) Expression of stem cell marker as analysed by RT-PCR. Exogenously transduced genes OCT3/4, SOX2, KLF4 and c-MYC became silenced (H) while endogenously expressed markers (I) became upregulated. Ntera cells and HDF were taken as positive or negative controls, respectively; GAPDH was served as a loading control. (K) Depiction of potential chromosomal rearrangements as compared between individual HDF and their respective reprogrammed counterparts by chromosomal genome hybridization. No major alterations were observed.

Differentiation of hiPSC into neural progenitor cells (NPC) and neurons. (K,L) Expression of NPC marker. ZO 1=Zona Occludens 1, PAX 6=Paired box gene 6, SOX 1= SRY box 1, Scale bar: 50 µm. (N) Immunocytochemical staining against neuronal synaptic markers. PSD95= Post synaptic density protein 95, VGluT=vesicular glutamate transporter 1. Scale bar: 10µm.

Supplemental figure S2

Supplemental figure S2

Immunostaining against stem cell markers. iPSCs from all donors were stained against stem cell marker genes SSEA 4 and Tra 1-81. Scale bars 100 µm.

Supplemental figure S3

Supplemental figure S3

Immunocytochemical staining against neural progenitor cell (NPC) markers for all donors. NPCs were stained for ZO 1 and transcriptions factor PAX 6 as well as Hoechst. Scale bar 100 µm.

Supplemental figure S4

Supplemental figure S4

Immunocytochemical staining against neuronal progenitor cell (NPC) markers for all donors. NPCs were stained for Nestin and transcription factor SOX 1 as well as Hoechst. Scale bar 100 µm.


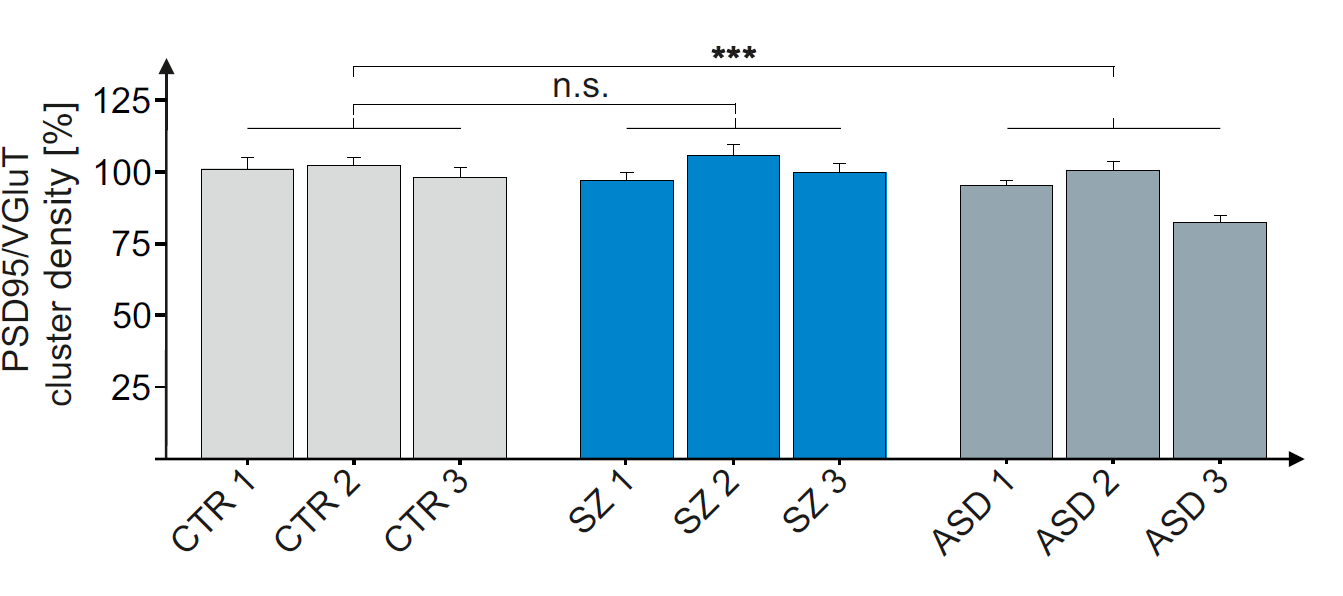


Supplemental figure S5

Quantification of synaptic structures identified by colocalized PSD95/VGlut immunostaining synaptical marker for all donors. Kruskal-Wallis Test and Dunn’s post hoc test, H(2)=24.4.; ***: p<0.001, n.s.= not significant, n=360, error bars are s.e.m.

Figure S6

Healthy control CTR1 is depicted in black while SZ2 is represented in blue. For the healthy control, a significant concentration-dependent decline in neurite outgrowth to up to 57% and 61% maximum was observed after application of up to 0.067 µM of haloperidol or 1 µM olanzapine, respectively. By contrast, clozapine increases neurite outgrowth, however, without significant alterations. None of the antipsychotics rescued impaired neurite outgrowth observed in neurons derived from a patient with schizophrenia.

Supplemental figure S7


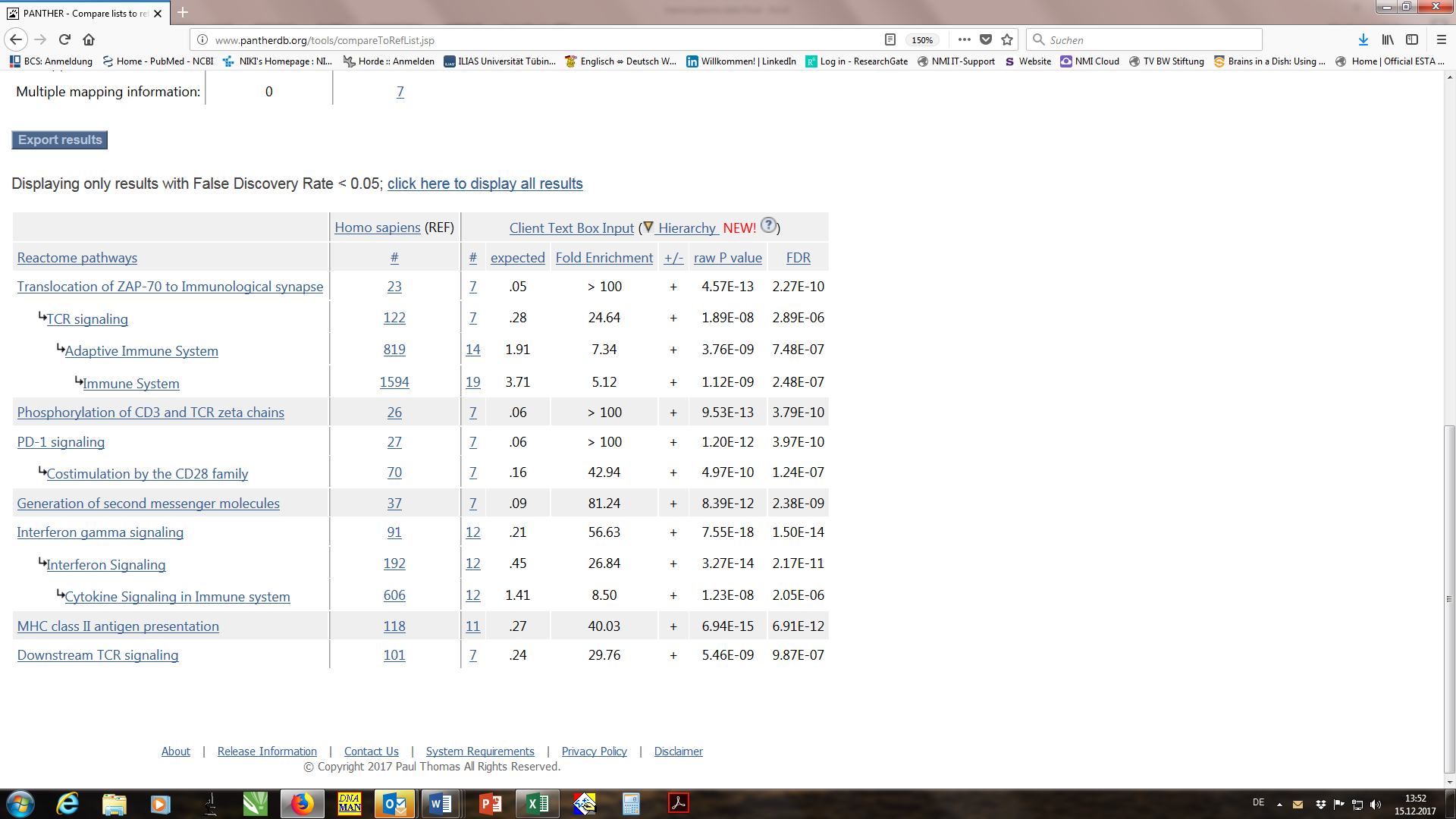


Gene ontology analysis of the reactome of deregulated genes of SZ2 ([www.pantherdb.org](http://www.pantherdb.org)) compared to the group of healthy controls

Supplemental figure S8


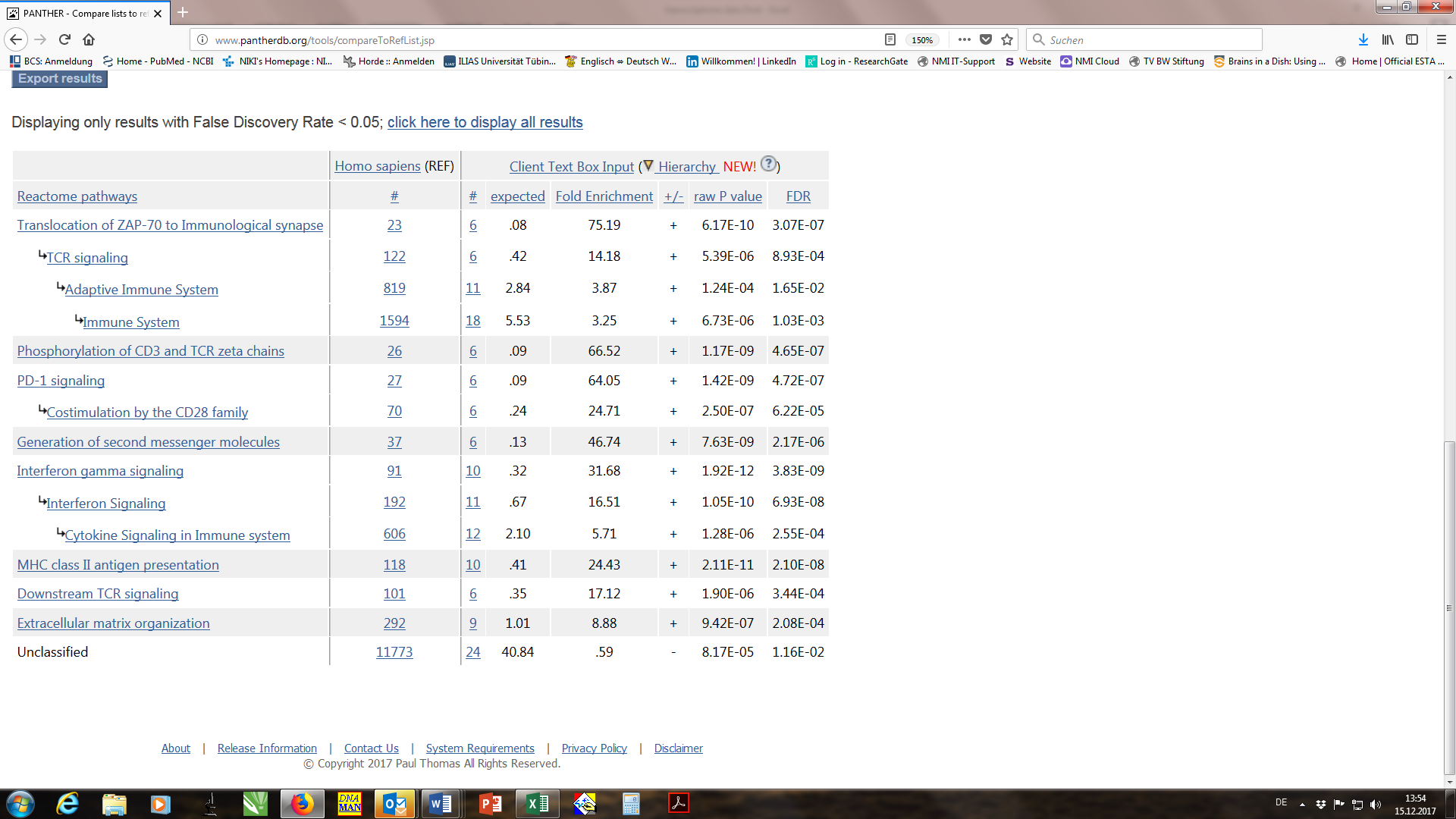


Gene ontology analysis of the reactome of deregulated genes of CTR1 after clozapine treatment compared to DMSO-treated CTR1 ([www.pantherdb.org](http://www.pantherdb.org))

Supplemental methods

Selection of patients with schizophrenia or autism

Inclusion criteria schizophrenia:

- Diagnosis of schizophrenia investigated by SCID-I according to DSM-IV
- One first-degree relative also with the diagnosis of schizophrenia
- Age: 18 – 65 years

Inclusion criteria autism:

- diagnosis of autism according to DSM-IV
- disease onset in early childhood
- Age: 18 – 65 years

Exclusion criteria schizophrenia and autism:

- Any other diagnosis according to DSM-IV
- Current addiction
- Use of illegal substances within 4 weeks before study
- Mentally handicapped persons
- Pregnancy or lactation
- Suicidal tendency or danger for others

Positive symptoms diagnosed in the schizophrenia group:

SZ1: no formal thought disorder, no delusions, no hallucinations

SZ2: formal thought disorder, paranoid thoughts, hallucinations

SZ3: formal thought disorder, no delusions, no hallucinations

Medication of the schizophrenia group:

SZ1: Lithium 450 – 0 – 450 mg, Quetiapine ret. 0 – 0 – 800 mg

SZ2: Clozapine 0 – 100 – 400 mg

SZ3: Valproate 1000 – 0 – 1000 mg, Clozapine 50 – 0 – 100 – 300 mg, Citalopram 20 – 0 – 0 mg, Fluanxol Depot 20mg 2%ig each 14d

Medication of the autism group:

The 3 patients with ASD received no medication.

Patients with schizophrenia were additionally tested for prepulse inhibition (PPI). SZ1 and 2 showed dysfunctional PPI while SZ3 showed low response rates excluding measuring startle responses.

iPSC reprogramming and differentiation

Fibroblasts from five individuals of each group (CTR, SZ, ASD) were isolated. Successful reprogramming was achieved from fibroblasts of three individuals per group, nine individuals in total. >100 independent clones were initially picked from each of the nine individuals. At least 10 independent iPSC clones were selected upon downregulation of exogenously introduced reprogramming factors and upregulation of endogenous stem cell markers based on RT-PCR and surface marker expression (TRA1-81, SSEA4). Three clones out of each individual were further expanded using the following criteria:

1. low degree of spontaneous differentiation

2. suitable proliferation rate

Since growth characteristics of differentiated neurons in single wells are quite different, any manual inspection of cellular assays in combination with low sample numbers needs to be excluded. As indicated in the figure legends, we expanded the number of data points to achieve robust assays.

For the basic experiments measuring neurite outgrowth and PSD95 cluster density (figure 3A and 4D), potential variations among individual donors, clones and differentiations were excluded as follows: Six independent differentiations with at least three differentiations per clone in total were applied to control for individual donors and differentiations. For normalization, clone CTR1 was included in each of the six independent differentiations. Clone variation was ruled out by including nine independent clones derived from nine individuals with three individuals (=clones) per group. Thus, three independent clones were included allowing for a group comparison. For the nine selected clones derived from nine patients, the full characterization program for expression of iPSC-, NPC-, and neuronal markers as well as of pluripotency and chromosomal integrity testing was applied.

At the example of one differentiation, transcriptomic analysis over all nine individual clones was performed (figure 2). The main finding that one clone from SZ2 showed deregulated expression of MHC II complexes was confirmed via quantitative immunofluorescence of one further independent differentiation using antibody Tü39 recognizing HLA-DR, -DP and –DQ (figure 2D).

Human skin biopsies were collected in F12/10% FCS. Tissue was dissociated in F12/0.2% trypsin, washed in F12/10% FCS and transferred to 10 cm Petri dishes. Once cells have adhered, DMEM/FCS was added for further cultivation and passaging. Fibroblasts were transduced with retrovirus expressing c-MYC, [KLF-4](https://de.wikipedia.org/w/index.php?title=Klf-4&action=edit&redlink=1), [OCT-4](https://de.wikipedia.org/wiki/Oct-4) and [SOX-2](https://de.wikipedia.org/wiki/Sox-2) (Addgene plasmids 13375, 13370, 13366, 13367) and reprogrammed according to published protocols [1]. hiPSC colonies were expanded on feeder layers of irradiated murine embryonal fibroblasts followed by expansion on Matrigel-coated plates (Corning Incorporated, New York, USA) in mTeSR^TM^1 medium (STEMCELL Technologies Germany GmbH , Köln, Germany).

For NPC generation, iPSCs were enzymatically dissociated using StemPro Accutase Cell Dissociation Reagent (Life Technologies GmbH, Darmstadt, Germany). 2 to 3x10^6^ hiPSC resuspended in STEMdiff^TM^ Neural Induction Medium (STEMCELL Technologies Germany GmbH , Köln, Germany) plus Y-27632 dihydrochloride (10 µM, Tocris, Bristol, UK) were seeded per single well of an AggreWell^TM^ plate (STEMCELL Technologies Germany GmbH , Köln, Germany). After incubation at 37 °C, 5 % CO_2_ for 5 days with daily medium change, embryoid bodies were harvested using 37µm Reversible Strainers (STEMCELL Technologies Germany GmbH, Köln, Germany) for plating. 6-well plates were pretreated with 0,002 % poly-L-ornithine in Dulbecco's phosphate-buffered saline, incubated for two hours at room temperature. (#P4957, Sigma-Aldrich, Munich, Germany) and washed. Subsequently, wells were treated with laminin solution (10 µg/ml in DMEM/F12), and incubated overnight at 37°C and 5 % CO_2_, (#L2020, Sigma-Aldrich, Munich, Germany). After washing, embroid bodies were plated into one well of a poly-L-ornithine/laminin coated 6-well plate, and cultivated for 7 days in STEMdiff^TM^ Neural Induction Medium (STEMCELL Technologies Germany GmbH , Köln, Germany) with daily medium change. Neural rosettes were selected using the STEMdiff^TM^ Neural Rosette Selection Reagent (STEMCELL Technologies Germany GmbH, Köln, Germany), resuspended in STEMdiff^TM^ Neural Induction Medium plus Dorsomorphin dihydrochloride (1 µM, Tocris, Bristol, UK), SB 431542 (10 µM, Tocris, Bristol, UK), recombinant Human Noggin Fc Chimera Protein (500 ng/ml, Bio-Techne GmbH, Wiesbaden-Nordenstadt, Germany) and cultivated in poly-L-ornithine/laminin coated 6-well plate. After the first passage, cultivation medium was changed to STEMdiff^TM^ Neural Progenitor Medium (STEMCELL Technologies Germany GmbH, Köln, Germany). NPCs were passaged up to passage 10.

For neuronal differentiation, NPC were incubated on poly-L-ornithine/laminin-coated dishes in 3N medium [2] plus bFGF (20ng/ml, Thermo Fisher Scientific, Rockford, USA) for 2 days. Subsequently, cells were transferred to poly-L-ornithine/laminin-coated cover slips and cultivated in 3N medium at a density of 5x10^4^/cm^2^ for up to 8 weeks. Medium was changed twice a week. For functional assays, medium was changed to BrainPhys^TM^ Neuronal Medium (STEMCELL Technologies Germany GmbH, Köln, Germany) plus supplements for the last two weeks of differentiation. STEMdiff™ Definitive Endoderm Kit (STEMCELL Technologies Germany GmbH, Köln, Germany) was used for endodermal differentiation of iPSCs. Mesodermal differentiation was achieved using PSC Cardiomyocyte Differentiation Kit (Thermo Fisher Scientific, Rockford, USA). Immunohistochemistry for the assessment of stem cell and neural phenotypes was performed as detailed below.

Number of clones, differentiations, biological and technical replicates for the main experiments

Fig 2C: PSD95 cluster

| Number of individuals | Number of independent differentiations | Number of technical replicates |
| --- | --- | --- |
| 9 (3 individuals per group) | 3 for each individual | 40 neuritic segments for each differentiation |

Fig 3D: neurite outgrowth

| Number of individuals | Number of independent differentiations | Number of technical replicates |
| --- | --- | --- |
| 9 (3 individuals per group) | Min of 3 for each individual | >150 cells for each differentiation |

Fig 3E: neurons with neurites

| Number of individuals/clones | Number of independent differentiations | Number of technical replicates |
| --- | --- | --- |
| 9 (3 individuals per group) | Min of 3 for each individual | 25 images with at least 25 cells for each image |

Fig 3F: drugs

| Number of individuals/clones | Number of independent differentiations | Number of technical replicates |
| --- | --- | --- |
| 9 (3 individuals per group) | 3 for each individual | 81 images from three independent differentiations |

Fig 4: calcium measurements

| Number of individuals | Number of independent differentiations | Number of technical replicates |
| --- | --- | --- |
| 9 (3 individuals per group) | 3 for each individual | >60 cell per individual |

Fig 5A,B: transcriptomic analysis

| Number of individuals | Number of independent differentiations | Number of technical replicates |
| --- | --- | --- |
| 9 (3 individuals per group) | 1 for each individual | Next generation sequencing of whole transcriptomes |

Fig 5F,I: MHC class II expression

| Number of individuals | Number of independent differentiations | Number of technical replicates |
| --- | --- | --- |
| 4 (3 independent clones of 1 individual SZ, 3 individuals with 1 clone/individual CTR) | >3 for each clone | >100 per clone (NPC)  >50 per clone (neurons) |

Array-CGH analysis

DNA isolation from cell pellets was performed using DNeasy Blood&Tissue Kit (Qiagen GmbH, Hilden, Germany). The Human Genome CGH 400K Microarray (Agilent Genomics) was used for detection of genome-wide DNA copy number variations in all analyzed samples with a resolution of >50kb. Small aberrations were further analyzed only if data quality and probe numbers proved to be sufficient in the region of interest. The gene list only considered gene annotation according to Software Agilent Cytogenomic Version 3.0.6.6.

Immunocytochemistry

For immunocytochemical analyses, the following antibodies were used: mouse monoclonal anti-gephyrin (mAb7a; 1:100; Synaptic Systems, Göttingen, Germany), rabbit polyclonal anti-PSD-95 (1:200; New England Biolabs GmbH, Frankfurt, Germany), mouse monoclonal β-III-tubulin (1:250,STEMCELL Technologies Germany GmbH, Köln, Germany), StemLightTM Pluripotency Surface Marker Antibody Kit (SSEA4, Tra1-81, 1:500, Cell Signaling Technology, MA, USA), mouse Cardiac Troponin T monoclonal antibody (13-11) (1:200, Thermo Fisher Scientific, Rockford, USA), polyclonal goat anti-human Sox17 antibody (1:250, R&D Systems, Minneapolis, USA), mouse monoclonal ZO-1 (1A12) antibody (1:150, Thermo Fisher Scientific, Rockford, USA), polyclonal rabbit anti-Pax-6 antibody (1:300, BioLegend, CA, USA), mouse monoclonal anti-Nestin antibody (1:1000, Synaptic Systems, Göttingen, Germany), polyclonal rabbit anti-SOX1 antibody (1:500, Abcam, Cambridge, UK) mouse monoclonal anti-VGLUT 1 antibody (1:300, Synaptic Systems, Göttingen, Germany). Cells were fixed with 4% paraformaldehyde/PBS for 10 min. After blocking and permeabilizing for 30 min with 0.2% Triton X-100 in PBS containing 5% Normal Goat Serum (ab7481; Abcam, Cambridge, UK) , cells were incubated with primary antibody at 4°C overnight. Subsequently, cells were washed three times using PBS before the secondary antibody (1:600 in 1xPBS; AlexaFluor647/AlexaFluor488/Cy3/Cy5-coupled donkey anti-goat, goat anti-mouse or goat anti-rabbit; Dianova GmbH, Hamburg, Germany) was added for 2 hrs at room temperature. Nuclei were stained using Hoechst 33258 (1:1000 in PBS; Sigma-Aldrich, St. Louis, USA).

| Antibodies |  |  |  |  |
| --- | --- | --- | --- | --- |
|  | **spezies** | **Manufacturer** | **Catalogue Number** | **Dilution** |
| β-III-tubulin (AA10) | mouse IgG2a | STEMCELL Technologies | 60100 | 1:250 |
| β-III-tubulin | rabbit | Synaptic Systems | 302302 | 1:1000 |
| Gephyrin | mouse IgG1 | Synaptic Systems | 147021 | 1:100 |
| Nestin (JP63) | mouse IgG | Synaptic Systems | 312011 | 1:1000 |
| NeuN | mouse IgG1 | Millipore | MAB377 | 1:500 |
| Pax-6 | rabbit | BioLegend | PRB-278P | 1:300 |
| PSD95 | rabbit | Abcam | 18258 | 1:1000 |
| SOX1 | rabbit | abcam | ab22572 | 1:500 |
| SSEA4 (MC813) | mouse IgG3 | Cell Signaling | 4755 | 1:1000 |
| Synaptophysin | rabbit | Synaptic Systems | 101002 | 1:500 |
| TRA-1-81 | mouse IgM | Cell Signaling | 4745 | 1:1000 |
| VGLUT1 | mouse IgG1 | Synaptic Systems | 135511 | 1:300 |
| ZO-1 | mouse IgG1 | Thermo Fisher Scientific | 339100 | 1:150 |

Proteomic analysis

For proteomic analysis a single well was plated with 1 x 10^5 cells of a single clone, of all 9 subjects, and differentiated for 6 weeks. Before harvest, cells were washed with PBS to remove media contaminants, and scraped without trypsin and resuspended in 35 ul 2%SD and 100 mM Tris pH 8.8, and stored at <70 ^0^C. Sample was incubated in 2x Laemmli SDS buffer containing 3 uL of 30% acrylamide for 45 min, then heated at 95^0^C for 5 min, loaded on a precast gel (Novex™ WedgeWell™ 10% Tris-Glycine Mini Gels) and run at 120V for approximately 1cm into the gel. The gel was fixed overnight, washed 4x15min in water, and briefly stained with colloidal Coomassie for 2-3 min. The sample-containing gel piece was cut into small blocks of approximately 1mm^3^ and collected in a 96-well filterplate (MultiScreen HTS, HV filter plate; Merck) with a Deepwell 96-Well collection plate (Eppendorf, Hamburg, DE). The gel blocks were destained in 50% acetonitrile/50% 25 mM NH_3_HCO_3_ and dehydrated in 100% acetonitrile. 100uL 50mM NH_3_HCO_3_ containing 0.67 µg Trypsin (Promega Sequence-grade) was added to the gel and incubated overnight at 37^0^C. Tryptic peptides were collected by centrifugation at 200xg for 1 min into the collection plate. The gel was further extracted once with 150 µL 0.1% TFA/50% acetonitrile and once with 0.1% TFA/80% acetonitrile, both collected by centrifugation in the deep-well plate. Peptides were transferred to Eppendorf tubes, dried in a speedvac, and stored at -20 ^0^C until mass spectrometry analysis.

Tryptic peptides were re-dissolved in 15 µl of 2% acetonitrile/0.1% formic acid, and injected into an Ultimate 3000 LC system (Dionex, Thermo Scientific) with a 5 mm Pepmap 100 C18 trap column (300 μm id, 5 μm particle size, Dionex). Peptide fractionation was performed on a 200 mm Alltima C18 homemade column (100 μm ID, 3 μm particle size), using a linear gradient of increasing acetonitrile concentration in 0.1% formic acid from 5% to 22% in 88 min, to 25% at 98 min, to 40% at 108 min, and to 90% at 110 min. The flow rate was 5 µl/min. Peptides were electro-sprayed into the TripleTOF 5600 mass spectrometer (Sciex) using an ion spray voltage of 2.5 kV and an interface heater temperature of 150°C. The MS survey scan range was *m/z* 350–1250 acquired for 200 ms. The top 20 precursor ions were selected for 100 ms per MS/MS acquisition. Dynamic exclusion was 16 s. Rolling CID function was activated. The collision energy spread was 15 eV. Mass spectra were searched against the human database (Uniport_2015-2) using MaxQuant. The MaxQuant default setting was used, except that Label Free Quantification (LFQ) was set to min. ratio count of 1, propionamide was used as fixed modification, and match between run was activated.

Significantly regulated proteins in any of the three group-wise comparisons were identified with ANOVA, and the issue of multiple testing was taken into consideration by converting ANOVA p-values into q-values [3] and resulting FDR is reported in the results.

1. Takahashi, K., et al., Induction of pluripotent stem cells from adult human fibroblasts by defined factors. Cell, 2007. **131**(5): p. 861-72.

2. Shi, Y., et al., Human cerebral cortex development from pluripotent stem cells to functional excitatory synapses. Nat Neurosci, 2012. **15**(3): p. 477-86, S1.

3. Storey, J.D. and R. Tibshirani, Statistical significance for genomewide studies. Proc Natl Acad Sci U S A, 2003. **100**(16): p. 9440-5.
